# Unraveling a Case Where the NAC Approach Fails—MD and QM Modeling of Regioselective *cis*-Stilbene Oxide Hydrolysis by Engineered Epoxide Hydrolases

**DOI:** 10.1021/acs.jcim.6c02029

**Published:** 2026-09-22

**Authors:** Elvira Bombino, Hesam Arabnejad, Baoyan Liu, Peter A. Jekel, Xiang Sheng, Dick B. Janssen, Fahmi Himo, Hein J. Wijma

**Affiliations:** † Biotransformation and Biocatalysis, Groningen Biomolecular Sciences and Biotechnology Institute, University of Groningen, Nijenborgh 4, 9747AG Groningen, The Netherlands; ‡ Department of Chemistry, Arrhenius Laboratory, 7675 Stockholm University, 10691 Stockholm, Sweden; § State Key Laboratory of Engineering Biology for Low-Carbon Manufacturing, Tianjin Institute of Industrial Biotechnology, 165087 Chinese Academy of Sciences, Tianjin 300308, P. R. China

## Abstract

Molecular dynamics (MD)-based methods to predict the enantioselectivity or product specificity of enzymes typically use a near attack conformation (NAC) approach. The computational efficiency of this ground-state approach, taking minutes to hours, allows to in silico screen thousands of enzyme variants. During the computational design and screening of new variants of limonene epoxide hydrolase for improved stereoselectivity, it was noticed that, while this approach gave satisfactory prediction accuracy for most substrates, the enantioselectivity predictions were far less accurate for variants designed for *cis*-stilbene oxide. Here, to investigate whether inaccurate predictions with the NAC-based approach were typical for this substrate, first, a larger data set was generated by experimentally testing and computationally predicting the enantioselectivity of an additional 18 enzyme variants for *cis*-stilbene oxide. Subsequent MD simulations confirmed that a NAC analysis resulted in inaccurate enantioselectivity predictions throughout the data set. Density functional theory (DFT) calculations using the B3LYP-D3­(BJ) functional, with an active site cluster model of >300 atoms, provided a rationale for why the NAC approach gave poor predictions with *cis*-stilbene oxide. The aromatic rings of the substrate influence the nucleophilic attack on the epoxide group, by providing a blend of stereoelectronic activation and steric hindrance to the transition state, which is not considered in the NAC approach. With this substrate, the DFT accurately predicted the enantioselectivity. The results offer valuable insights into the applicability of the NAC approach for predicting enzyme selectivity, suggesting that more expensive methodologies, which do explicitly consider the transition state structure, can also become required when bulky or stereoelectronically activating groups are bound to the reacting atoms.

## Graphical abstract

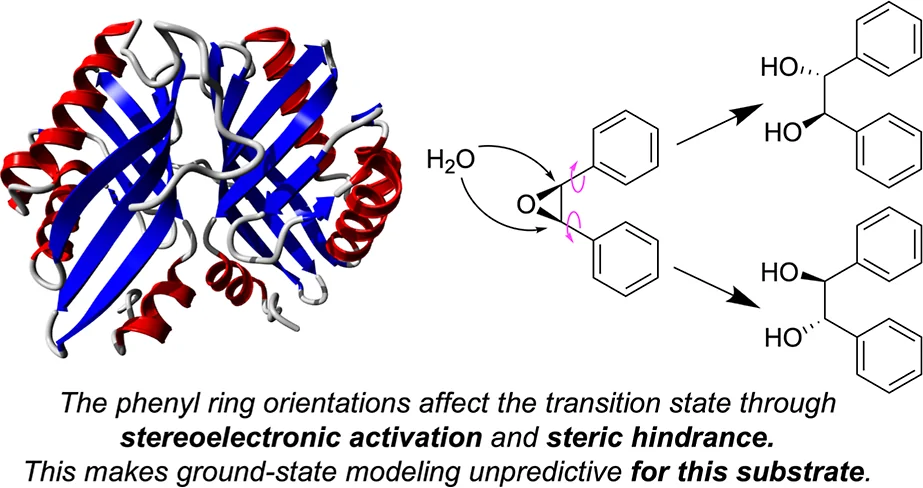

## Introduction

Enzyme engineering often is a key step in the development of eco-friendly biocatalytic processes. Important targets are the enhancement of operational stability and tuning of the catalytic selectivity to meet bioprocess requirements. In the case of applications in pharmaceutical synthesis, high enantioselectivity frequently is an essential property.^1–3^

Chiral epoxides and 1,2-diols are building blocks for the synthesis of bioactive compounds, including pharmaceuticals.^4,5^ They can be prepared by stereoselective epoxide ring opening, either through homogeneous catalysis using chiral metal complexes,^6^ or enzymatically using epoxide hydrolases or halohydrin dehalogenases.^7^ Epoxide hydrolases are a diverse group of enzymes that are considered attractive for stereoselective synthesis due to their capacity to transform a wide range of epoxides to chiral products, either by asymmetric hydrolysis of *meso*-epoxides or via kinetic resolution of racemates.^8,9^

Two important groups of epoxide hydrolases can be distinguished. The α/β-hydrolase fold group encompasses mostly monomeric enzymes.^10^ These possess a nucleophilic aspartate that attacks an epoxide carbon with the formation of an hydroxyalkyl-enzyme intermediate, which is subsequently hydrolyzed to release the corresponding diol. The other group includes enzymes related to limonene epoxide hydrolase (LEH). The first enzyme of this LEH group was discovered some 30 years ago in *Rhodococcus erythropolis*.^11^ These enzymes are homodimers with subunits of approximately 155 amino acids. The epoxide hydrolysis reaction proceeds by nucleophilic attack of a water molecule on an epoxide carbon atom, without the formation of a covalent enzyme–substrate intermediate.^11,12^ Of the two groups, LEH enzymes have recently been explored for stereoselective transformations.

The *R. erythropolis* limonene epoxide hydrolase (PDB 1NWW,^12^ Uniprot Q9ZAG3) and its homologues display a concerted reaction mechanism for the opening of the epoxide ring by water (Figure 1). The nucleophilic water molecule is positioned through a network of hydrogen bonds involving an asparagine (Asn55), a tyrosine (Tyr53), and a conserved aspartate (Asp132). The aspartate activates the water molecule for nucleophilic attack by abstracting one of the protons. The epoxide oxygen, which is the leaving group in the S_N_2 reaction, is protonated by a second conserved aspartate (Asp101). An arginine (Arg99) orients the carboxylate groups of the two aspartates in a suitable position for the reaction to occur in a concerted fashion.^13,14^ LEH is very efficient in the hydrolysis of its natural substrate limonene epoxide, displaying high turnover numbers^11,13,14^ with a *k*_cat_ of 23 s^–1^, but its substrate range is limited and its enantioselectivity for non-native substrates is too low for application in practical synthesis.

**Figure 1:**
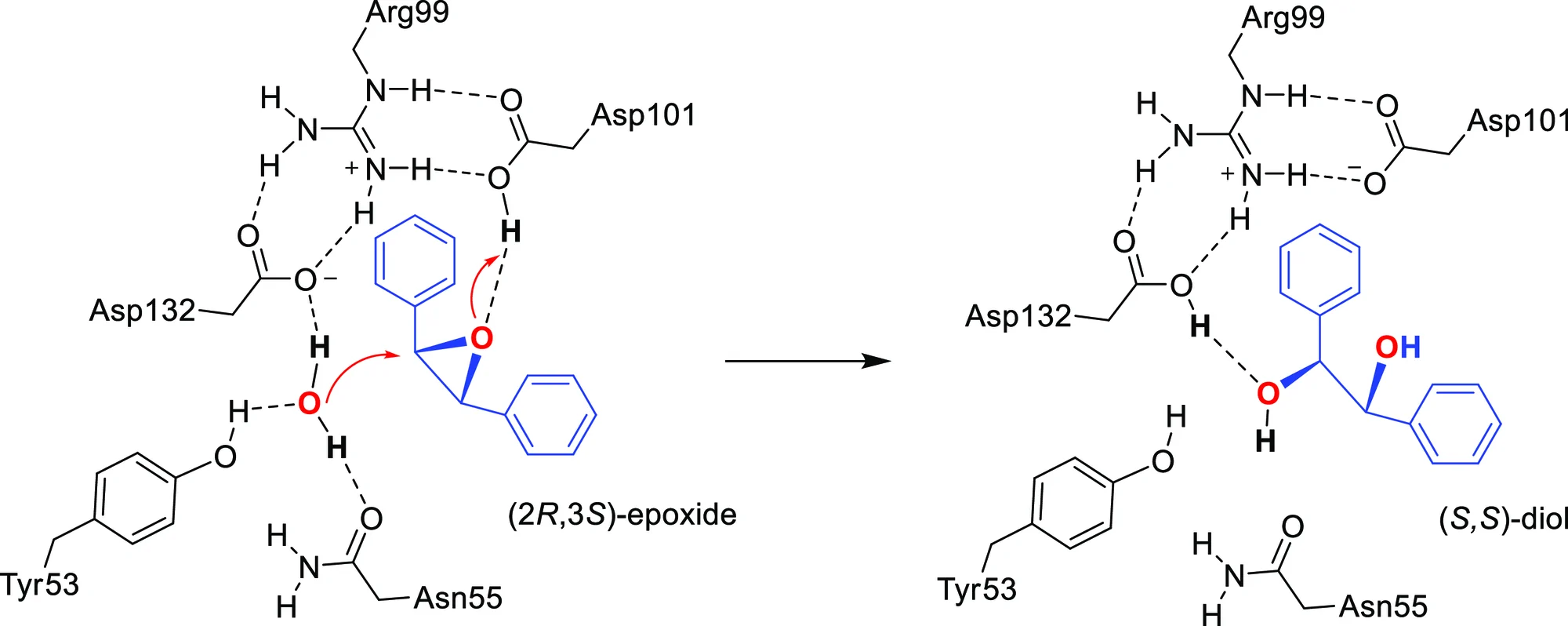
Mechanism of LEH-catalyzed hydrolysis of *cis*-stilbene oxide. The nucleophilic water is activated by Asp132 and the leaving group is protonated by Asp101, both facilitated by Arg99. The reaction proceeds with inversion of configuration at the chiral carbon atom that is attacked, converting the prochiral meso compound *cis*-stilbene oxide to the corresponding (*R,R*)- or (*S,S*)-alcohol, depending on the regioselectivity of water attack. The figure shows regioselective attack on the (*R*)-configured carbon atom of the meso compound, leading to an (*S,S*)-diol.

In view of these attractive properties of LEH for stereoselective biocatalysis, there is an interest in discovering better variants through screening or protein engineering. In the past decade, new LEH-like epoxide hydrolases were identified by screening hot spring metagenomic libraries.^15^ These variants, however, had similar low enantioselectivity for non-natural substrates as previously discovered wild-type variants. Directed evolution and computational design studies, to improve enantioselectivity of LEH family members, gave good results.^16–19^ Although directed evolution is an established approach for enzyme activity engineering,^20,21^ it is often difficult to implement when targeting enantioselectivity since it requires high-throughput tools for chirality screening of large numbers of enzyme variants, which is associated with significant cost and time efforts.^19,22^ Therefore, use of in silico methods such as bioinformatics and computational design are in demand.

Computational enzyme engineering has gained considerable momentum due to increasingly powerful hardware and algorithms. Ideally, computational tools should predict optimal variants in a single step, without a need for iterative mutagenesis followed by repetitive experimental testing and improvement. The approaches include structure-based computational redesign employing biophysical energy functions, and Monte Carlo search algorithms.^23,24^ The use of such procedures for computational prediction of enzyme variants with increased thermostability,^25–28^ activity, and enantioselectivity^29,30^ has been reported.

Some of us have developed a protocol called CASCO, which includes the use of the Rosetta framework with its energy function^23^ to obtain primary designs in a single dock and design step, followed by computational screening for the desired enantioselectivity by molecular dynamics simulations.^30^ This way, different LEH variants with modified enantioselectivity were obtained, including enzymes with enantiocomplementary selectivity for cyclopentene oxide and *cis*-2,3-buteneoxide. With such meso compounds, the design process optimizes the protein environment for binding the substrate in a conformation that promotes water attack either on the *R*-configured or on the *S*-configured epoxide carbon (Figure 1), which leads to an (*S,S*)-diol or (*R,R*)-diol, respectively. In total 37 *proSS* variants and 20 *proRR* variants were designed.^29,30^ When evaluated experimentally for asymmetric conversion of their aliphatic target substrates, 52 of the 57 variants were active. The large majority, 45 out of 52, showed the predicted enantioselectivity. Thirteen of these variants were highly enantioselective for production of the targeted enantiomer with an enantiomeric excess (e.e.) of >75%.^29,30^ However, 7 variants displayed the wrong enantioselectivity, suggesting some flaws in the in silico screening process.

Most incorrect predictions concerned diols produced from the aromatic substrate *cis*-stilbene oxide. Of the 10 variants designed for this substrate, 2 were essentially inactive (<1% activity compared to the most active variant) but appeared to produce the wrong diol enantiomer, 2 had very low activity, and 2 variants were active but certainly produced the wrong diol enantiomer. This indicated that most variants designed for *cis*-stilbene oxide hydrolysis did not perform as expected. Another issue specific for this substrate was that one of the mis-predicted (*R*,*R*) variants appeared to be the best (*S,S*)-diol producing variant (52A, e.e. 97%) and also the third most active variant (43% compared to the most active variant, Table 1).^29^

**Table 1:**
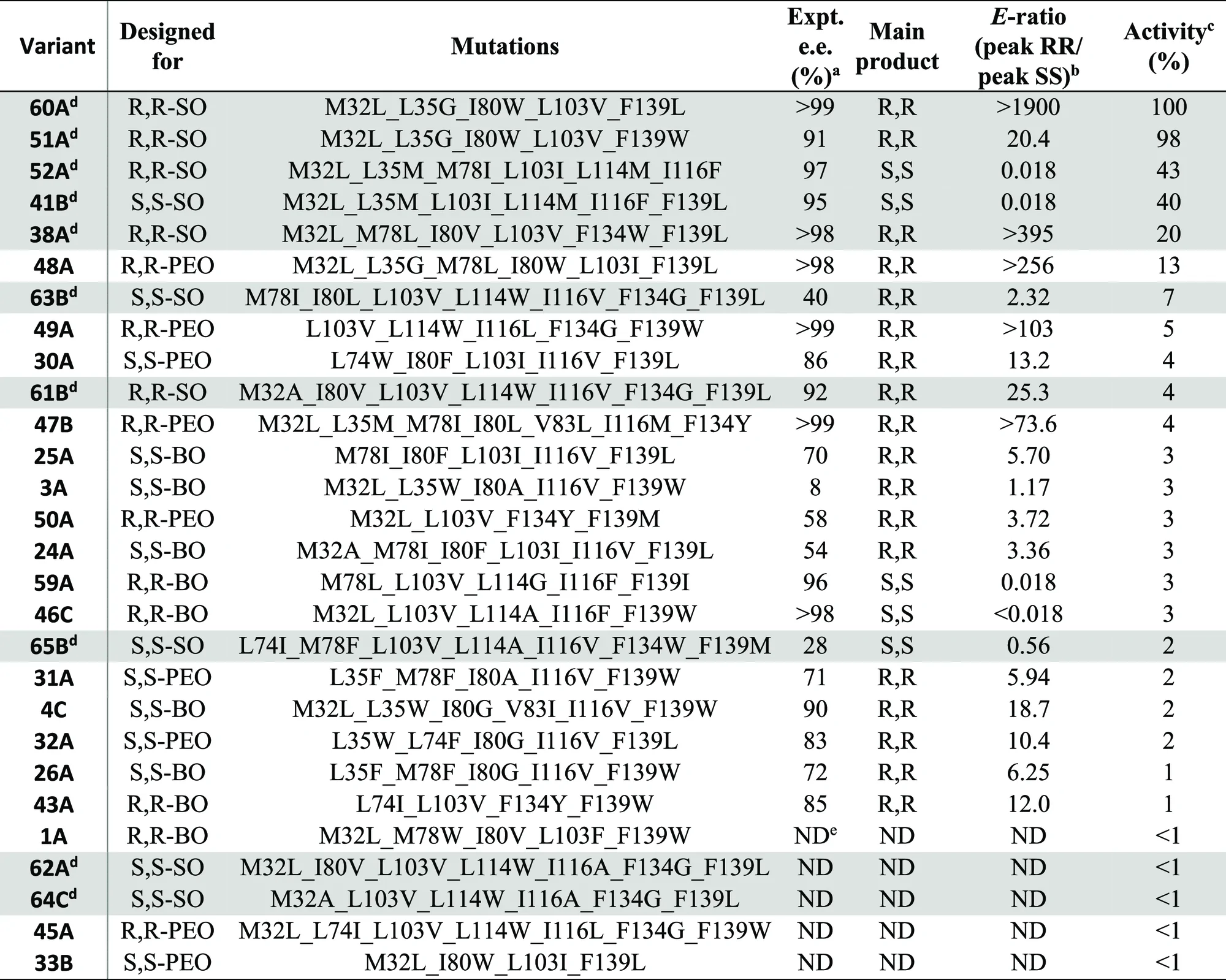
Enantioselectivity and Activity of Redesigned LEH Variants with *cis*-Stilbene Oxide^a^

a All activities of variants with a white background are newly reported in this study. Part of the results were reported earlier^29^ and are shown here for comparison, with a gray background; percentage enantiomeric excess based on HPLC areas.

b The mutants were initially designed for improving selectivity of LEH for substrates PEO (cyclopentene oxide), BO (butene oxide) or SO (*cis*-stilbene oxide) for both *R,R*-selective and *S,S*-selective variants.

c Relative activity compared to the most active variant (60A). Mutants are ordered by activity.

d Reported earlier.

e ND, not determined due to low activity.

A better understanding of the origins of the incorrect enantioselectivity predictions for *cis*-stilbene oxide could contribute to better predictions in future enzyme design projects. An aim of the current study was therefore to explore the enantioselectivity determinants for *cis*-stilbene oxide using more accurate methods. We increased the experimental data set by measuring the enantioselectivities of 18 additional LEH variants with *cis*-stilbene oxide and explored whether selectivity predictions could be improved by better conformational sampling through a larger number or longer MD simulations. Furthermore, quantum mechanical (QM) calculations using density functional theory (DFT)^14^ were performed on LEH-P, a thermostable variant with the same enantioselectivity as the WT, and on the most enantioselective *R,R-* and *S,S-*mutants (60A, 52A and 41B, Table 1). The results indicate that a NAC approach, which only evaluates the ground state, is insufficient for the complicated situation of enantioselective hydrolysis of *cis*-stilbene oxide, where the phenyl rings can provide both stereoelectronic activation and steric hindrance during the transition state, depending on their orientation. The DFT-calculated relative energy barriers were in excellent agreement with experimental data, which demonstrates that this methodology can provide good enantioselectivity predictions on such a challenging case.

## Materials and Methods

### Production and Characterization of Mutant Enzymes

Mutations were introduced in the LEH gene by QuikChange site-directed mutagenesis, using as the template a pBAD-derived expression vector encoding LEH-P (*T*_m,app_ = 70 °C, PDB: 4R9K).^31^ Mutagenesis, transformation, plating, and sequencing were done in microtiter plate format as previously described.^29^ After confirming the sequence, LEH variants were expressed and isolated by His-tag affinity chromatography with Ni-NTA agarose resin. This gave samples of purified enzymes with a typical yield of 50–150 mg per liter of culture. Protein concentrations were determined by the Bradford assay, and purity was checked by SDS-PAGE. The LEH variants could be stored for more than 2 years at −80 °C without loss of activity.

Activity assays were performed by incubating 6.4 mg of enzyme (final concentration 320 μM) with 10 mg of *cis*-stilbene oxide (final concentration 50 mM) for 1 h at 30 °C in 50 mM HEPES, pH 8.0, in a total reaction volume of 1 mL. Samples were analyzed by HPLC on a Luxcellulose-3 column (Phenomenex, Utrecht, The Netherlands). The mobile phase was heptane/2-propanol (90/10) with a flow rate of 1 mL min^–1^. Formation of (*R,R*)- and (*S,S*)-1,2-diphenylethane-1,2-diol was detected at 254 nm and enantiomeric excess was calculated from product concentrations as described by Arabnejad et al.^29^

### Molecular Dynamics Simulations

Starting with the thermostable LEH scaffold (PDB: 4R9K), Rosetta’s enzyme_design application (Rosetta version 60351) was used to model the 23 active LEH-variants with *cis*-stilbene oxide docked in the active site in *proRR* and *proS*S conformations in subunit A and B, respectively. Rosetta simultaneously mutates the required residues and optimizes the binding pose of the substrate in the active site.^24,32^ The substrate was placed in a catalytically optimal geometry, with the relevant epoxide carbon aligned between the nucleophilic water from the crystal structure and the epoxide oxygen, which itself was near Asp101 for facile protonation.^29,30^ For each of the 23 variants, Rosetta was instructed to introduce the 4–7 mutations, while both optimizing the conformation of the active site and maintaining the catalytically productive geometry of the substrate. Accurate sampling of substrate conformations was obtained by first creating a random library of 1000 rotamers with different dihedrals using YASARA. The dihedral angles were assigned using Yasara’s staggered method, which selects angles from energetically favorable staggered conformations. The rotamers were scanned for duplicates, deleting rotamers with less than 0.1 Å RMSD from an earlier encountered rotamer. The resulting 343 unique substrate conformations were used for docking by Rosetta (options: “-nstruct 200 -extra_res_fa SUB.params WAT.params -cst_predock -cst_design -detect_design_interface -cst_min -chi_min -bb_min -use_input_sc -soft_rep_design -extrachi_cutoff 1 -design_min_cycle 5 -ex1:2 -ex2:2 -ex1aro:2 -ex2aro:2”). For the MD simulations, either just one Rosetta-modeled E–S complex was used per variant or 200 of such E–S structures were used for each variant.

Some of the enzyme–substrate complexes were generated independently using Boltz-2.^33^ To this end, the amino acid sequence of 4R9K was used as input (with the relevant mutations, Table 1). The dimeric structures were predicted with the products instead of the substrates to decrease the number of ligands to be modeled in this step from four (two substrates and two water molecules) to two. The command line options for Boltz-2 were --use_msa_server --diffusion_samples 50 --use_potentials. From the resulting 50 predicted structures with bound product, the best 45% (as ranked by Boltz-2) were further evaluated using geometric criteria that selected for catalytically competent poses of the corresponding substrate (namely the sum of the distance between one hydroxyl group and Asp101 and the average distance of the other hydroxyl group to Asn55, Tyr53, and Asp132, while also the dihedral over the four most central carbon atoms had to be >−10° to prevent generation of the *trans*-stilbene oxide). The structure with the best geometry score was converted to one that had the original substrates and waters using local energy minimization steps under Yasara, in which only the substrates, the water molecules, and nearby hydrogen atoms of the enzyme were allowed to change conformation (script available upon request). Initial experiments with LEH-P, using either the *RR* and *SS*-product as input, resulted in indistinguishable substrate-bound structures and therefore all production modeling was done using the experimentally known preferred product for each variant. From the resulting models, the floppy termini were removed by deleting all N- and C-terminal residues with a pLDDT score of less than 75. Without further optimization, this consistently resulted in final structures with the same start and end residues as in LEH structure 1NWW.^12^

All MD simulations were performed with Yasara,^34^ using the Yamber3 force field, which is a derivative of Amber99.^35^ Partial atomic charges for the substrate atoms were assigned using the restrained electrostatic potential method (RESP) with Gaussian09 software.^36^ The time step was 1.25 fs and the list of electrostatic interactions was updated every 2 timesteps. An extensive set of high-throughput type molecular dynamics simulations was performed on the 23 redesigned LEH variants. After energy minimization of the structure, a gradual warming up from 5 to 298 K was performed. For the longest MD simulations, the warming up was done in 30 ps while for ultrashort (10 ps) MD simulations the warming up was done in 3 ps. Such ultrashort MD simulations can be used effectively for screening for enantioselectivity.^29,30,37,38^ For these ultrashort MD simulations, the following equilibration lasted 2 ps and the production phase 5 ps, with snapshots taken at 5 and 10 ps. For longer MD simulations, the equilibration lasted 2 ns, the production phase was 20 ns, and snapshots were taken every 10 ps. For the longest MD simulations, the initial equilibration lasted 200 ns, the production phase 300 ns, and snapshots were recorded every 100 ps.

To improve conformational sampling, multiple independent MD simulations were run per enzyme–substrate complex, each with a different set of independently assigned initial atom velocities. For ultrashort MD simulations, 20 independent trajectories were generated (i.e., 20 × 10 ps). Thus, if 200 E–S complexes were taken, 4000 ultrashort MD simulations were run for each variant, with a total simulation time of 40 ns per variant. For the longer time scale MD simulations, just one starting structure was used per variant. From the 200 starting structures generated by Rosetta, a structure was picked based on its low overall energy and its minimal violations of the geometric constraint, as reported in the Rosetta generated output file with scores. For each variant structure, 5 independent simulations of 22 ns were run with independently assigned initial atom velocities, giving a total simulation time of 100 ns. For four variants, 5 trajectories of 500 ns were carried out, with a total simulation time of 2.5 μs per variant.

While MD simulations were running, NACs (Figure 2) were scored on the fly (Figure 3) to more accurately determine their frequency than possible from the snapshots. For the 20 × 10 ps trajectories, the geometry was recorded every 20 fs, resulting in 5000 geometry measurements per simulated structure (namely 20 [trajectories] × 5000 [fs of production phase per trajectory]/20 [fs]), far more than possible from the 40 snapshots. For the 5 × 20 ns trajectories, this sampling was done every 200 fs resulting in 500,000 geometry measurements per variant while for the 5 × 500 ns MD simulations one geometry determination per 2 ps resulted in 750,000 geometry measurements per simulated variant. The enantioselectivity was calculated from the resulting NAC frequencies as described below (eq 1).

**Figure 2:**
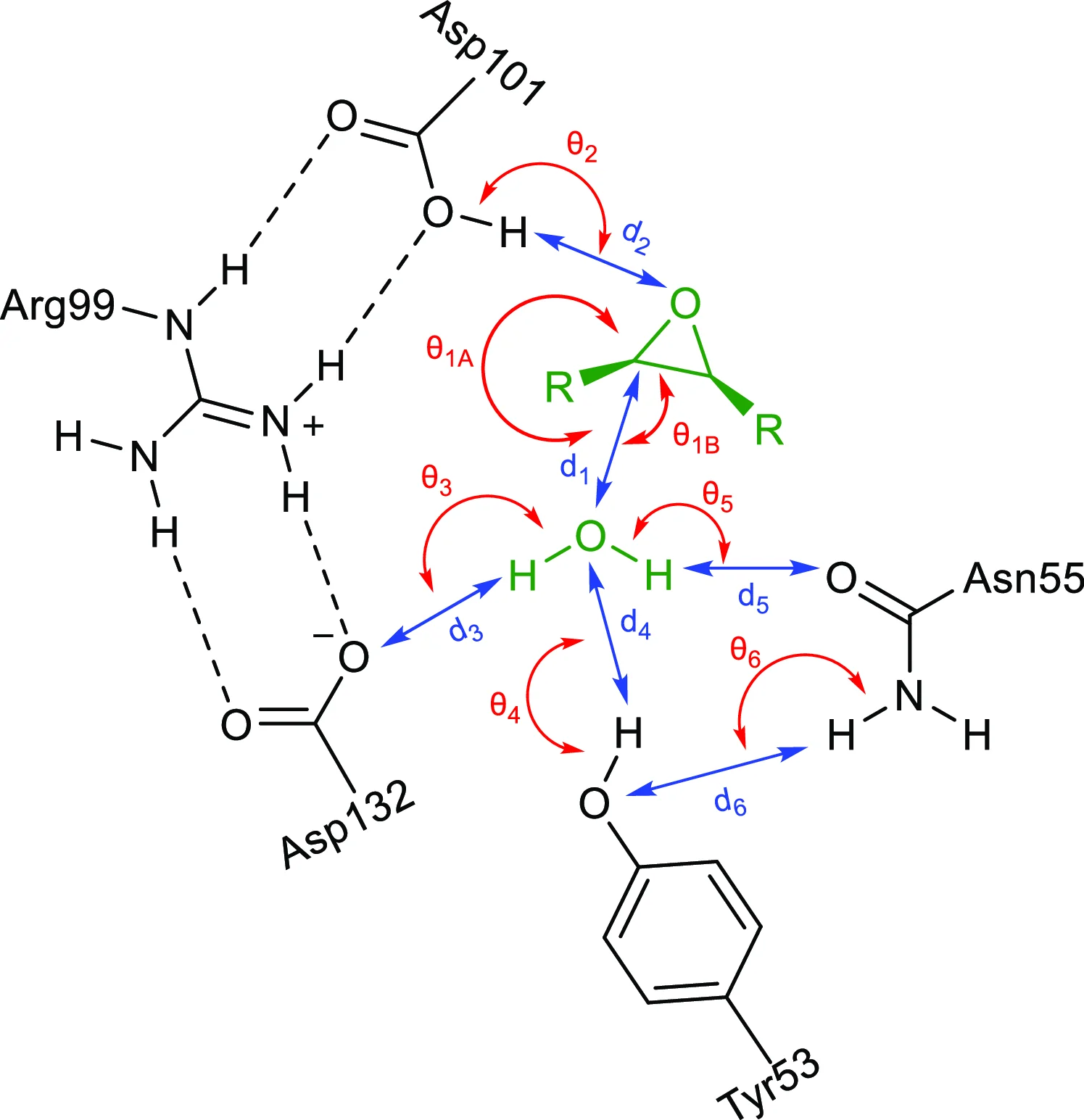
NAC criteria for stilbene oxide hydrolysis. Angle and distance criteria were defined as follows: for the H-bonds: θ_2_–θ_6_ = 120–180° and *d*_2_–*d*_6_ = 0–3.50 Å. For the nucleophilic attack angle: θ_1A_ = 128–163°, θ_1B_ = 99.5 ± 17.5° and *d*_1_ = 0–3.22 Å. The substrate and the reacting water molecule are shown in green.

**Figure 3:**
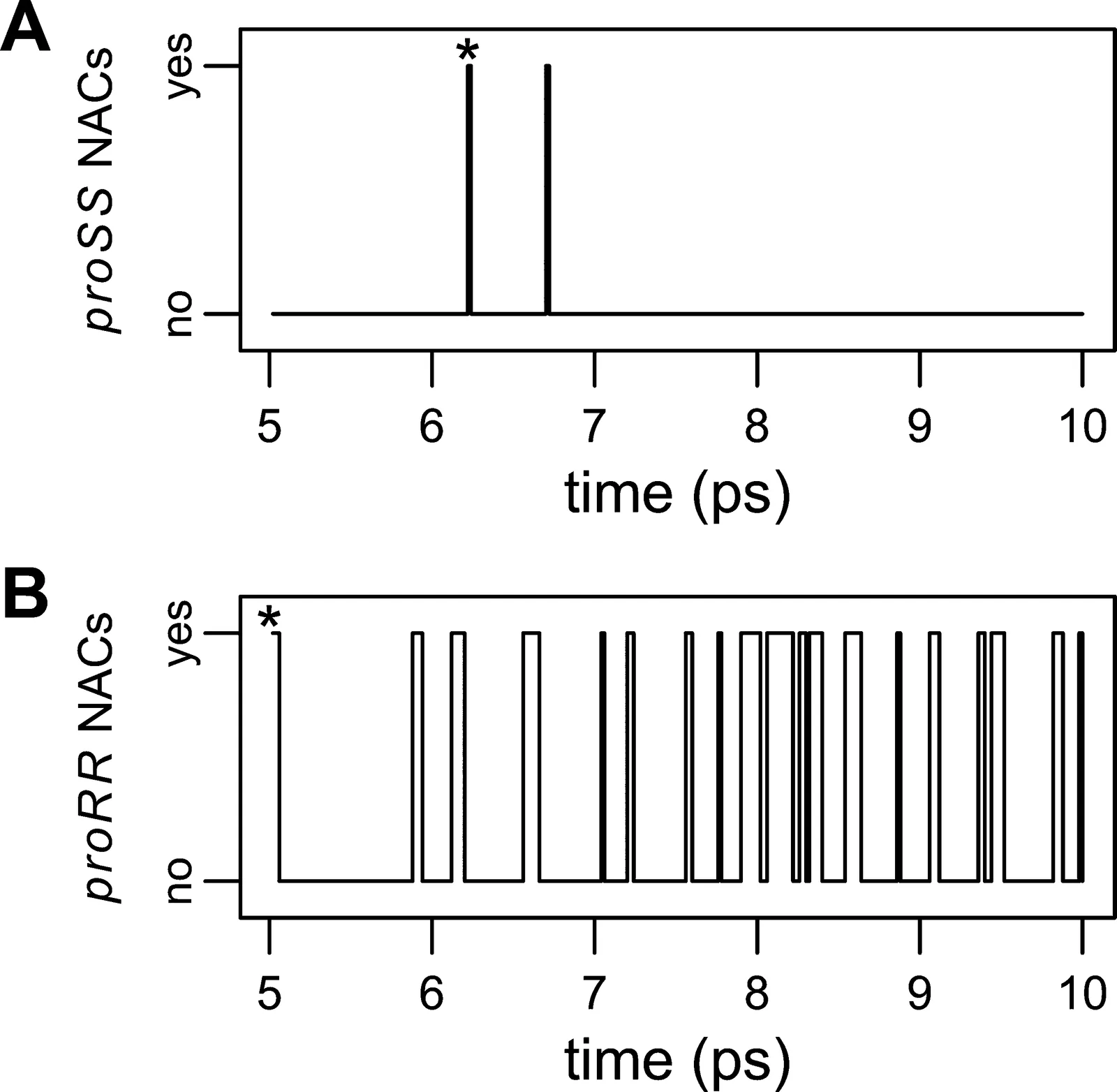
Dynamic exchange of NACs at the sub picosecond time scale. The presence of *proSS* (panel A) and *proRR* NACs (panel B) during the same simulation is plotted versus time. Displayed is the production phase of the first 10 ps trajectory of 60A (which used the Rosetta starting structure). The asterisk signs (*) indicate the time at which the additional snapshots displayed in Figure S1 were taken.

As in the previous study,^29^ NACs (Figure 2) were defined as conformations meeting a standard set of geometric criteria for angles and distances between substrate atoms and catalytic residues that were similar to those found for the transition state of the reaction (namely with angles within ±17.5° as from those observed in an earlier QM study^13^ and distances of less than the van der Waals contact distances). More permissive types of NAC definitions have been developed^39–41^ for other enzymes, like cytochrome P450s. Based on the frequency of occurrence of *proRR* and *proSS* NACs (*proRR* NACs are for attack on the *S*-configured carbon atom of the meso epoxide, giving the (*S,S*)-diol by inversion of configuration during nucleophilic attack), the product enantioselectivity of the mutants was predicted using eq 1.

1 $${e.e.}^{pred}=\frac{{NACs}^{RR}-{NACs}^{SS}}{{NACs}^{RR}+{NACs}^{SS}}$$

The enantiomeric ratio of product formation (*E*) and the difference in transition state energy between the *proRR* and *proSS* reaction (ΔΔ*G* ^TS^) are related via eq 2.

2 $${\Delta \Delta G}^{TS}=-RT\ln\frac{\left[R,R‐diol\right]}{\left[S,S‐diol\right]}\;\text{or}\;{\Delta \Delta G}^{TS}=-RT\ln\left(E\right)$$

To allow for comparison with experimental data, where e.e. never exceeds >99% due to the experimental detection limits for the nonpreferred products, e.e.^pred^ values of >99% were also rounded to 99%. Similarly, for *E* the maximum and minimal predicted ratios were set to 199 and 1/199, which results in a maximum/minimum ΔΔ*G* ^TS^ of ±13.1 kJ/mol for the predictions.

### Quantum Chemical Modeling

Quantum mechanical calculations were performed using Gaussian16 ^36^ with the B3LYP-D3­(BJ) density functional method.^42–44^ All geometry optimizations were performed using the 6-31G­(d,p) basis set.^45^ At the same level of theory, frequency calculations were performed, as well as single-point energies employing the SMD solvation model,^46^ with the dielectric constant set to 4. Based on the optimized structures, more accurate single-point energy calculations were carried out using the 6-311+G­(2d,2p) basis set. All reported data are the single-point energies with the large basis set, corrected by solvation effects and zero-point energies, in accordance with previous calculations.^14^

A cluster model for LEH-P, the thermostable variant of LEH used as the template in this work, was designed based on its crystal structure (PDB 4R9K). The model consists of 319 atoms, comprising the substrate, key catalytic residues (Tyr53, Asn55, Arg99, Asp101, and Asp132), the crystallographic water molecule that acts as the nucleophile, and other residues forming the active site (Met32, Leu74, Phe75, Met78, Ile80, Val83, Leu103, Leu114, Ile116, Trp130, Phe134, and Phe139). Hydrogens were added manually to saturate truncated bonds. Following the previous studies, Asp101 was set to its protonated form, while Asp132 was set to its deprotonated form.^13,14,47^ As usual in the cluster approach, a number of atoms at the edge of the model were kept fixed during the geometry optimizations (see Supporting Information, Figure S4).^48–53^

As the crystal structures of the redesigned LEH variants are not available, the Rosetta enzyme_design application^54^ was employed to obtain structures of the enzyme–substrate complexes for the mutants. The structure of LEH-P was used as a template, and mutations and docking were performed simultaneously. Three mutants with high experimental selectivities were considered, namely 41B, 52A and 60A (see Table 1). Based on these structures, cluster models consisting of 354, 347, and 351 atoms, respectively, were constructed in a similar fashion as for LEH-P. Optimized structures of these models are given in the Supporting Information.

## Results and Discussion

### Experimental Characterization of LEH Variants

The first step in analyzing the accuracy of the in silico predictions for the enantioselectivity of LEH variants was to generate a larger data set. In a previous study, we characterized 28 variants of limonene epoxide hydrolase that were designed to convert cyclopentene oxide, 2,3-butene oxide, and *cis*-stilbene oxide to enantiopure diols.^29^ It was found that ten variants specifically designed and tested for enantioselective conversion of *cis*-stilbene oxide gave a poor match between experimental results and predictions (see above). To exclude that this was mere coincidence, a further 18 LEH variants that were designed for the other substrates were also characterized with regards to their conversion of *cis*-stilbene oxide to enantiomeric diols.

Catalytic activities of the 18 additional variants with *cis*-stilbene oxide were calculated from chiral HPLC peak areas of (*R,R*)- and (*S,S*)-diols produced in reaction mixtures with purified enzymes (Table 1). It appeared that 15 of these 18 variants were catalytically active, but only one had a high activity with *cis-*stilbene oxide. Of the three inactive variants, two were originally designed to convert cyclopentene oxide and one had been designed for 2,3-butene oxide. The fraction of active designs was similar to that in the set of variants designed for *cis*-stilbene oxide (namely 8/10) but the activity of these dedicated designs was clearly higher. On average, stilbene oxide designs had 8- to 15-fold higher stilbene oxide hydrolysis activity than designs for cyclopentene oxide or butene oxide and the 5 most active variants for *cis*-stilbene oxide were all designed for this substrate.

The same chiral HPLC data were used to calculate enantioselectivities. The LEH-P template converted *cis*-stilbene oxide mainly to the (*R,R*)-diol with an e.e. of 90% and showed an activity almost double (196%) of that of the most active design. The same (*R,R*)-diol enantiopreference was found for 18 of the 23 active redesigned variants. A very high (*R,R*)-enantioselectivity, giving the diol with >95% e.e., was observed for only 5 designs. Only 5 of the 23 active variants showed predominant formation of the (*S,S*)-product, but with 4 of those displaying an excellent e.e. of ≥95%.

### Molecular Dynamics Simulations

To examine whether MD simulations can explain the observed product enantioselectivities, including agreements and deviations between the observed and designed chirality of the formed diols, we determined for all 23 active variants the occurrence of reactive conformations along simulation trajectories. It is established knowledge that multiple shorter MD simulations give more complete conformational sampling than a single much longer trajectory because at the start of an MD simulation the starting structure will relax randomly into one of many possible local minima while transitions from one into another local minimum only rarely occur during the rest of the MD simulation.^55–58^

It is especially relevant for modeling enzyme reactivity through a NAC approach that reactive conformations are often rare and only found in a minority of the trajectories even when these MD simulations are started from the catalytically most ideal conformations that could be obtained by docking. For example, for a P450 with a relatively large bound substrate (testosterone), only four out of the 20 × 300 ns MD simulations displayed the NACs that correctly predicted its enantioselectivity (when using standard strict NAC criteria like used in this investigation); the other trajectories sampled unproductive binding modes with (virtually) zero NACs.^41^ A systematic study on four dehalogenases with 47 different substrates (mostly hydrophobic with 4 to 21 heavy atoms) found that single long MD simulations (at the time 22 ns was long) gave poor predictions because the catalytically productive conformations were often missed, with 41% of the converted enantiomers giving zero NACs.^37^ The same study found that the structural relaxing at the start of the MD simulations mostly took less than 5 ps, which can be rationalized since carbon atoms at room temperature move at an average speed of 2.5 Å/ps, enabling the initial catalytically productively bound starting structures to rapidly relax into alternative conformations. As a result, the enantioselectivity of the dehalogenases could be predicted much more accurately by using 10 MD simulations of only 10 ps, as these resulted in much more complete conformational sampling than a single 22 ns trajectory. The use of many ultrashort MD simulations was subsequently successfully applied to engineer enantioselective ketoreductases^38^ and epoxide hydrolases^29,30^ by in silico screening thousands of variants.

The most basic protocol resembled these originally used high-throughput simulations. During MD, the *proRR* and *proSS* NACs formed and disappeared at the fs time-scale (Figures 3, S1 and S2). Starting with a single Rosetta-generated E–S complex structure for each LEH variant, 20 independent MD trajectories of 10 ps each were generated (Figure 4A and Table S1). Each trajectory started with independently assigned initial atom velocities. Enantioselectivity predictions were based on counting the occurrence of *proRR* and *proSS* NACs and were made separately for the two active sites. Both active sites gave similar results (Tables S1 and S2). The majority (63%) of the simulations predicted an enantioselectivity opposite to that observed by chiral HPLC analysis (Table S1). At first sight, there appeared to be a bias toward erroneous prediction of (*S,S*)-diol enantioselectivity (Figure 4A) as also indicated by the mean difference between calculated and predicted e.e. values (Table S1), which was +7.7 kJ/mol but would be close to zero in the absence of bias. This bias could, however, be caused by predictions being essentially random, while experimentally there were far more (*R,R*)-diol than (*S,S*)-diol producing variants. The mean absolute error in predicted ΔΔ*G* ^TS^ varied from −18 to +23 kJ/mol, with an absolute average of 10.9 kJ/mol. The results confirmed that NAC-based MD protocols gave poor predictions of the regioselectivity of *cis*-stilbene oxide hydrolysis by LEH variants.

**Figure 4:**
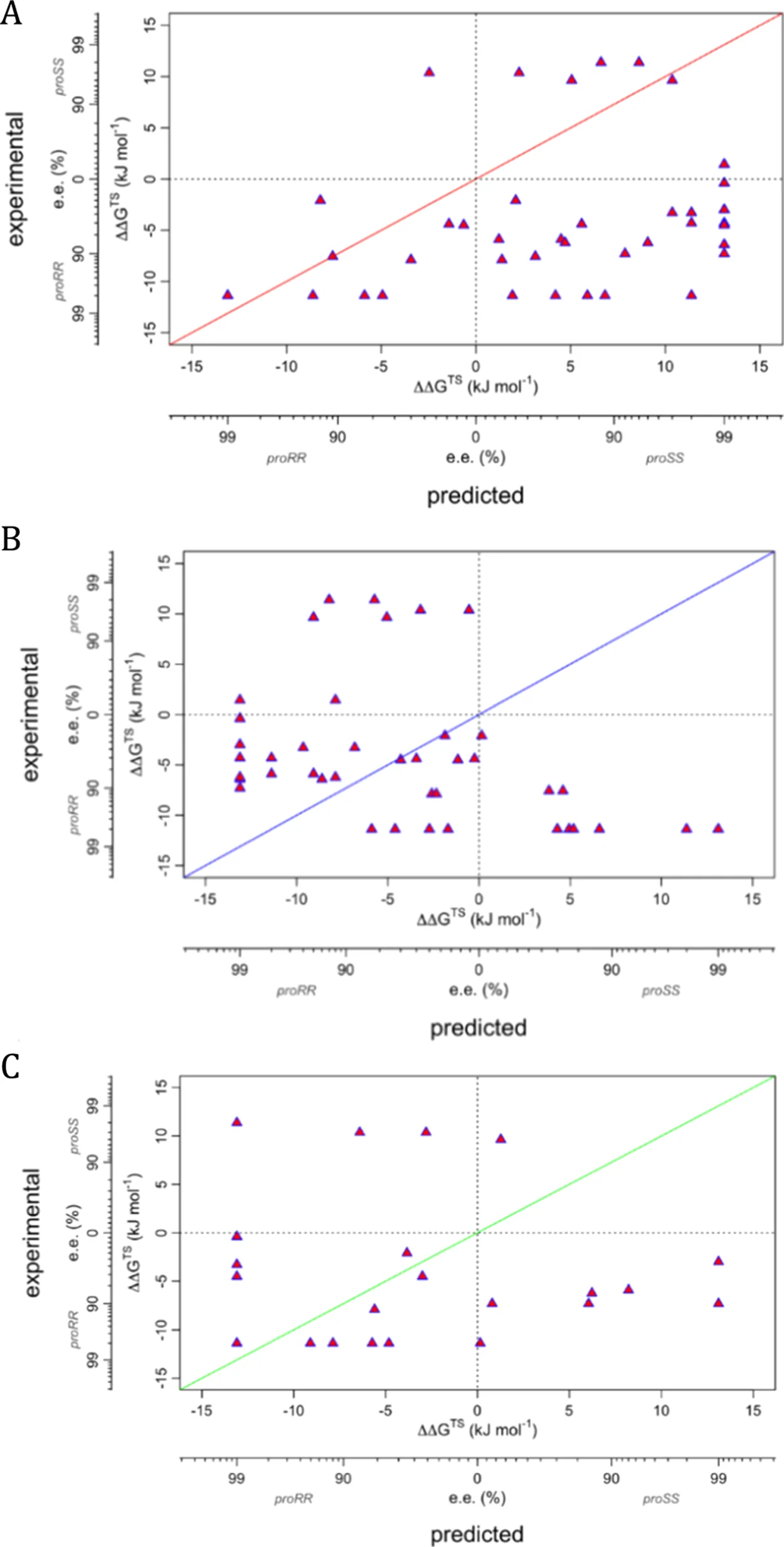
Comparison of computed and experimental regioselectivities. The plot shows differences in transition state free energies (ΔΔ*G* ^TS^) and e.e. values for (*R,R*)-diols vs (*S,S*)-diols determined experimentally by chiral HPLC vs values predicted by MD simulations. In total, 23 different LEH designs are shown. The diagonal lines correspond to perfect predictions. Predicted ΔΔ*G* ^TS^ values are plotted separately for subunit A and B. (A) ΔΔ*G* ^TS^ values predicted using ultrashort MD simulations with a single initial Rosetta-generated E–S complex for each variant. (B) Averaged ΔΔ*G* ^TS^ values predicted through ultrashort MD simulations using 200 initial Rosetta E–S complexes for each variant. (C) Averaged ΔΔ*G* ^TS^ values predicted through 22 ns MD simulations using one initial Rosetta E–S complex for each variant. This panel contains less data since many variants produced NACs neither for *proRR* or *proSS* attack after the 2 ns equilibration period.

To examine if the high-throughput MD protocol could be improved by increasing conformational diversity of starting structures, multiple Rosetta-generated E–S complexes were tested as input for MD simulations. In total 200 E–S complexes per variant were tested. Short MD simulations were subsequently run for a total of 40 ns for each of the 23 catalytically active variants (Figure 4B). However, with multiple starting structures, the prediction accuracy barely improved. Only for a small majority (60%) of the designs the enantiopreference was correctly predicted (Table S1). The mean signed error was −0.9 kJ/mol, indicating there was no systematic bias for *proRR* or *proSS* designs (Table S1). However, none of the five variants that experimentally were (*S,S*)-selective were correctly predicted (Figure 4B). With this more extensive protocol, the mean absolute error in predicted ΔΔ*G* ^TS^ was not significantly improved (9.7 kJ/mol). Thus, also with improved conformational sampling using 200 instead of 1 starting structure per variant, the prediction accuracy was poor for *cis*-stilbene oxide.

We also examined whether the enantioselectivity predictions could be improved by using longer simulation times, which would facilitate escape from energetically unfavorable conformations and improve conformational sampling by allowing drift further away from the starting structures. The longer simulations encompassed 5 trajectories of 20 ns each for a total of 100 ns per variant. However, the predicted ΔΔ*G* ^TS^ values still were in poor agreement with the experimental results (Figure 4C and Table S2). For half of the active sites (52%) no predictions were possible because no NACs were observed at all after the 2 ns equilibration time (Table S2). For the remaining predictions, the accuracy was not significantly improved (mean absolute error 9.2 kJ/mol). Thus, using 2000-fold longer MD simulations did not improve NAC-based prediction of the selectivity of *cis*-stilbene oxide hydrolysis.

Additionally, for the four most important variants, the same that were also studied with DFT (see below), Boltz-2 models were created to investigate whether the Rosetta generated starting structures were reliable. Boltz-2 was selected because it predicts protein–ligand structures using machine learning, making it orthogonal to the original Rosetta method which uses molecular mechanics. The resulting 5 × 20 ns MD simulations gave outcomes that were only correct for the 60A variant and indistinguishable from those with Rosetta starting structures of the same variants (Table S2). The starting structure is arguably less likely to be critical in this specific case as the substrate is small and hydrophobic and fully buried in a hydrophobic cavity; like with the dehalogenases, where 5 ps MD simulations could relax the structure sufficiently,^37^ there are hardly any hydrogen bonds to the substrate or other strong directional interactions to hinder substrate rearrangement.

To evaluate whether simulating as long as possible could make a difference, the MD trajectories for these four variants were further extended to 5 × 500 ns, of which the first 200 ns was used for equilibration (Table S3). This gave the same results as the 5 × 20 ns trajectories. At the end of this 500 ns, the dynamics of exchange between *proRR* and *proSS* NACs were observed to be similar to those in the first 10 ps (Figures 3 and S2). With other enzymes, far more dramatic conformational changes have been observed within this time scale, like for example a 7 Å structural rearrangement of the active site of LovD.^59^ Thus, also 50,000-fold longer MD simulations did not suggest that the poor enantioselectivity predictions were due to there being insufficient time to relax the structure.

The NAC definitions for scoring reactive conformations along MD simulations include the orientation of the water oxygen relative to the atoms of the epoxide group, as mainly determined by *d*_1_ and θ_1A_. Most other NAC criteria, i.e. *d*_2_–*d*_6_ and θ_2_–θ_6_, are the same for *proRR* and *proSS* attack (Figure 2). If the NAC approach were to give better predictions of enantiopreference by adopting its criteria, the prime candidates for modification are the settings of *d*_1_ and θ_1A_ which are expected to determine enantioselectivity. We therefore examined the nucleophilic attack angles (θ_1A_) and the distances of the nucleophile (*d*_1_) occurring in the longer MD simulations (22 ns, Figures S3–S5). Specifically, LEH-P and 60A were analyzed because these variants gave the highest catalytic activity, suggesting that catalytically competent orientations during MD simulation occur frequently. Experimentally, both LEH-P and 60A produce mainly the (*R,R*)-diol.

Subunit A of the LEH-P template gave the highest number of *proSS* NACs, while for subunit B *proRR* and *proSS* NACs occurred in similar numbers. Inspection of a density plot of the attack angles versus distance showed no clear difference between *proRR* and *proSS* NACs that would lead to a different ratio if NAC criteria were made more relaxed, more stringent, or were shifted. The MD simulations reproduced the (*R,R*) enantiopreference of 60A for subunit B (Figure S4), but for subunit A the selectivity was underestimated (Table S2). Again, inspection of the density plot of θ_1A_ versus *d*_1_ (Figure S4) did not suggest a simple way to fix these poor predictions by an empirical adaptation of the NAC criteria.

### Quantum Chemical Modeling

Because predicting the enantioselectivity by a NAC approach did not work, we decided to model the reactions using the more accurate methods provided by DFT calculations. In case of enantioselectivity predictions, cancellation of systematic errors between the very similar transition state structures leads to higher accuracy on their energy difference, as previously demonstrated for various enzymes including LEH.^14,51^ Large cluster models were constructed for LEH-P and three variants with high enantioselectivities, namely 41B, 52A, and 60A (see Materials and Methods section above).

In agreement with previous findings for wild-type LEH with other substrates,^13,14^ the DFT results showed that both the LEH-P and the variants follow a concerted reaction mechanism. This involves the simultaneous deprotonation of the water by Asp132, nucleophilic attack of the water oxygen on the carbon atom, and protonation of the epoxide oxygen by Asp101.

Gratifyingly, the calculated trends in the stereopreference of LEH-P and all three redesigned variants were consistent with the experimental results (Tables 2 and S4). More specifically, for LEH-P and 60A, the calculated energy of the TS leading to the (*R*,*R*)-diol was lower than that leading to the (*S*,*S*)-diol, by 10.5 and 2.9 kJ/mol, respectively. In contrast, for 52A and 41B, the energy of the TS favoring the (*S*,*S*)-diol was found to be lower, by 5.4 and 13.9 kJ/mol, respectively. For these variants, the MD simulations gave an incorrectly predicted enantiopreference, like they did for many mutants. The DFT calculations thus yielded much more accurate results compared to the MD simulations.

**Table 2:** Calculated Energy Barriers for TS­(*S*,*S*) and TS­(*R*,*R*) for *cis*-Stilbene Oxide Hydrolysis by LEH Variants^a^

	preference	Δ*E* ^TS^(*S,S*) (kJ/mol)	Δ*E* ^TS^(*R,R*) (kJ/mol)	ΔΔ*E* ^TS^_calc_ ^b^ (kJ/mol)	ΔΔ*G* ^TS^_exp_ (kJ/mol)	e.e._exp_ ^c^ (%)
LEH-P	(*R,R*)-diol	62.5	52.0	+10.5	+7.1	90
mutant 60A	(*R,R*)-diol	63.5	60.6	+2.9	>+11.3	>99
mutant 52A	(*S,S*)-diol	77.1	82.5	–5.4	–10.0	–97
mutant 41B	(*S,S*)-diol	80.4	94.3	–13.9	–9.2	–95

a The ΔΔ*G* ^TS^_exp_ values were calculated from the e.e. values determined by chiral HPLC.

b ΔΔ*E* ^TS^_calc_ = Δ*E* ^TS^(*S,S*) – Δ*E* ^TS^(*R,R*).

c A negative value indicates a preference for producing the (*S,S*)-diol.

To identify the factors controlling the stereopreference of LEH-P and its three variants with *cis*-stilbene oxide, it is essential to analyze the transition state geometries obtained from the DFT calculations. For LEH-P, the optimized structures of TSs show that the phenyl ring of the substrate that is positioned at the back of the active site (Figure 5) is surrounded by a number of nonpolar residues (Met78, Val83, Leu103, Leu114, Phe134, and Phe139). Among these, the bulky side chains of Leu114 and Leu103 introduce unfavorable steric clashes with this phenyl ring, disfavoring the TS leading to the (*S*,*S*)-diol. In contrast, this steric effect is absent in the TS leading to the (*R*,*R*)-diol, as there is more space in the binding pocket for the phenyl ring at the front. This explanation for (*R,R*)-diol preference applies also to the 60A variant (see geometries in the Supporting Information, Figure S7). Both LEH-P and 60A indeed were experimentally observed to exhibit regioselectivity favoring the (*R*,*R*)-diol as the main product.

**Figure 5:**
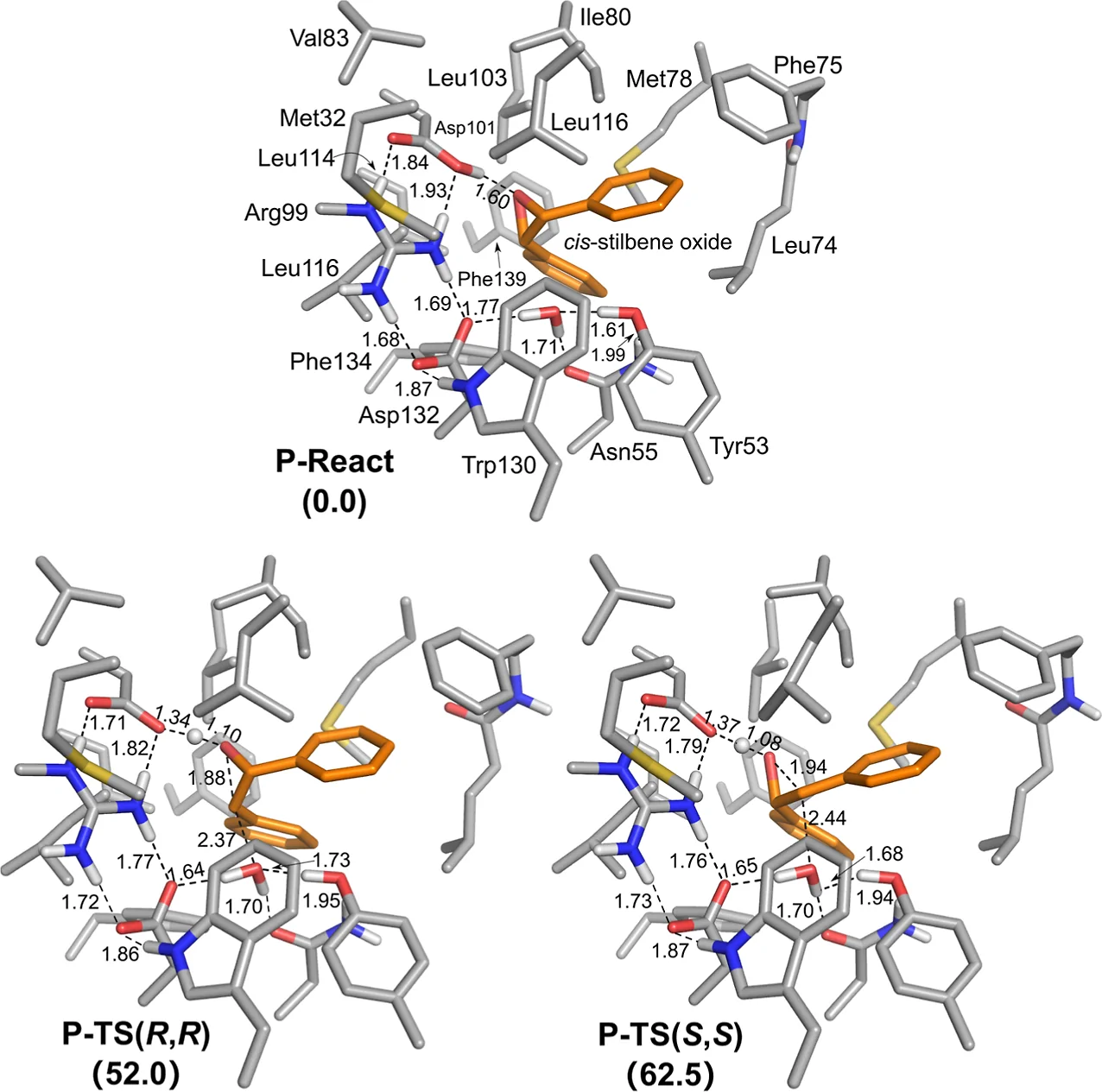
Optimized structures of LEH-P with *cis*-stilbene oxide. Shown are the optimized structures of the enzyme–substrate complex (P-React) and the transition states leading to the formation of (*R*,*R*)-diol and (*S*,*S*)-diol, labeled as P-TS­(*R*,*R*) and P-TS­(*S*,*S*), respectively. The substrate is depicted in orange in all models. For clarity, only polar hydrogens are displayed in the figures. The fixed atoms during the geometry optimization are shown in Figure S6 in the Supporting Information. The energies relative to P-React are given in kJ/mol.

A similar analysis of the TSs of the 41B and 52A variants that favor the formation of the (S,*S*)-diol shows that the side chain of Met35 in both variants introduces steric hindrance to the substrate’s phenyl ring at the front, making the TS leading to the formation of the (*R*,*R*)-diol less favorable compared to the (*S*,*S*)-diol (Figures S8 and S9 in Supporting Information). This effect thus results in a reversed product stereopreference compared to LEH-P and 60A.

Monitoring geometric parameters of the transition states can give further information about the structural origin of selectivity of the different variants (Table 3, Figures 5 and S3–S5). For all the considered variants except LEH-P, the *proRR* transition state appears to be more advanced along the reaction coordinate than the *proSS* transition state. This is evidenced by the distance of the carbon–oxygen bond that is formed during the reaction. For the *proRR* TSs this distance is relatively short at 2.35–2.37 Å, while for the *proSS* TSs it is relatively long at 2.65–2.81 Å, indicating a more substrate-like transition state (Table 3).

**Table 3:** Geometric Parameters of Optimized Transition States of Quantum Chemical Models^a^

		transition state structures of							
		LEH-P		41B		52A		60A	
	NAC criterion	*proRR*	*proSS*	*proRR*	*proSS*	*proRR*	*proSS*	*proRR*	*proSS*
*d*_1_(Å)	<3.22	2.37	2.44	2.35	2.81^f^	2.36	2.65^f^	2.37	2.71^f^
θ_1A_(°)	145.5 ± 17.5	146.1	141.9	146.2	133.6	144.6	144.7	146.8	125.2
θ_1B_(°)	99.5 ± 17.5	98.1	95.3	100.1	89.0	97.6	99.4	103.8	88.5
*d*_2_(Å)	<3.50	1.10	1.08	1.06	1.04	1.06	1.05	1.07	1.05
θ_2_(°)	>120	169.0	170.8	170.1	168.8	171.5	169.9	172.7	173.3
*d*_3_(Å)	<3.5	1.64	1.65	1.59	1.67	1.60	1.65	1.70	1.69
θ_3_(°)	>120	164.9	165.3	169.0	159.8	170.6	165.5	168.6	167.5
*d*_4_(Å)	<3.50	1.73	1.68	1.84	1.67	1.98	1.73	1.71	1.65
θ_4_(°)	>120	153.2	155.7	150.9	157.3	144.3	154.7	155.3	156.5
*d*_5_(Å)	<3.50	1.70	1.70	1.75	1.76	1.74	1.74	1.65	1.67
θ_5_(°)	>120	170.0	167.8	162.7	167.6	165.0	170.4	173.2	172.7
*d*_6_(Å)	<3.50	1.95	1.94	1.93	1.93	1.96	1.93	1.91	1.90
θ_6_(°)	>120	158.4	161.3	159.0	163.5	157.0	162.9	160.9	167.6
distance attacked carbon–epoxide oxygen (Å)^b^	NA	1.88	1.94	1.90	1.96	1.89	1.97	1.90	2.08
dihedral between epoxide and proximal phenyl ring (°)^c^	NA	50.8	16.5	77.3	11.2	78.0	13.7	44.5	15.2
distance water oxygen–nearest proximal phenyl atom (Å)^d^	NA	2.83	2.93	2.33^g^	3.60	2.37^g^	2.94	3.30	3.44
distance water oxygen–nearest distal phenyl atom (Å)^e^	NA	3.09	2.64	3.45	2.30^g^	2.63	2.38^g^	3.37	2.62

a NAC criteria are given for comparison.

b Distance between the epoxide oxygen and the carbon atom that is attacked by the water.

c The dihedral through the two carbon atoms of the epoxide ring, the carbon atom next to the attacked carbon atom of the oxirane group, and the neighboring carbon atom in the same phenyl ring that gave a dihedral closest to zero (the other neighboring phenyl carbon atom would give a value which would be fully equivalent but would differ by 180°, making comparisons harder). This dihedral should be optimal when close to 0° or 180°.

d Proximal to the attacked carbon atom of the oxirane ring. This nearest ring atom was always hydrogen.

e Distal to the same carbon atom. The nearest ring atom was always a hydrogen.

f Indicating a relatively early transition state.

g Within van der Waals overlap.

While the phenyl rings can activate the substrate by stereoelectronic effects, they can also slow down the reaction by introducing steric hindrance to formation of the transition state.^60^ Almost all modeled transition states display steric hindrance between the attacking oxygen and an ortho hydrogen of the distal or proximal phenyl rings, with distances down to 2.30 Å (Table 3), which cannot be detected in the ground-state MD simulations. The shortest distances are clearly within the van der Waals contact distance (1.52 + 1.2 Å, for oxygen and hydrogen radii, respectively) and are observed for the transition states of 41B and 52A.

For the transition state of this nucleophilic attack a partial sp^2^ hybridization of the reacting carbon is required. The transient sp^2^-orbitals can be stabilized by overlap with the π-orbitals of the proximal phenyl ring. This stabilization is optimal if that phenyl ring is coplanar with the carbon atoms of the oxirane.^60^ In line with this notion, for 52A and 41B the dihedral angle that describes the coplanarity is much closer to zero for the favored *proSS* transition state (13.7° and 11.2°, Table 3) than for the disfavored *proRR* attack (78.0° and 77.3°). On the other hand, for variants 60A and LEH-P, *proRR* attack was dominant even though the dihedrals (44.5° and 50.8°, respectively) are less favorable than for *proSS* attack (15.2° and 16.5°), but with smaller differences in angle than with 52A and 41B. This indicates that stereoelectronic effects of the orientation of the proximal phenyl group alone cannot explain enantioselectivity in all variants.

Another important point is the angle of nucleophilic attack, which can reveal the presence of strain in the transition state.^14,61^ For six of the eight considered transition states, the attack angle is in a narrow range of 144–147° (Table 3) which appears to be optimal.^62,63^ Only for 60A, one of the two variants where the alignment of the π-orbitals was not in agreement with the observed enantioselectivity, the nucleophilic attack angle θ_1A_ is indeed suggesting strain in the disfavored *proSS* transition state, being 20° lower. For 41B, which favors *proSS* attack, the angle for this *proSS* attack is also somewhat smaller (12° below optimal) with a smaller difference to the value for the disfavored *proRR* attack.

Finally, the DFT calculations show that the NAC criteria used for predicting enantioselectivities from MD trajectories were appropriate, as they agree with angles and distances optimized for the TSs (Table 3). The NAC distance criteria were based on van der Waals overlap between reacting atoms, rather than on the transition state. Criteria with shorter distances are not feasible, as during the MD simulations the ground state is sampled and distances will stay much longer than in the transition state. During MD, the nucleophilic attack distance *d*_1_ was never below 2.8 Å (Figures S3 and S4), a much longer distance than in most of the transition states (2.35–2.71 Å), considering that only for the 41B variant the distance *d*_1_ was 2.81 (Table 3). Angles involving hydrogen bonds were not part of NAC criteria since broad values were set (>120°), to avoid scoring conformations that miss a catalytically relevant H-bond as catalytically competent. The remaining θ_1A_ and θ_1B_ angles (Figure 2) are almost always in agreement with the NAC definitions. Furthermore, the orientation of the proximal phenyl ring appears unsuitable for inclusion in the NAC definitions because of the large range values in the transition states as found by DFT calculations; the range is 3-fold wider than that of the attack angle θ_1A_ (Table 3). These considerations do suggest that the NAC definitions could not be adapted to provide better predictions of enantioselectivity with the stilbene oxide substrate.

## Conclusions

In this work we have investigated the use of MD simulations and DFT calculations to explain the product enantiopreference of LEH variants in *cis*-stilbene oxide hydrolysis and to understand why this selectivity is poorly predicted by the frequently used MD-based NAC approach. Initially, experimental data for 23 LEH variants plus wild type were compared to the e.e. values predicted by the occurrence of *proRR* and *proSS* NACs in MD trajectories. Despite extensive conformational sampling, both through longer trajectories and by increasing the number of independent runs, the NAC approach gave inaccurate predictions of the product enantioselectivity of *cis*-stilbene oxide conversion. The DFT results gave good agreement with experimental data, and the obtained transition state structures provided hints as to why NAC scoring failed to predict the chirality of the diol formed by *cis*-stilbene oxide hydrolysis.

Previous computational studies on LEH showed that the relative proximity of the electrophilic carbon atoms to the attacking water oxygen and possibly the alignment of the reacting atoms were sufficient to predict activity and regioselectivity.^29,30,37^ Accordingly, conversion of cyclopentene oxide by LEH variants could be modeled successfully with a NAC approach.^29,30^ A DFT study showed that the positive effect of mutations on regioselectivity was due to small shifts of the position of this substrate in the active site cavity, causing the reacting atoms to be better positioned for nucleophilic attack by water.^13,14^ For such relatively small substrates with energy barriers that depend on simple geometric variables, the NAC approach can thus correctly predict enantioselectivity. The same was found with dehalogenases, for which the nucleophilic attack angles were the main criterion to distinguish preferred from disfavored substrate enantiomers.^37^ NAC approaches solely based on distances and angles between atoms involved in catalysis were also used to explain the enantioselectivity and specificity of other enzymes^52,53,64^ and to obtain alcohol dehydrogenases with improved enantioselectivity.^38,65^

The nucleophilic ring opening of *cis*-stilbene oxide studied here differs in two important aspects from the reactions of small molecules that are well modeled by the NAC approach. First, benzylic carbons are more susceptible to S_N_2 reactions due to stereoelectronic effects. Second, the phenyl rings can cause steric hindrance, depending on the torsion angle between the ring system relative to the C–C bond of the epoxide ring. Electronic activation was proposed to contribute to the S_N_2 reactivity of styrene oxide with an α/β-class epoxide hydrolase and the observed enantioselectivity could be partially explained by varying degrees of alignment of the epoxide and phenyl rings.^60^ Such an effect may hold for variant 52A and 41B of limonene epoxide hydrolase as well, where the best alignment for stereoelectronic activation is indeed observed for the lowest barrier transition state. However, alignment of the phenyl ring appears to be less influential for enantioselectivity of *cis*-stilbene oxide hydrolysis by LEH-P and 60A, since the relevant dihedral was worse for the transition state giving the experimentally observed product. With those two variants, however, the differences in dihedral between favored and disfavored transition state were much less than with variants 52A and 41B (Table 3). We therefore conclude that alignment for stereoelectronic activation was not (always) the most important factor determining regioselectivity in LEH, unlike what was found for *trans*-stilbene oxide hydrolysis by an α/β-class epoxide hydrolase.^66^

The results reported here for *cis*-stilbene oxide show that another dominant factor influencing both activity and regioselectivity is steric hindrance to the approaching water nucleophile due to the orientation of a phenyl ring, which cannot be observed during ground-state modeling. The calculated barriers for LEH-P, for which the transition states did not show signs of such steric hindrance (last rows in Table 3), were 52.0 and 62.5 kJ/mol for *proSS* and *proRR* transition states, respectively, which is significantly lower than for the transition states where steric hindrance was apparent. This steric hindrance by the phenyl groups was apparent in five of the eight modeled transition states, with very short distances between the attacking oxygen atom and hydrogen atoms of the phenyl ring (Table 3). It is due to the restricted space in the active site, which prevents the phenyl ring from adopting a rotamer that creates space for the nucleophilic water to attack as the E–S complex moves from the ground state to the transition state. This steric hindrance caused by the phenyl rings can explain the large differences in Δ*G* ^TS^ between some of the reactions, with the most facile reaction at 52.0 kJ/mol and the most disfavored transition state being more than 40 kJ/mol higher, at 94.3 kJ/mol (Table 2). The DFT cluster model of the active site could treat this complicated mix of stereoelectronic effects and steric hindrance and accurately predict the barriers and thereby enantioselectivity in cases where the NAC approach failed.

The NAC approach is computationally far cheaper than DFT and allows screening of thousands of enzyme variants in a short time,^30,37^ raising questions regarding which aspects of the transition state geometries and the energy barriers are not accounted for by MD simulations and if there is a possible solution. In a previous paper, improper description of the effect of π–π interactions was proposed to cause these problems,^29^ but π–π interactions do not significantly contribute to interaction energies of aromatic systems with single rings, like between stilbene oxide and surrounding phenyl and tyrosine rings.^67–69^ Insufficient conformational sampling during the MD simulations was also considered to cause inaccurate predictions,^37^ but since the use of 200 starting structures instead of a single one and 50,000× longer MD simulations did not improve NAC-based predictions we dismissed this explanation.

In general, DFT outperforms the NAC approach because DFT also models the transition state of the reaction and not only the ground state. While a NAC approach can be effective in giving computationally affordable and practically useful predictions in certain cases, it can fail for several reasons. For example, if the rate-limiting or enantioselectivity-determining step of the catalytic cycle is multiple reaction steps away from the Michaelis-complex, the NAC approach becomes irrelevant and modeling the relative stability of a nearby covalent intermediate is more attractive.^70,71^ In the one-step reaction studied here though, the specific failure of the NAC approach for stilbene oxide appears to be caused by stereoelectronic effects and steric hindrance, both dependent on the orientation of the phenyl rings of the substrate during the transition state. It seems unlikely that a NAC approach could be adapted to successfully model stereoelectronic activation and the relevant steric clashes with the phenyl rings only appear in the transition state; they are not apparent in the ground state that is studied during MD simulations (distance *d*_1_ in Figures S3 and S4). Thus, the current results indicate that the presence of bulky or stereoelectronically activating groups near the reacting atoms should be seen as an additional warning sign that a NAC approach can fail.

Another advantage of the DFT calculations over MD simulations is that the former can predict the energy barriers of reactions, which the NAC approach cannot.^72–77^ Although very powerful, DFT can, however, only be applied to a small number of variants due to its large computational cost. In contrast, high-throughput MD simulations encompassing multiple independent short runs can be used for in silico screening thousands of variants.^37^ Furthermore, MD simulations have been found to allow computationally designed (or docked) enzyme–substrate complexes to leave local minimum conformations, resulting in more realistic predictions for reactivity,^78,79^ though the same can be done by DFT. The results of the present work suggest that in silico screening of better enzyme variants by MD simulations with the NAC approach should ideally be followed by a screening of the top-ranked variants using a QM method like DFT, which can accurately calculate barriers even when complicated steric hindrance and stereoelectronic activation are involved.

## Supplementary Material



## Data Availability

All stationary points are available in the Supporting Information.

## Notes

Tables of NAC frequencies and predictions for each variant, the applied energetic corrections, visualizations of NACs, plots of their occurrence and geometric distributions, and visualizations and Cartesian coordinates of the stationary points (PDF)
