# Supplementary material for: Unraveling a Case Where the NAC Approach Fails—MD and QM Modeling of Regioselective cis-Stilbene Oxide Hydrolysis by Engineered Epoxide Hydrolases
Source: J Chem Inf Model. 2026 Sep 22;66(19):12877–90. doi: 10.1021/acs.jcim.6c02029 (PMC13663864; doi:10.1021/acs.jcim.6c02029)
Supplement: Supplementary file 1 [file ci6c02029_si_001.pdf]

Supplementary Materials belonging to

Unraveling a case where the NAC approach fails - MD and QM modeling of regioselective *cis*-stilbene oxide hydrolysis by engineered epoxide hydrolases

Elvira Bombino[1], Hesam Arabnejad[1], Baoyan Liu[3], Peter A. Jekel[1], Xiang Sheng[3], Dick B. Janssen[1], Fahmi Himo[2*], Hein J. Wijma[1*]

[1] Biotransformation and Biocatalysis, Groningen Biomolecular Sciences and Biotechnology Institute, University of Groningen, Nijenborgh 4, 9747AG, Groningen, the Netherlands.
[2] Department of Chemistry, Arrhenius Laboratory, Stockholm University, 10691, Stockholm, Sweden.
[3] State Key Laboratory of Engineering Biology for Low-Carbon Manufacturing, Tianjin Institute of Industrial Biotechnology, Chinese Academy of Sciences, Tianjin 300308, P.R. China.
* Corresponding authors: fahmi.himo@su.se, h.j.wijma@rug.nl

1. Supplementary Tables

Table S1. Short (20 × 10 ps trajectories) MD simulations and calculated ΔΔG^{TS} values for stereoselective *cis*-stilbene oxide hydrolysis by redesigned LEHs. ΔΔG values were calculated according to Equation 1, with pro*RR* and pro*SS* NAC frequencies obtained for subunits A and B of 23 variants by HTMI-MD simulations. For each variant, simulations were started both with a single E-S complex and with 200 independent Rosetta-generated E-S complexes. Experimental ΔΔG values were obtained by chiral HPLC using Equation 2. The data are plotted in Figure 1 in the main text. Negative values represent (*R,R*)-diol selectivity, positive values indicate (*S,S*) selectivity.

Variant	experimental ΔΔG^{TS} (kJ/mol)	predicted ΔΔG^{TS} (subunit A) 1 E-S complex (kJ/mol)	predicted ΔΔG^{TS} subunit B 1 E-S complex (kJ/mol)	predicted ΔΔG^{TS} (subunit A) 200 E-S complexes (kJ/mol)	predicted ΔΔG^{TS} (subunit B) 200 E-S complexes (kJ/mol)
proRR-60A	-11.38	-13.11	-13.11	11.38	13.11
proRR-51A	-7.57	-7.6	3.14	4.60	3.84
proSS-52A	10.37	-2.46	2.30	-0.55	-3.21
proRR-38A	-11.38	5.90	4.20	-1.70	-4.60
proRR-48A	-11.38	-5.90	-4.94	4.94	4.30
proRR-63B	-2.10	2.10	-8.22	-1.87	0.15
proRR-49A	-11.38	6.82	11.38	-2.72	-5.90
proRR-30A	-6.41	13.11	13.11	-13.11	-8.61
proRR-61B	-7.90	-3.43	1.37	-2.59	-2.34
proRR-47B	-11.38	-8.6	1.92	6.61	5.20
proRR-25A	-4.30	11.38	13.11	-11.38	-13.11
proRR-3A	-0.40	13.11	13.11	-13.11	-13.11
proRR-50A	-3.29	11.38	10.37	-6.82	-9.64
proRR-24A	-3.00	13.11	13.11	-13.11	-13.11
proSS-59A	9.64	5.10	10.37	-5.06	-9.08
proSS-46C	11.38	6.61	8.61	-5.73	-8.22
proSS-65B	1.43	13.11	13.11	-7.90	-13.11
proRR-31A	-4.40	-1.43	5.60	-0.25	-3.43
proRR-4C	-7.30	7.90	13.11	-13.11	-13.11
proRR-32A	-5.90	1.22	4.50	-11.38	-9.077
proRR-26A	-4.50	-0.7	13.11	-1.16	-4.30
proRR-43A	-6.22	9.10	4.71	7.90	-13.11
proSS-41B	9.10	-8.6	-4.94	-6.61	-13.11

Numbers printed in red indicate predictions deviating from experimental enantiopreference. The predictions for the single complexes are based on 5,000 geometry measurements, that were evenly spaced (every 20 fs using on the fly measurements, see materials and methods) during the production phase of the 20 trajectories. For the 200 complexes, 1,000,000 geometry measurements were used per active site per variant.

Table S2. Longer (5 × 22 ns) MD simulations results for stilbene oxide. Predicted ΔΔG values are obtained from NAC occurrences, experimental values from enantiomeric excess measured by chiral HPLC.

variant	subunit A		subunit B		subunit A	subunit B	experimental ΔΔG (kJ/mole)
	**proSS NACs (%)**	**proRR NACs (%)**	**proSS NACs (%)**	**proRR NACs (%)**	**predicted ΔΔG (kJ/mole)**	**predicted ΔΔG (kJ/mole)**	
proRR-LEH-P	5.68	0.50	3.24	2.33	6.05	0.80	-7.30
proRR-LEH-P [A]	6.74	0.48	2.382	0.11	6.55	7.62	-7.30
proRR-60A	3.00	21.20	0.10	30.17	-4.82	-13.11	-11.38
proRR-60A[A]	0.828	20.898	0.18	12.312	-8.00	-10.47	-11.38
proRR-51A	0.00	0.00	0.00	0.00	--	--	-7.57
proSS-52A	0.02	0.07	0.23	3.17	-2.80	-6.41	10.37
proSS-52A[A]	0.316	6.9	0.382	9.752	-7.64	-8.03	10.37
proRR-38A	0.00	0.00	0.01	0.23	--	-7.87	-11.38
proRR-48A	0.00	0.00	1.28	23.63	--	-9.10	-11.38
proRR-63B	0.00	0.00	1.38	6.57	--	-3.84	-2.10
proRR-49A	0.00	0.00	0.05	0.51	--	-5.73	-11.38
proRR-30A	0.00	0.00	0.00	0.00	--	--	-6.41
proRR-61B	0.00	0.00	0.25	2.41	--	-5.60	-7.90
proRR-47B	0.00	0.01	1.73	1.63	-13.11	0.15	-11.38
proRR-25A	0.00	0.00	0.00	0.00	--	--	-4.30
proRR-3A	0.00	0.00	0.00	44.00	--	-13.11	-0.40
proRR-50A	0.00	0.04	0.00	0.01	-13.11	-13.11	-3.29
proRR-24A	0.01	0.00	0.02	0.00	--	13.11	-3.00
proSS-59A	0.00	0.00	2.00	1.20	--	1.27	9.64
proSS-46C	0.00	0.00	0.00	1.13	--	-13.11	11.38
proSS-65B	0.00	0.00	0.00	0.00	--	--	1.43
proRR-31A	0.00	0.00	0.00	0.00		--	-4.40
proRR-4C	0.00	0.00	28.61	0.02	--	13.11	-7.30
proRR-32A	0.00	0.00	0.27	0.01	--	8.22	-5.90
proRR-26A	0.00	0.01	0.30	1.00	-13.11	-3.00	-4.50
proRR-43A	0.00	0.00	1.44	0.13	--	6.23	-6.22
proSS-41B	0.00	0.00	0.30	1.00	--	-3.00	9.10
proSS-41B [A]	1.36	6.47	0.388	6.36	-3.86	-6.93	9.10

Red fonts indicate that the sign of the prediction is incorrect. The NAC percentages and the derived **ΔΔG** predictions are based on 500,000 geometry measurements, except for those complexes obtained using Boltz-2 derived structures. For those, the frequency of sampling was set less intensive (because the simulations were subsequently continued for in total 500 ns, see materials and methods), resulting in 10,000 geometry measurements per active site during the first 22 ns (of which the very first 2 ns was used for equilibration).

[A] Using a Boltz-2 generated starting structure instead of a Rosetta generated starting structure.

Table S3. Results with the longest (5 × 500 ns) MD simulations. Predicted ΔΔG values are obtained from NAC occurrences, experimental values from enantiomeric excess measured by chiral HPLC. For each simulated variants the fractions of NACs are indicated per trajectory. The values shown immediately behind the name of the variant are the average from 5 trajectories.

variant	subunit A		subunit B		subunit A	subunit B	experimental ΔΔG (kJ/mole)
	***proSS* NACs (%)**	***proRR* NACs (%)**	***proSS* NACs (%)**	***proRR* NACs (%)**	**predicted ΔΔG (kJ/mole)**	**predicted ΔΔG (kJ/mole)**	
**proRR-LEH-P**	1.039	0.057	3.376	0.186	7.19	7.18	-7.30
**traj. 1**	5.193	0.285	0.870	0.007			
**traj. 2**	0.000	0.000	0.000	0.000			
**traj. 3**	0.001	0.000	0.000	0.000			
**traj. 4**	0.000	0.000	6.083	0.063			
**traj. 5**	0.000	0.000	9.925	0.862			
**proRR-60A**	0.019	5.584	1.161	6.763	-13.1	-4.37	-11.38
**traj. 1**	0.096	27.903	0.000	0.000			
**traj. 2**	0.000	0.000	0.000	0.000			
**traj. 3**	0.000	0.015	0.000	0.000			
**traj. 4**	0.000	0.001	4.738	13.631			
**traj. 5**	0.000	0.000	1.069	20.182			
**proSS-52A**	**0.212**	**0.624**	**0.294**	**0.888**	-2.67	-2.74	10.37
**traj. 1**	0.000	0.000	0.000	0.013			
**traj. 2**	1.058	3.109	0.041	1.441			
**traj. 3**	0.000	0.000	0.063	1.119			
**traj. 4**	0.000	0.004	0.373	0.671			
**traj. 5**	0.000	0.009	0.995	1.197			
**proSS-41B**	0.023	1.143	0.411	1.441	-9.68	-3.11	9.10
**traj. 1**	0.000	0.000	0.343	1.131			
**traj. 2**	0.006	0.707	0.000	0.000			
**traj. 3**	0.029	4.663	0.249	0.983			
**traj. 4**	0.001	0.001	0.008	1.067			
**traj. 5**	0.079	0.345	0.079	1.127			

Red fonts indicate that the sign of the prediction is incorrect. Each **ΔΔG** prediction is based on 750,000 on-the-fly geometry measurements (150,000 for the production phase of each trajectory, the first 200 ns was used for equilibration).

Table S4. Absolute energies, energy corrections, and relative energies of the enzyme-substrate complexes and the transition states leading to formation of (*R*,*R*)- and (*S*,*S*)-enantiomers.
MB: 6-31G(d,p) basis set
LB: 6-311+G(2d,2p) basis set

	$E_{MB\text{-}Gas}$ (au)	$E_{LB\text{-}Gas}$ (au)	$E_{MB\text{-}SMD}$ (au)	E_{ZPE} (au)	E_{Total} (au)	ΔE_{Total}[a] (kJ/mol)
**LEH-P**						
**P-React**	-6110.496669	-6110.489337	-6111.958214	2.848051	-6109.102831	**0.0**
**P-TS(*R*,*R*)**	-6110.477788	-6110.467323	-6111.938819	2.845347	-6109.083008	**52.0**
**P-TS(*S*,*S*)**	-6110.472598	-6110.46275	-6111.934419	2.845528	-6109.079043	**62.5**
**Mutant 60A**						
**60A-React**	-6121.561856	-6121.552295	-6123.096019	3.111733	-6119.974725	**0.0**
**60A-TS(*R*,*R*)**	-6121.538304	-6121.525089	-6123.074485	3.109637	-6119.951632	**60.6**
**60A-TS(*S*,*S*)**	-6121.534461	-6121.524819	-6123.069577	3.109391	-6119.950544	**63.5**
**Mutant 52A**						
**52A-React**	-6619.242119	-6619.233890	-6620.830273	3.166494	-6617.655551	**0.0**
**52A-TS(*R*,*R*)**	-6619.207817	-6619.199638	-6620.796414	3.164101	-6617.624134	**82.5**
**52A-TS(*S*,*S*)**	-6619.207964	-6619.200125	-6620.798345	3.164310	-6617.626197	**77.1**
**Mutant 41B**						
**41B-React**	-6864.991280	-6864.981877	-6866.572591	3.143940	-6863.419248	**0.0**
**41B-TS(*R*,*R*)**	-6864.955188	-6864.945650	-6866.535389	3.142527	-6863.383325	**94.3**
**41B-TS(*S*,*S*)**	-6864.959395	-6864.949857	-6866.540388	3.142213	-6863.388637	**80.4**

[a] $E_{Total} = E_{LB\text{-}Gas} + (E_{MB\text{-}SMD} - E_{MB\text{-}Gas}) + E_{ZPE}$

2. Supplementary Figures

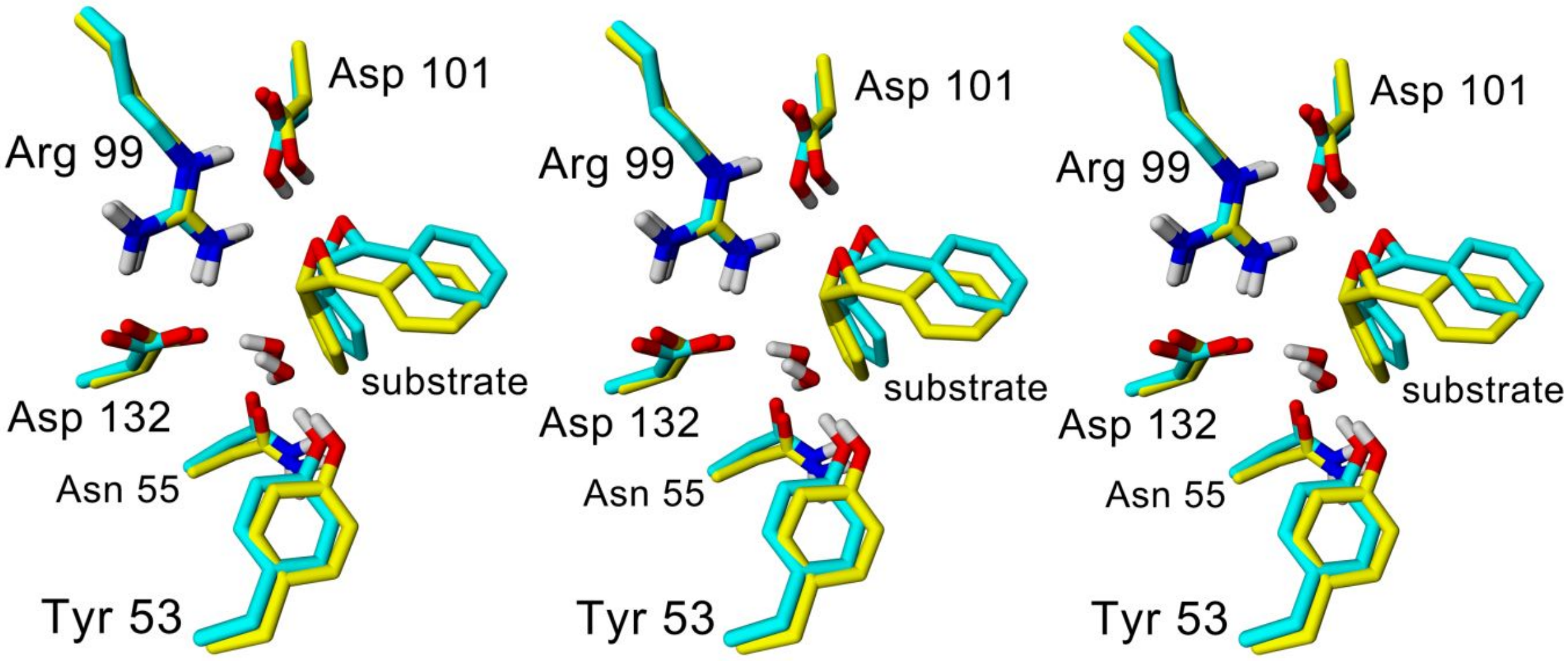

Figure S1. Snapshots of different NACs. These additional snapshots were taken at moments that a NAC was detected (see Figure 3 of the main manuscript, at points indicated with an asterisk sign). The proSS NAC (with yellow carbon atoms) was taken 1.2 ps after the proRR NAC (with cyan carbon atoms). These snapshots were superimposed on the C_α atoms of subunit A of the enzyme. The left side of the picture present cross-eyed stereo while the right side allows for parallel stereo viewing.

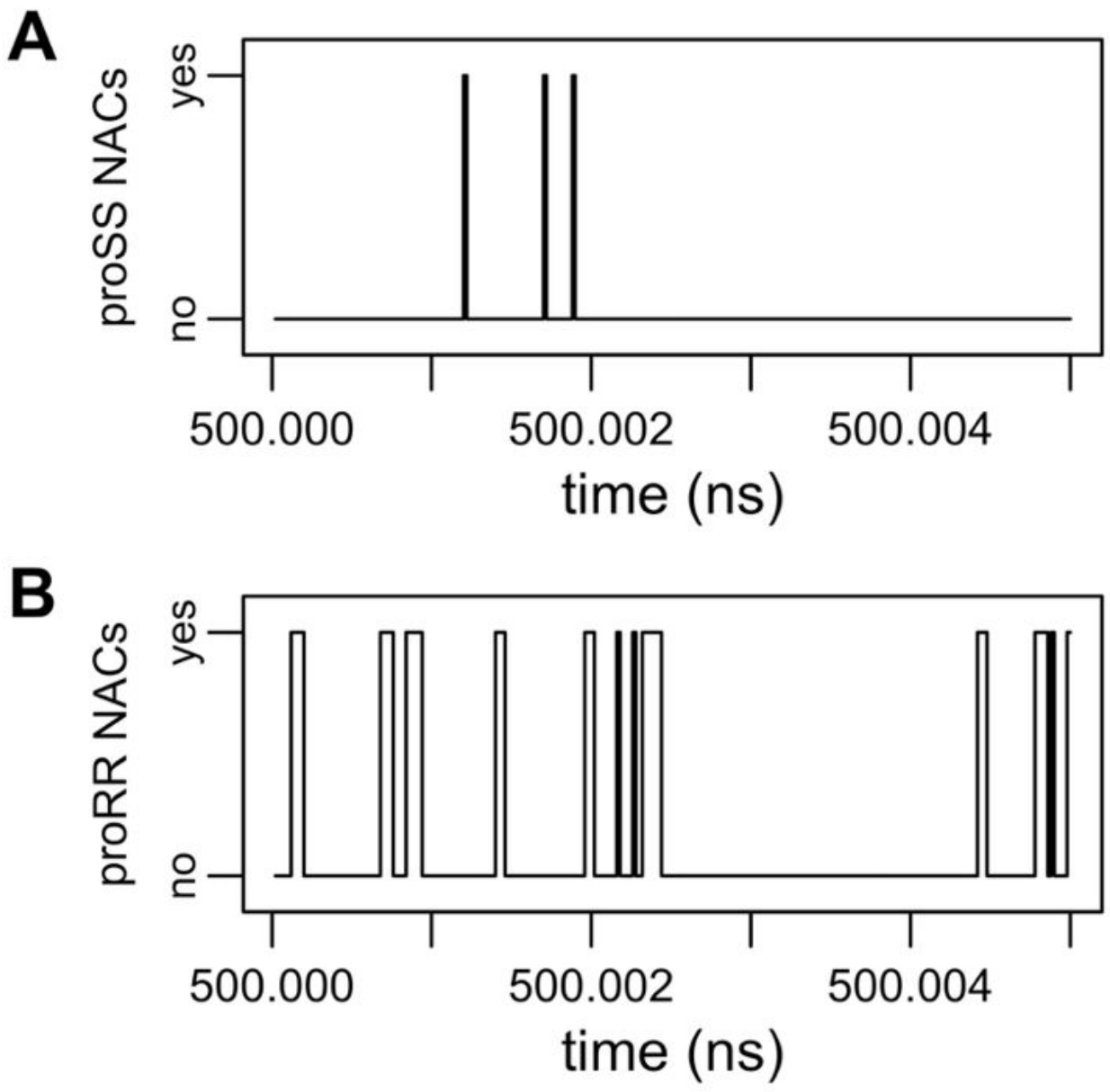

Figure S2. Dynamic exchange of NACs after 500 ns of MD simulation. The time scale (5 ps wide) and conformational evaluation rate (once every 20 fs) was chosen to be identical to Figure 3. To measure with this dense sampling rate (for the first 500 ns less frequent evaluations were used to speed up the calculations), the snapshot at 500 ns (the file of which included the atom velocities at that moment) was used as the starting point for an additional 5 ps. The results are for 60A's subunit B of trajectory 4, which was chosen because it displayed relatively high NACs (see Table S3).

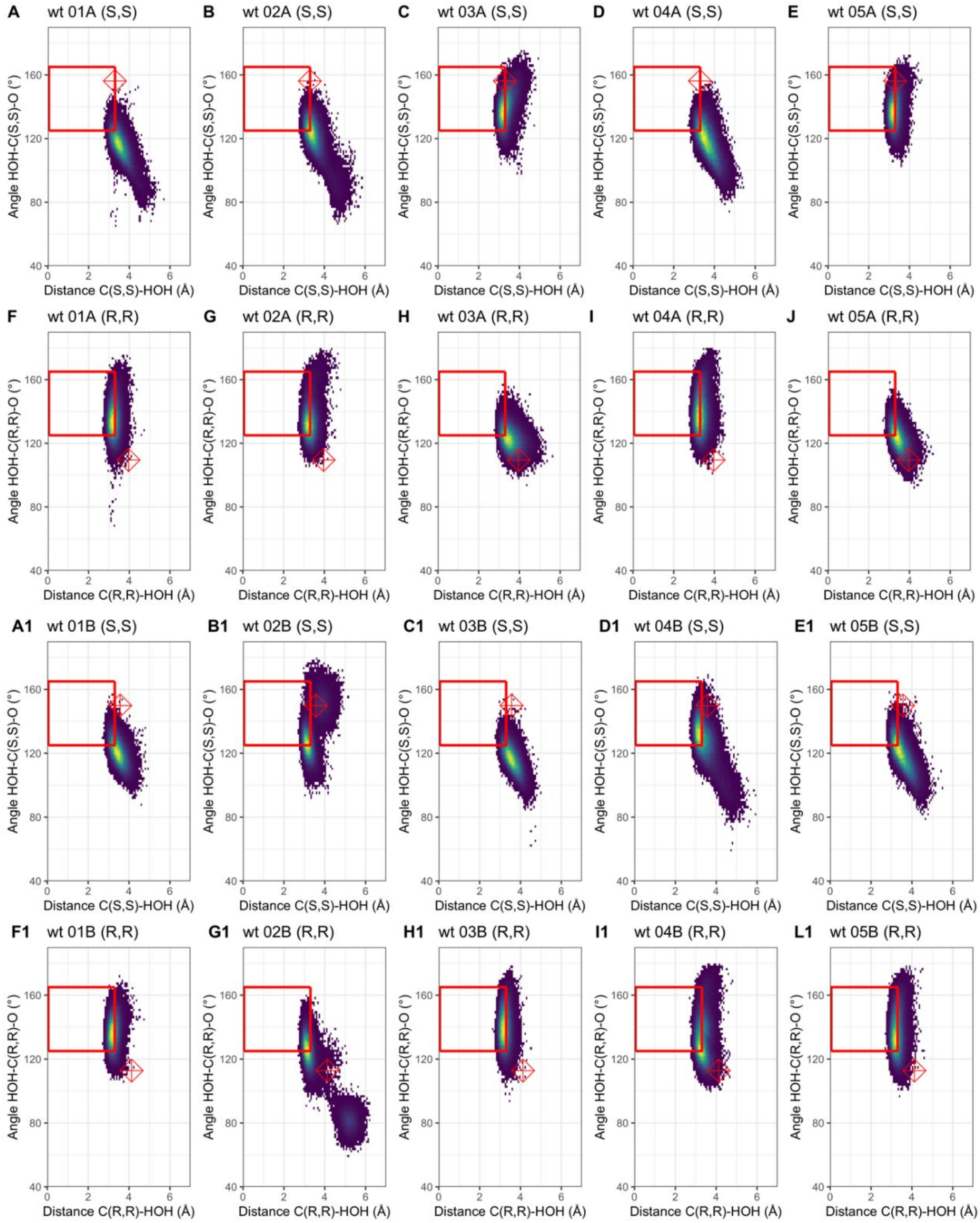

Figure S3. Dynamics of the water molecule in LEH-P subunit A and subunit B. The panels display the position of the water molecule in the LEH-P active site along 22 ns MD simulations, 5 seeds. The initial position of the water molecule after Rosetta redesign is shown with a red marker for the pro*SS* (subunit A A-E; subunit B A1-E1) and pro*RR* carbon atoms (subunit A F-J; subunit B F1-J1). The red boxes indicate fulfillment of the NAC criterion for nucleophilic attack. The most populated area shows the water molecule partially in a NAC conformation along the simulations. The yellow and green colors represent the most populated region. The violet dots show the less populated zone. Experimentally, LEH-P produces (*R,R*)-diol.

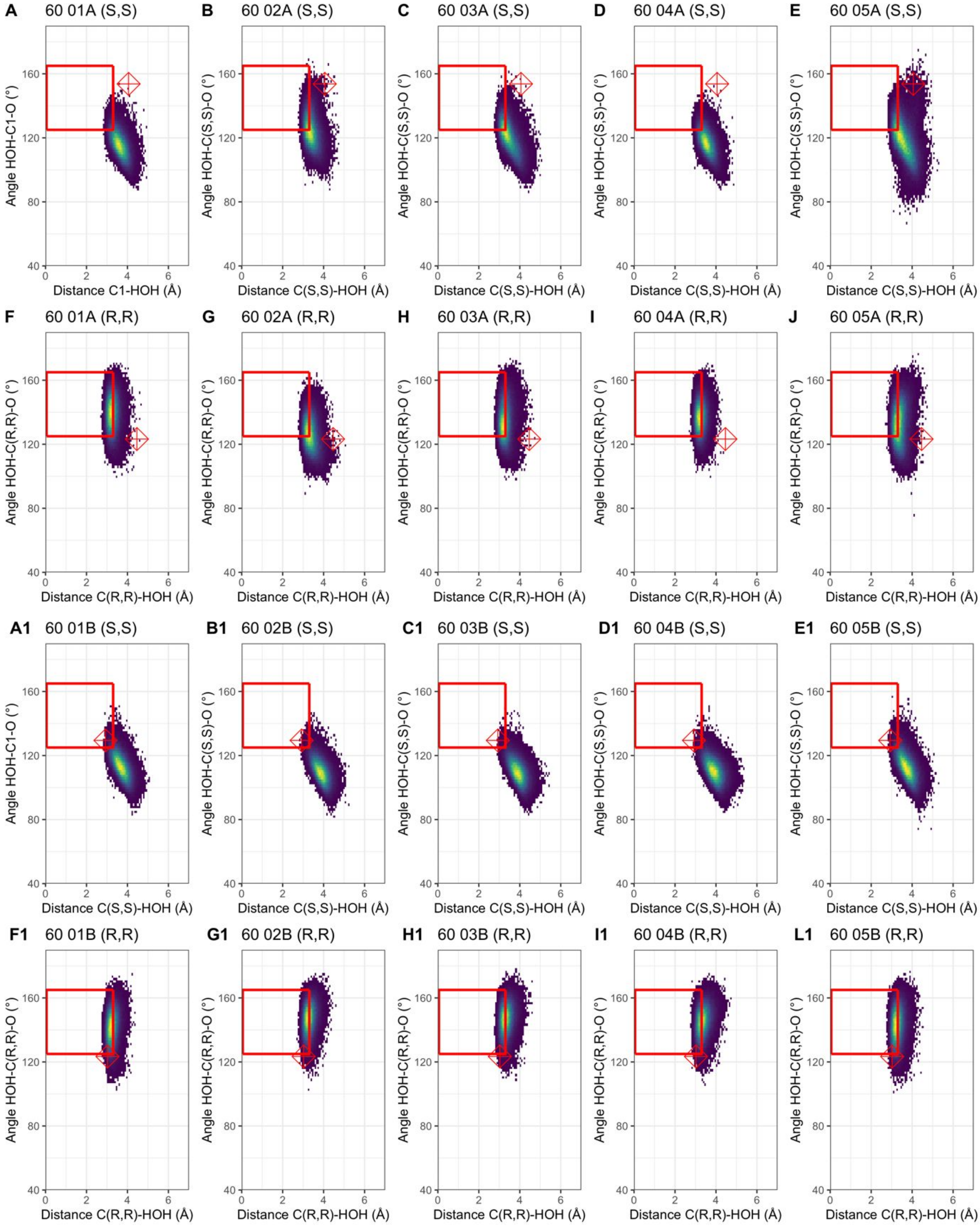

Figure S4. Water molecule in LEH design 60A subunit A and subunit B. The position of the nucleophilic water molecule in the 60A LEH active site is shown along 22 ns MD simulations, 5 seeds. The initial position of the water molecule after Rosetta redesign is shown in red in pro*SS* (subunit A A-E; subunit B A1-E1) and pro*RR* positions (subunit A F-J; subunit B F1-J1). The red boxes indicate fulfillment of the NAC criteria for nucleophilic attack. Experimentally the 60A variant is selective for production of (*R,R*)-diol.

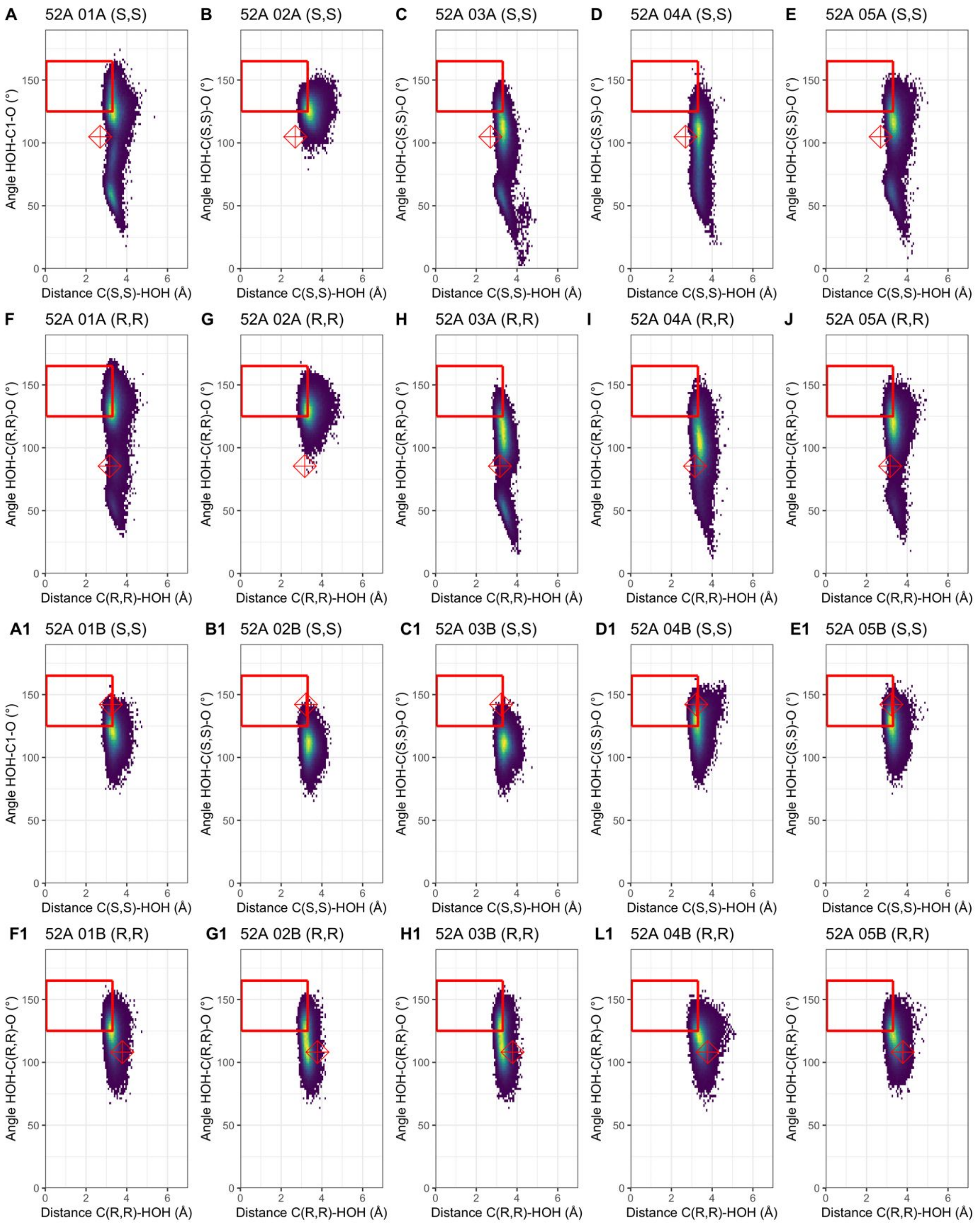

Figure S5. Water molecule in 52A subunit A and subunit B. The 52A variant produces mainly the (*S,S*)-diol. The position of the water molecule in the active site is shown along 22 ns MD simulations, 5 seeds. The initial position of the water after Rosetta redesign is shown in red for pro*SS* NACs (subunit A A-E; subunit B A1-E1) and pro*RR* NACs (subunit A F-J; subunit B F1-J1). The red boxes indicate fulfillment of the NAC criteria for nucleophilic attack.

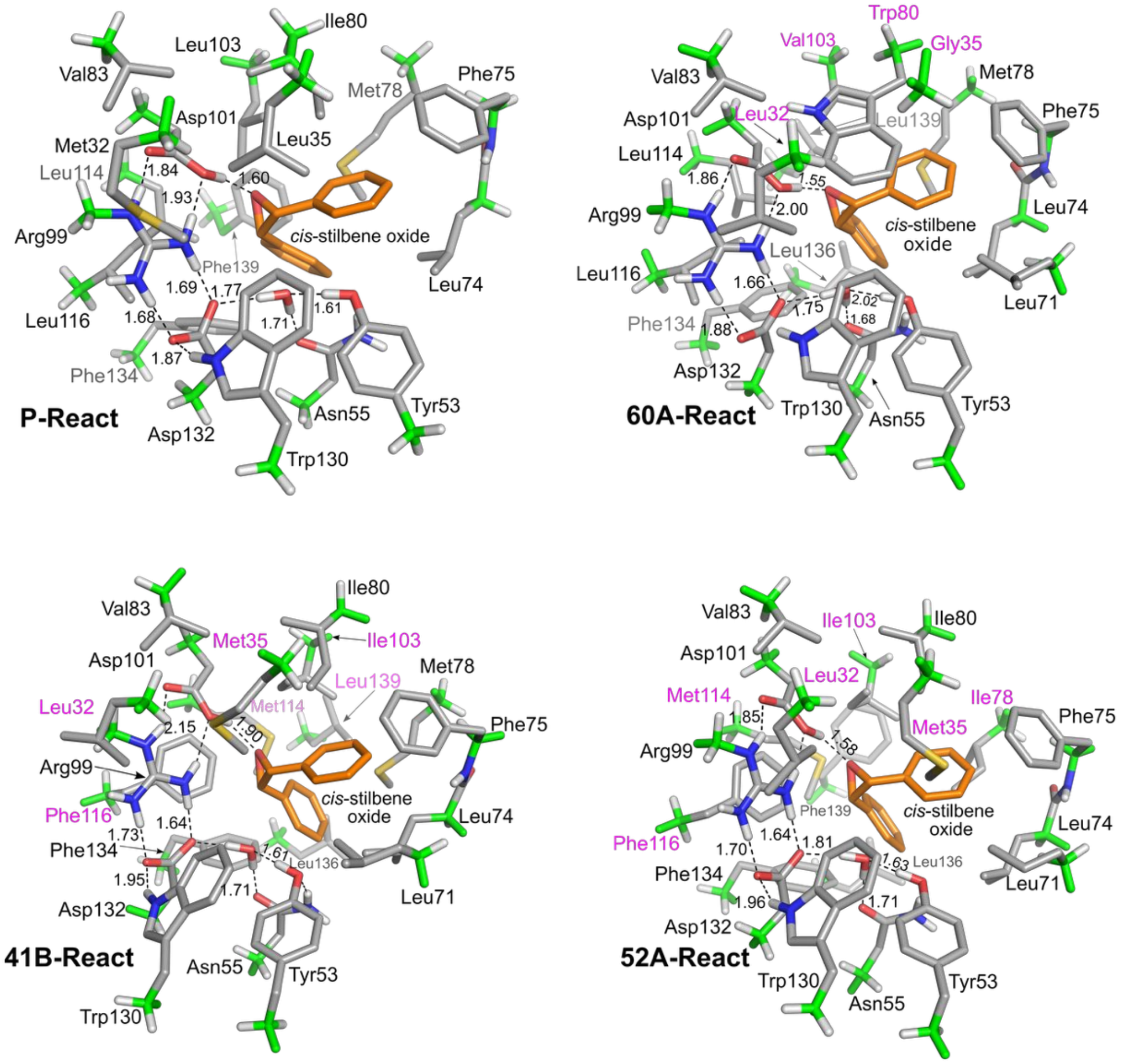

Figure S6. Optimized structures of LEH-P and three redesigned mutants, 60A, 41B and 52A, with *cis*-stilbene oxide. The substrate is depicted in orange in all models. For clarity, only selected hydrogens are displayed. The atoms that were fixed during geometry optimization are highlighted in green.

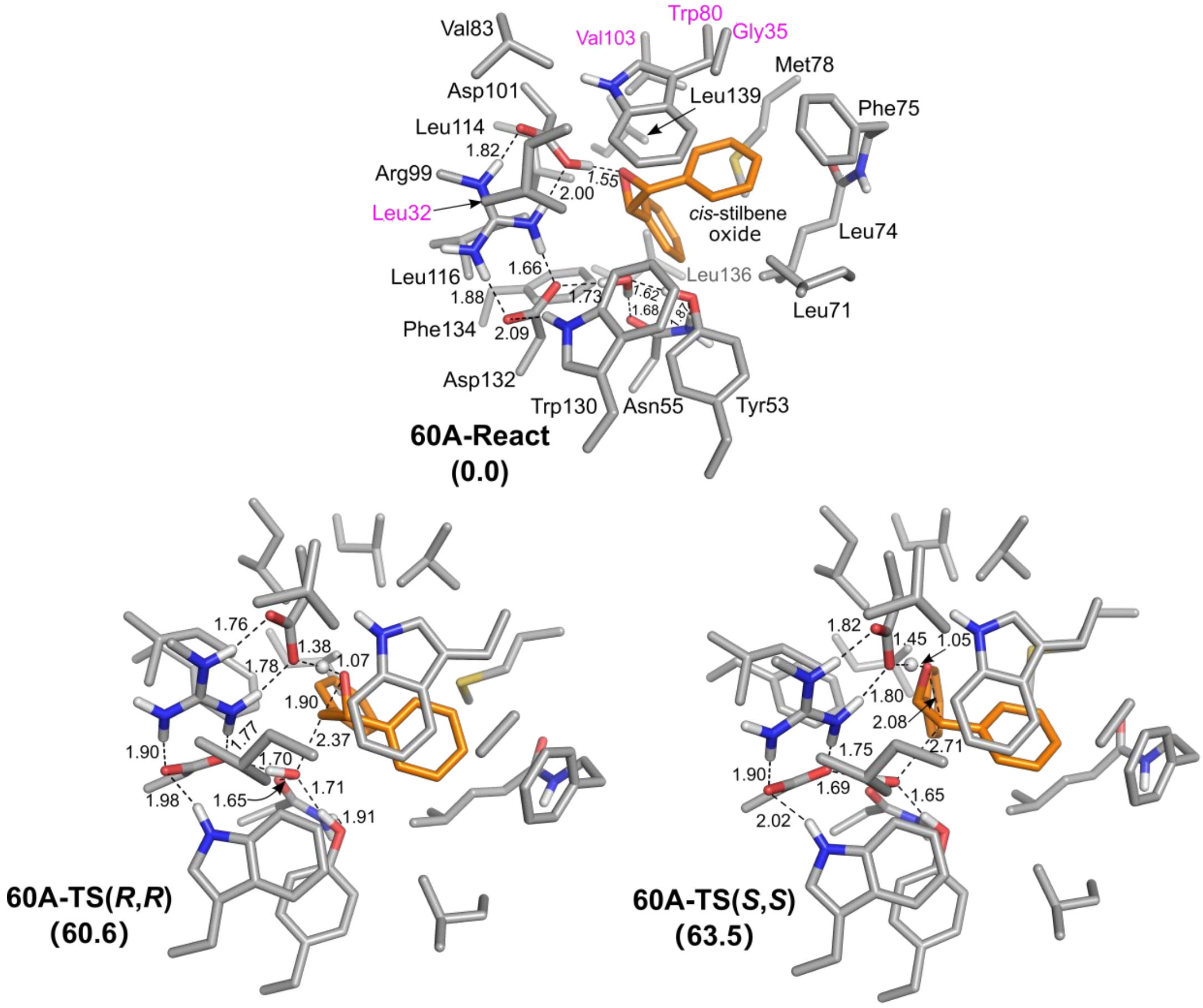

Figure S7. Optimized structures of 60A with *cis*-stilbene oxide. Shown are the optimized structures of the enzyme-substrate complex (60A-React) and the transition states leading to the formation of (*R*,*R*)-diol and (*S*,*S*)-diol, labeled as 60A-TS(*R*,*R*) and 60A-TS(*S*,*S*), respectively. The substrate is depicted in orange in all models. For clarity, only polar hydrogens are displayed. The fixed atoms during the geometry optimization are shown in Figure S4. The energies relative to **60A-React** are given in kJ/mol.

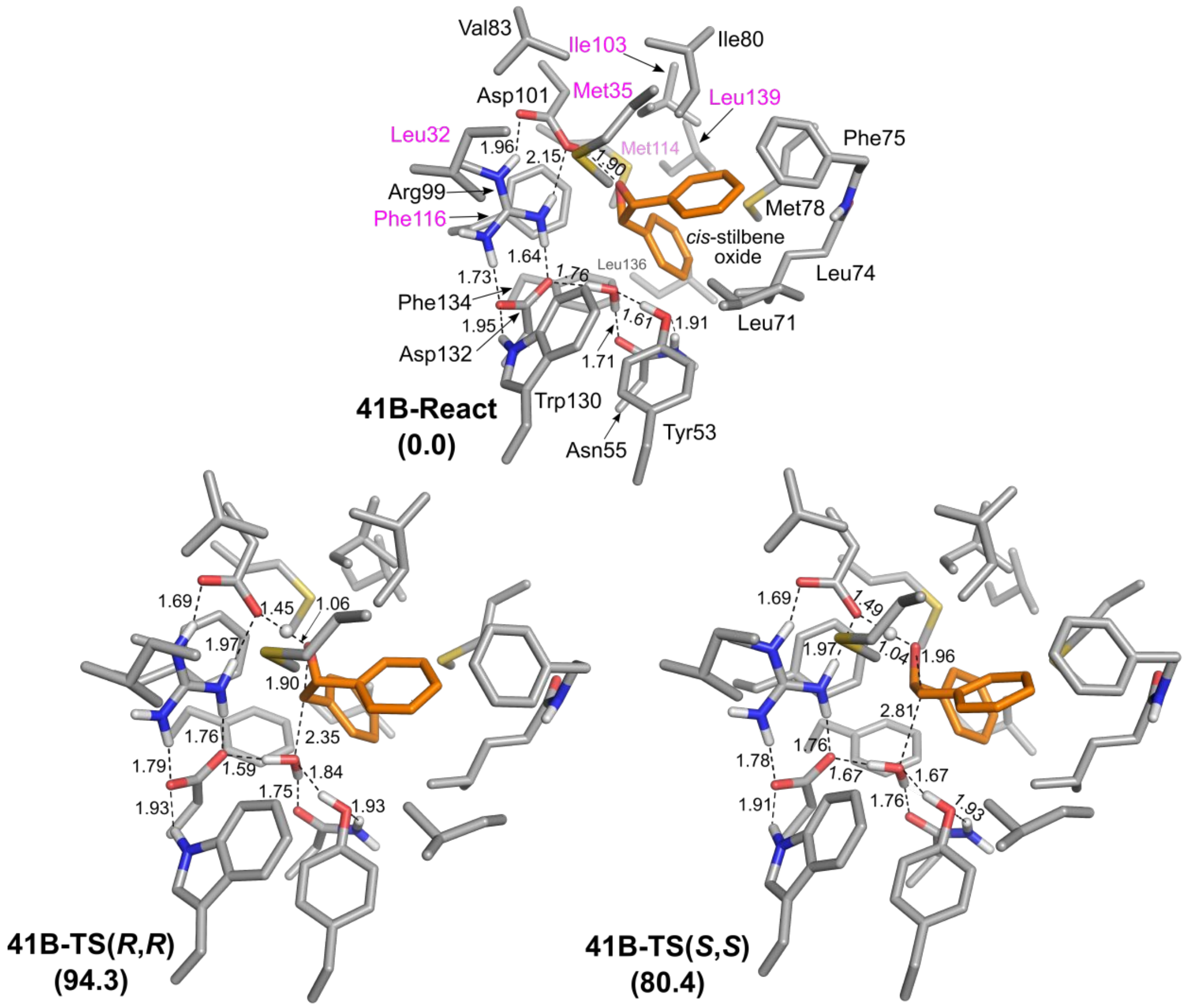

Figure S8. Optimized structures of 41B with *cis*-stilbene oxide. Shown are the optimized structures of the enzyme-substrate complex (41B-React) and the transition states leading to the formation of (*R*,*R*)-diol and (*S*,*S*)-diol, labeled as 41B-TS(*R*,*R*) and 41B-TS(*S*,*S*), respectively. The substrate is depicted in orange in all models. For clarity, only polar hydrogens are displayed. The fixed atoms during the geometry optimization are shown in Figure S4. The energies relative to **41B-React** are given in kJ/mol.

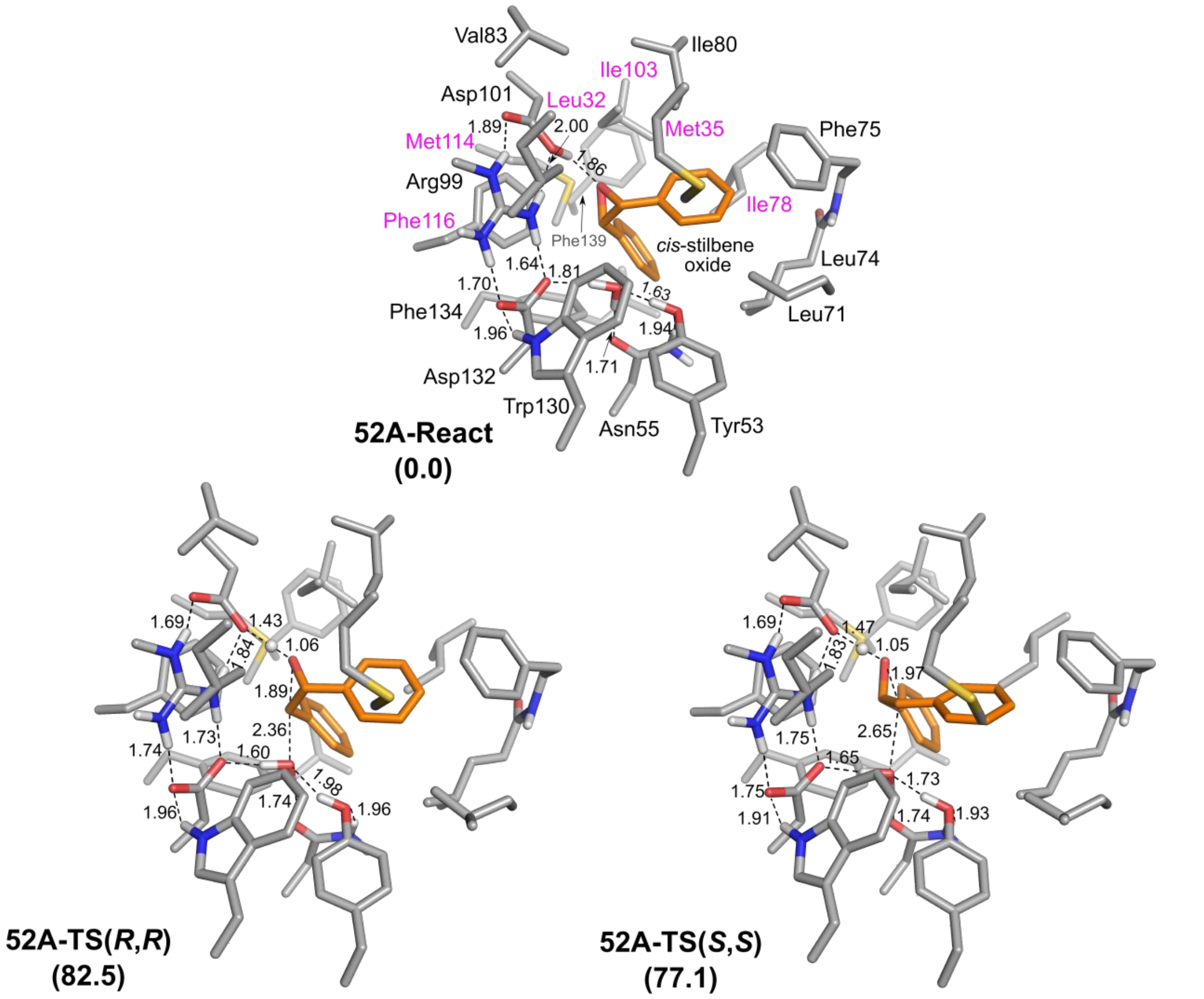

Figure S9. Optimized structures of 52A with *cis*-stilbene oxide. Shown are the optimized structures of the enzyme-substrate complex (52A-React) and the transition states leading to the formation of (*R*,*R*)-diol and (*S*,*S*)-diol, labeled as 52A-TS(*R*,*R*) and 52A-TS(*S*,*S*), respectively. The substrate is depicted in orange in all models. For clarity, only polar hydrogens are displayed. The fixed atoms during the geometry optimization are shown in Figure S4. The energies relative to **52A-React** are given in kJ/mol.

3. Cartesian Coordinates of Optimized DFT Geometries

LEH-P-React

C	8.03400	1.90300	5.32100
C	8.35200	0.42300	5.52900
C	8.73400	-0.31700	4.24200
S	7.34900	-0.55200	3.04800
C	7.62000	0.79800	1.84700
H	8.92099	2.46700	5.01300
H	7.27100	2.02300	4.54600
H	9.18099	0.31800	6.24200
H	7.49000	-0.08200	5.97900
H	9.55200	0.18900	3.71800
H	9.08300	-1.32600	4.48299
H	8.63100	0.75000	1.43500
H	6.90400	0.64800	1.03600
H	7.44500	1.77900	2.29400
C	3.70900	5.10600	5.05800
C	3.61100	3.64000	4.60100
C	4.39300	3.25200	3.33300
C	4.27100	1.74100	3.08800
C	3.91600	4.02600	2.09600
H	3.66500	5.80100	4.21400
H	3.93000	2.99000	5.42600
H	2.55900	3.39500	4.42000
H	4.62899	5.30900	5.61300
H	5.45600	3.49000	3.49400
H	3.21600	1.44600	3.00900
H	4.76499	1.44300	2.16000
H	4.71800	1.15799	3.89900
H	2.86800	3.78900	1.87200
H	3.99100	5.11000	2.23000
H	4.51400	3.76200	1.22000
C	4.15700	4.16399	-7.86900
C	3.45400	3.76500	-6.59600
C	3.77200	2.57799	-5.92300
C	2.43299	4.56300	-6.06600
C	3.08500	2.18000	-4.77799
C	1.73800	4.18499	-4.92199
C	2.05799	2.98600	-4.27700
O	1.29800	2.62600	-3.20400
H	3.61300	3.81700	-8.75700
H	4.24899	5.25100	-7.95200
H	4.57300	1.94600	-6.29800
H	5.16300	3.73700	-7.92100
H	2.17600	5.49700	-6.56100
H	3.34200	1.26099	-4.26400
H	0.94800	4.80500	-4.50900
H	1.61400	1.75900	-2.81400
C	0.16700	-1.77100	-7.21300
C	-0.68000	-0.58099	-6.76000
C	-0.17600	0.01400	-5.45600
N	-0.44300	1.32000	-5.23500
O	0.39400	-0.69900	-4.62100
H	-0.23700	-2.20000	-8.13400
H	1.20000	-1.46700	-7.39900
H	-0.72700	0.19400	-7.53400
H	-1.71500	-0.90100	-6.57700
H	-0.02600	1.77900	-4.42500
H	-0.71400	1.91200	-6.00400
H	0.17800	-2.54300	-6.44200
C	-5.36900	5.99700	-2.67900
C	-5.83200	6.58800	-1.33200
O	-6.97700	6.42000	-0.92000
C	-3.90600	5.52800	-2.69700
C	-3.51200	4.67699	-3.91800
C	-1.98500	4.55199	-4.01600
C	-4.15400	3.28600	-3.86200
H	-6.05800	5.17300	-2.87600
H	-3.24300	6.40300	-2.66100
H	-3.69400	4.94500	-1.79200
H	-3.87300	5.19200	-4.82000
H	-1.50900	5.53600	-4.08800
H	-1.56800	4.04200	-3.14100
H	-1.69600	3.98000	-4.90299
H	-3.89200	2.69900	-4.74899
H	-3.79700	2.73600	-2.98400
H	-5.24600	3.33400	-3.81300
N	-4.92100	7.33400	-0.63500
C	-5.22100	7.92900	0.67100
C	-4.11300	8.90600	1.07900
C	-2.76200	8.24100	1.23300
C	-2.49900	7.41600	2.33599
C	-1.75200	8.41600	0.27900
C	-1.26000	6.79900	2.48900
C	-0.50800	7.79600	0.42700
C	-0.25900	6.98800	1.53500
H	-5.33000	7.14300	1.43000
H	-4.05100	9.70800	0.33600
H	-4.40700	9.36900	2.02599
H	-3.27200	7.26700	3.08600
H	-1.93400	9.06400	-0.57599
H	-1.07300	6.16900	3.35200
H	0.26300	7.94900	-0.32200
H	0.70200	6.49700	1.65200
H	-3.95200	7.31200	-0.91600
C	-7.62000	4.22300	1.90900
C	-6.89400	4.62800	3.19700
C	-7.26900	2.80900	1.42000
C	-5.79500	2.66100	1.04500
S	-5.29500	1.00299	0.44700
C	-6.08400	0.98100	-1.19700
H	-8.70200	4.26700	2.08000
H	-7.24600	5.60100	3.55500
H	-7.06300	3.89700	3.99600
H	-5.81200	4.71000	3.04900
H	-7.51400	2.06900	2.19300
H	-7.88800	2.56900	0.54800
H	-5.52100	3.40100	0.28399
H	-5.15299	2.83700	1.91400
H	-7.17100	0.91000	-1.12700
H	-7.40700	4.94900	1.11400
H	-5.80300	1.87000	-1.76600
H	-5.69100	0.10300	-1.71200
C	-2.44400	2.56800	6.12800

C	-1.10100	2.72500	5.46500
C	-1.27899	3.17400	4.00100
C	-0.26100	1.44500	5.56500
C	0.01600	3.53100	3.27500
H	-2.34900	2.24300	7.17100
H	-3.00200	3.51100	6.12700
H	-0.55100	3.52200	5.99000
H	-1.79800	2.38400	3.44400
H	-1.95100	4.04300	3.98200
H	-0.18300	1.10000	6.60200
H	-0.72300	0.64400	4.97800
H	0.75500	1.58900	5.18800
H	0.66700	2.65900	3.15600
H	-0.18900	3.91800	2.27599
H	0.57999	4.29200	3.82600
H	-3.06400	1.82900	5.61000
C	3.16200	-1.70700	8.58900
C	3.91200	-0.83000	7.57600
C	4.72800	-1.69300	6.60400
C	2.93900	0.07800	6.81600
H	3.85200	-2.33800	9.16100
H	2.59200	-1.09900	9.29900
H	4.61100	-0.19000	8.13299
H	4.06300	-2.37200	6.06100
H	5.24900	-1.07600	5.86300
H	5.47500	-2.29300	7.13600
H	2.21800	-0.52100	6.25200
H	2.38000	0.72700	7.49900
H	3.46500	0.71700	6.09900
H	2.45699	-2.37000	8.07300
C	5.55800	-3.60100	3.35900
N	4.56600	-2.73700	2.75400
C	4.44000	-2.57100	1.43000
N	3.41200	-1.86400	0.94500
N	5.33500	-3.07900	0.57999
H	5.45700	-4.63900	3.01700
H	6.57200	-3.24300	3.15200
H	3.80300	-2.40600	3.34700
H	2.68300	-1.58200	1.59100
H	3.19300	-1.93600	-0.07000
H	6.12400	-3.59000	0.93800
H	5.14799	-3.06200	-0.44800
H	5.40900	-3.57600	4.43800
C	0.03100	-3.35000	5.66100
C	-0.11800	-2.38899	4.48299
C	1.21900	-2.00400	3.89400
O	2.27900	-2.37100	4.37800
O	1.23700	-1.22500	2.81100
H	-0.95300	-3.61600	6.05700
H	0.54200	-4.26499	5.35500
H	-0.73300	-2.82000	3.68400
H	-0.62900	-1.46700	4.78300
H	0.61600	-2.89300	6.46200
C	-5.46200	-1.10600	5.42800
C	-5.28200	-1.48000	3.95200
C	-3.80900	-1.60200	3.52200
C	-3.66900	-2.23400	2.13700
C	-3.10200	-0.24400	3.55600
H	-5.03400	-0.12600	5.66400
H	-6.51700	-1.09000	5.71600
H	-5.77600	-2.44400	3.78000
H	-5.78400	-0.75400	3.30000
H	-3.31100	-2.26600	4.24700
H	-4.08900	-1.58500	1.36400
H	-2.61300	-2.38300	1.89300
H	-4.17300	-3.20100	2.07500
H	-2.09500	-0.30600	3.13500
H	-3.65200	0.48800	2.95400
H	-3.02500	0.14499	4.57200
C	-2.88800	-7.06000	3.34500
C	-3.14100	-6.02700	2.24500
C	-3.14600	-6.58700	0.81100
C	-4.26999	-7.60600	0.59400
C	-3.25100	-5.44400	-0.20500
H	-3.68200	-7.81200	3.39100
H	-4.10300	-5.52600	2.42400
H	-2.37600	-5.24200	2.30900
H	-2.18500	-7.09900	0.64900
H	-4.26199	-7.97800	-0.43600
H	-4.18300	-8.47000	1.25899
H	-5.24500	-7.13800	0.77300
H	-2.37800	-4.78899	-0.14499
H	-3.29900	-5.81800	-1.23000
H	-4.14900	-4.84300	-0.02200
C	2.38800	-7.64900	0.29300
C	2.29300	-6.15200	0.60300
C	0.83900	-5.74800	0.91300
C	2.91000	-5.34300	-0.54100
C	0.67900	-4.29800	1.37100
H	1.80000	-7.89700	-0.59900
H	3.42200	-7.95000	0.09900
H	2.88300	-5.95400	1.51200
H	0.21400	-5.91700	0.02800
H	0.44900	-6.41500	1.69400
H	3.97800	-5.55500	-0.64400
H	2.43900	-5.59400	-1.49700
H	2.80300	-4.26900	-0.40100
H	0.97000	-3.59500	0.58700
H	-0.36200	-4.08399	1.63800
H	1.30700	-4.09900	2.24600
C	8.19000	0.77400	-6.40100
C	7.69500	1.67400	-5.26300
C	7.00700	0.91200	-4.16600
C	7.03400	-0.44800	-3.96100
C	6.18300	1.45800	-3.11300
C	5.75500	0.36200	-2.30700
C	5.73200	2.75000	-2.79300
N	6.30900	-0.78400	-2.83400
C	4.90800	0.53200	-1.20800
C	4.87399	2.92000	-1.71300
C	4.46800	1.82100	-0.92900
H	8.94699	0.06500	-6.05100
H	8.53500	2.25400	-4.85400
H	6.99700	2.41900	-5.66900
H	7.52300	-1.22000	-4.53599
H	6.02600	3.60100	-3.40000
H	6.00900	-1.72600	-2.58400
H	4.59400	-0.30800	-0.60200
H	4.50000	3.91100	-1.47300
H	3.79800	1.98100	-0.09000

C	3.72900	-3.97600	-4.63500
C	3.39700	-2.62800	-3.98000
C	3.68200	-2.60900	-2.48100
O	4.79699	-3.06200	-2.08700
O	2.80000	-2.11700	-1.70300
H	3.06600	-4.76199	-4.26000
H	4.02600	-1.84200	-4.42100
H	2.35800	-2.34100	-4.15700
C	-0.76500	-8.81199	-3.62000
C	-1.66300	-7.82000	-2.86400
C	-1.36000	-6.36800	-3.17100
C	-0.19500	-5.76600	-2.67900
C	-2.23500	-5.58700	-3.93400
C	0.08100	-4.41899	-2.91400
C	-1.95900	-4.24099	-4.18499
C	-0.80500	-3.64800	-3.67100
H	-1.01400	-9.84600	-3.35900
H	-2.71300	-8.02600	-3.10000
H	-1.55200	-7.98800	-1.78500
H	0.49600	-6.36300	-2.09300
H	-3.14600	-6.03400	-4.32300
H	0.97200	-3.95800	-2.49900
H	-2.65800	-3.64500	-4.76400
H	-0.59800	-2.59900	-3.84900
O	1.58600	0.21000	-2.38700
H	1.09800	-0.17600	-3.14800
H	2.19200	-0.50800	-2.10700
C	-0.31300	-0.82700	-0.28199
O	-0.58500	-0.56100	1.14399
H	4.75900	-4.26400	-4.41200
H	3.60200	-3.92200	-5.72000
H	0.37300	-1.08200	2.31300
H	-0.88000	-8.69400	-4.70200
H	0.28999	-8.64600	-3.38100
H	8.62500	1.35800	-7.21700
C	-0.39200	1.75700	0.17900
C	0.44400	2.78100	-0.27900
C	-1.73900	2.02500	0.44100
C	-0.06300	4.06300	-0.48000
H	1.48300	2.56800	-0.50700
C	-2.23700	3.31400	0.25600
H	-2.39200	1.23000	0.77600
C	-1.40300	4.33500	-0.20100
H	0.59000	4.84600	-0.85100
H	-3.27900	3.52000	0.47300
H	-1.78900	5.34000	-0.32700
C	-1.49600	-0.89900	-1.18500
C	-1.68800	0.02800	-2.21400
C	-2.38600	-1.96900	-1.04900
C	-2.76000	-0.12100	-3.09300
H	-0.99400	0.84900	-2.32799
C	-3.46700	-2.11000	-1.91300
H	-2.22600	-2.69900	-0.26700
C	-3.65100	-1.18500	-2.94300
H	-2.88600	0.59200	-3.90000
H	-4.14600	-2.94600	-1.79500
H	-4.48100	-1.30100	-3.63500
H	-5.53700	6.74900	-3.46000
H	-6.18600	8.43800	0.60000
H	0.42100	-1.61800	-0.42500
H	2.84300	5.33300	5.69800
H	7.62600	2.36600	6.23200
H	-4.94800	-1.86000	6.04200
H	2.00700	-8.26000	1.11900
H	-1.94200	-7.58800	3.17800
H	-2.83200	-6.58200	4.32900
H	7.34000	0.19800	-6.79400
C	0.18500	0.40000	0.35200
H	1.24700	0.39600	0.59300
C	-8.88400	-7.33100	-0.31900
C	-7.72100	-6.69300	-1.10000
C	-7.37400	-5.28400	-0.66500
C	-7.20100	-4.96100	0.68800
C	-7.20700	-4.26600	-1.61100
C	-6.88100	-3.66200	1.08000
C	-6.88300	-2.96400	-1.22200
C	-6.71900	-2.65500	0.12800
H	-8.64600	-7.43600	0.74400
H	-6.83300	-7.33100	-0.99600
H	-7.96300	-6.68300	-2.16800
H	-7.32500	-5.72900	1.44500
H	-7.33800	-4.49500	-2.66500
H	-6.76300	-3.43200	2.13300
H	-6.76700	-2.19000	-1.97400
H	-6.47200	-1.64500	0.43900
H	-9.10600	-8.32900	-0.70700
H	-9.78900	-6.72100	-0.40000

LEH-P-TS-RR

C	8.04200	1.89800	5.38400
C	8.30400	0.40500	5.56200
C	8.69800	-0.31700	4.26800
S	7.33500	-0.50600	3.04200
C	7.62600	0.87900	1.88800
H	8.95400	2.43800	5.10600
H	7.29700	2.06500	4.59900
H	9.10800	0.25400	6.29400
H	7.41300	-0.07900	5.97300
H	9.53800	0.18100	3.77100
H	9.01900	-1.33700	4.49700
H	8.64600	0.85100	1.49900
H	6.93000	0.74500	1.05700
H	7.43300	1.84600	2.35700
C	3.71500	5.10200	5.14200
C	3.61700	3.64000	4.66500
C	4.39300	3.26900	3.38700
C	4.26600	1.76100	3.12500
C	3.91000	4.05900	2.16200
H	3.67400	5.80800	4.30700
H	3.94200	2.97900	5.47800
H	2.56500	3.39600	4.48600
H	4.63400	5.29700	5.70200
H	5.45700	3.50400	3.54500
H	3.21200	1.46300	3.05200
H	4.75200	1.47200	2.18900
H	4.72000	1.17100	3.92700
H	2.86000	3.82800	1.94300
H	3.99000	5.13999	2.30500

H	4.50100	3.80000	1.27800
C	4.18400	4.27500	-7.79300
C	3.43400	3.78600	-6.57400
C	3.81300	2.63100	-5.88100
C	2.32799	4.50000	-6.09200
C	3.11700	2.18600	-4.75300
C	1.62500	4.07700	-4.96900
C	2.01799	2.91400	-4.29600
O	1.24700	2.52300	-3.23600
H	4.88400	3.51800	-8.15800
H	3.50200	4.52600	-8.61100
H	4.67000	2.05600	-6.22000
H	4.76600	5.17900	-7.57100
H	2.00999	5.40400	-6.60600
H	3.43500	1.29500	-4.22400
H	0.77000	4.63199	-4.59400
H	1.57500	1.68900	-2.81000
C	0.20000	-1.66800	-7.19500
C	-0.77000	-0.60200	-6.67800
C	-0.29300	0.02700	-5.37900
N	-0.58800	1.32600	-5.17500
O	0.29300	-0.65900	-4.53000
H	-0.18700	-2.12400	-8.10900
H	1.17900	-1.23300	-7.41300
H	-0.94600	0.17400	-7.43100
H	-1.74600	-1.05700	-6.46300
H	-0.16100	1.79500	-4.37399
H	-0.89500	1.90500	-5.93900
H	0.33700	-2.44899	-6.44500
C	-5.35200	6.05400	-2.60200
C	-5.33800	6.28000	-1.08400
O	-5.35000	5.31700	-0.31600
C	-4.01400	5.39200	-2.98900
C	-3.92600	4.82500	-4.41399
C	-2.48300	4.38300	-4.69900
C	-4.89600	3.65500	-4.62600
H	-6.19100	5.38400	-2.80900
H	-3.21300	6.12800	-2.84200
H	-3.81600	4.58399	-2.27700
H	-4.18499	5.62300	-5.12600
H	-1.78200	5.21900	-4.60900
H	-2.16800	3.61100	-3.98900
H	-2.39299	3.97400	-5.71300
H	-4.79900	3.24400	-5.63600
H	-4.68800	2.84900	-3.91100
H	-5.94000	3.95000	-4.49300
N	-5.25200	7.56800	-0.64700
C	-5.21200	7.95700	0.76600
C	-4.03899	8.89500	1.08700
C	-2.68300	8.22800	1.08100
C	-2.28200	7.45400	2.17700
C	-1.79400	8.38299	0.01300
C	-1.01800	6.87100	2.21900
C	-0.52500	7.80100	0.04900
C	-0.13200	7.05000	1.15500
H	-5.14100	7.02800	1.33600
H	-4.04800	9.73700	0.38400
H	-4.22700	9.32199	2.07799
H	-2.96300	7.32200	3.01400
H	-2.08900	8.98300	-0.84500
H	-0.72500	6.27900	3.07700
H	0.15800	7.94000	-0.78400
H	0.85100	6.59000	1.18600
H	-5.27000	8.30400	-1.33600
C	-7.62700	4.10100	2.03100
C	-6.88500	4.63199	3.25800
C	-7.25800	2.65600	1.66100
C	-5.75000	2.49100	1.44000
S	-5.27000	1.07000	0.38600
C	-5.81200	1.73900	-1.22200
H	-8.71000	4.15900	2.20100
H	-7.22400	5.64000	3.51900
H	-7.04600	3.99100	4.13300
H	-5.80600	4.68300	3.07900
H	-7.59600	1.95700	2.43500
H	-7.79600	2.38400	0.74600
H	-5.34600	3.39000	0.97100
H	-5.22400	2.34400	2.38700
H	-6.90000	1.74200	-1.31300
H	-7.40200	4.74600	1.17400
H	-5.43400	2.75800	-1.33900
H	-5.39000	1.09700	-1.99700
C	-2.43800	2.54800	6.18100
C	-1.09300	2.71200	5.51900
C	-1.27200	3.22400	4.07400
C	-0.26600	1.42100	5.56500
C	0.02700	3.56800	3.34600
H	-2.34500	2.17300	7.20600
H	-2.97800	3.50200	6.22500
H	-0.53400	3.48200	6.07300
H	-1.81800	2.46800	3.49400
H	-1.91800	4.11100	4.09300
H	-0.20700	1.02499	6.58400
H	-0.71600	0.65300	4.92900
H	0.75700	1.57800	5.21400
H	0.65700	2.68500	3.20600
H	-0.17800	3.98000	2.35600
H	0.61000	4.30700	3.90900
H	-3.07500	1.84700	5.63100
C	3.16700	-1.74300	8.61100
C	3.87700	-0.92500	7.52400
C	4.64300	-1.84100	6.56200
C	2.87800	-0.05100	6.75800
H	3.87700	-2.35000	9.18300
H	2.63200	-1.09500	9.31400
H	4.60100	-0.26200	8.01900
H	3.94600	-2.51500	6.05300
H	5.15900	-1.26000	5.79000
H	5.38900	-2.44400	7.09400
H	2.13899	-0.67500	6.24700
H	2.34500	0.63200	7.42800
H	3.37800	0.54900	5.99200
H	2.43400	-2.42499	8.16100
C	5.57000	-3.58900	3.36800
N	4.54699	-2.73600	2.80000
C	4.42699	-2.51399	1.48800
N	3.37900	-1.81600	1.03000
N	5.35200	-2.94800	0.62000
H	5.51800	-4.61400	2.97800
H	6.57100	-3.18200	3.18600

H	3.72200	-2.51700	3.38600
H	2.62800	-1.58400	1.68800
H	3.17200	-1.87200	0.02200
H	6.11800	-3.50300	0.95900
H	5.15299	-2.93700	-0.40000
H	5.41300	-3.62500	4.44700
C	-5.45100	-1.12100	5.44100
C	-5.27900	-1.46800	3.95500
C	-3.81200	-1.55800	3.49600
C	-3.68900	-2.11100	2.07399
C	-3.11300	-0.20000	3.59700
H	-5.02700	-0.14399	5.69100
H	-6.50700	-1.11200	5.73100
H	-5.76500	-2.43400	3.76500
H	-5.80200	-0.73300	3.32700
H	-3.29800	-2.25599	4.17500
H	-4.14100	-1.43000	1.34700
H	-2.63700	-2.22400	1.79300
H	-4.17800	-3.08300	1.97200
H	-2.10600	-0.23500	3.17600
H	-3.66800	0.56000	3.03600
H	-3.03500	0.13600	4.63199
C	-2.87100	-7.05400	3.30900
C	-3.19200	-5.96100	2.28600
C	-3.11300	-6.40600	0.81400
C	-4.13699	-7.49900	0.48300
C	-3.29500	-5.20400	-0.11700
H	-3.59600	-7.87200	3.27600
H	-4.19600	-5.55700	2.47600
H	-2.49900	-5.12300	2.43200
H	-2.10600	-6.81700	0.64600
H	-4.07700	-7.77800	-0.57599
H	-3.98300	-8.40600	1.07400
H	-5.15299	-7.13700	0.67500
H	-2.56400	-4.42500	0.11900
H	-3.15800	-5.48300	-1.16500
H	-4.29500	-4.76999	-0.00500
C	2.41100	-7.61200	0.26000
C	2.29700	-6.11800	0.58299
C	0.83500	-5.73500	0.88100
C	2.92100	-5.29000	-0.54500
C	0.64400	-4.28000	1.30800
H	1.84100	-7.85700	-0.64500
H	3.45100	-7.90300	0.08200
H	2.87300	-5.92100	1.50100
H	0.21500	-5.93900	-0.00100
H	0.45900	-6.39400	1.67500
H	3.98900	-5.50300	-0.64600
H	2.45500	-5.52300	-1.50800
H	2.81700	-4.21800	-0.38500
H	0.90700	-3.58800	0.50200
H	-0.39600	-4.08399	1.59000
H	1.27800	-4.04000	2.16600
C	8.21899	0.87500	-6.34800
C	7.71900	1.77200	-5.20800
C	7.00700	1.01000	-4.12700
C	7.02400	-0.35100	-3.92600
C	6.16100	1.55500	-3.09100
C	5.70800	0.45600	-2.30100
C	5.71200	2.84700	-2.77100
N	6.26900	-0.68900	-2.82000
C	4.83200	0.62300	-1.22200
C	4.83000	3.01700	-1.71100
C	4.39600	1.91400	-0.94500
H	8.97000	0.16100	-5.99700
H	8.56199	2.33700	-4.78400
H	7.03700	2.53100	-5.61800
H	7.52600	-1.12200	-4.49099
H	6.03000	3.70100	-3.36300
H	5.98200	-1.63300	-2.56300
H	4.50200	-0.21800	-0.62700
H	4.46300	4.00900	-1.46900
H	3.71500	2.07500	-0.11500
C	3.75700	-3.89400	-4.63199
C	3.39800	-2.55300	-3.97600
C	3.67900	-2.53600	-2.47500
O	4.79200	-2.98400	-2.08000
O	2.79300	-2.04999	-1.69600
H	3.10800	-4.69299	-4.26000
H	4.01499	-1.75500	-4.41300
H	2.35400	-2.28500	-4.15700
C	-0.73400	-8.74100	-3.66800
C	-1.61600	-7.77800	-2.85800
C	-1.34700	-6.32700	-3.18600
C	-0.18800	-5.70000	-2.71200
C	-2.22600	-5.58300	-3.98100
C	0.08100	-4.36200	-2.99800
C	-1.95500	-4.24700	-4.28500
C	-0.80500	-3.63000	-3.79200
H	-0.94500	-9.78300	-3.40700
H	-2.67300	-8.00400	-3.04200
H	-1.44600	-7.94600	-1.78700
H	0.50400	-6.27100	-2.10200
H	-3.13200	-6.05200	-4.35500
H	0.96500	-3.88000	-2.59200
H	-2.65500	-3.68000	-4.89300
H	-0.60400	-2.58700	-4.00300
O	1.37100	0.08800	-2.19700
H	0.88100	-0.20800	-3.00400
H	2.03900	-0.63800	-2.06100
H	4.79100	-4.16200	-4.40200
H	3.63600	-3.84100	-5.71800
H	-0.90600	-8.61500	-4.74099
H	0.32700	-8.55100	-3.47700
H	8.66000	1.46000	-7.15800
H	-5.50900	6.98200	-3.16300
H	-6.15700	8.44500	1.03299
H	2.84700	5.32100	5.78200
H	7.63200	2.35300	6.29800
H	-4.93900	-1.88100	6.04800
H	2.01900	-8.23199	1.07400
H	-1.87800	-7.48200	3.12500
H	-2.87400	-6.65200	4.32800
H	7.36900	0.30300	-6.74700
C	0.04500	-3.36000	5.66300
C	-0.10500	-2.36800	4.50800
C	1.21500	-2.04199	3.82100
O	2.26399	-2.53500	4.27299
O	1.19900	-1.27800	2.77700
H	-0.93000	-3.57700	6.11200

H	0.48500	-4.29800	5.31500
H	-0.79300	-2.75000	3.74300
H	-0.54700	-1.42900	4.85700
H	0.70200	-2.95800	6.43700
C	-0.05900	-0.55300	-0.41800
O	-0.52000	-0.28999	1.38000
H	0.17200	-0.79400	2.06500
C	-0.36300	1.91600	0.27600
C	0.41800	2.91400	-0.31300
C	-1.70700	2.16299	0.57099
C	-0.14800	4.15000	-0.62200
H	1.45400	2.71500	-0.56499
C	-2.26399	3.40600	0.27800
H	-2.31500	1.38000	1.00499
C	-1.48800	4.40000	-0.32000
H	0.46000	4.91399	-1.09300
H	-3.30300	3.60400	0.50500
H	-1.92400	5.36900	-0.53300
C	-1.27400	-0.68700	-1.21700
C	-1.74600	0.35600	-2.03200
C	-1.96600	-1.90600	-1.17900
C	-2.89400	0.16800	-2.79400
H	-1.19300	1.28499	-2.09200
C	-3.14600	-2.06900	-1.89700
H	-1.58000	-2.71900	-0.57599
C	-3.60300	-1.03400	-2.71200
H	-3.22900	0.96100	-3.45500
H	-3.69000	-3.00300	-1.83900
H	-4.50800	-1.16700	-3.29900
H	0.58600	-1.41900	-0.32500
C	0.23900	0.56399	0.49900
H	1.29299	0.61600	0.78100
C	-8.85900	-7.29700	-0.36700
C	-7.69600	-6.65100	-1.14100
C	-7.33100	-5.24400	-0.71500
C	-7.28400	-4.86400	0.63300
C	-7.00100	-4.28300	-1.68100
C	-6.93500	-3.56400	1.00000
C	-6.64600	-2.98500	-1.31500
C	-6.61400	-2.61500	0.02900
H	-8.61900	-7.42900	0.69100
H	-6.81000	-7.29300	-1.04299
H	-7.93700	-6.64000	-2.21000
H	-7.53100	-5.58500	1.40700
H	-7.03100	-4.55900	-2.73200
H	-6.91500	-3.28800	2.04900
H	-6.40400	-2.25400	-2.08199
H	-6.34900	-1.60300	0.31400
H	-9.08900	-8.28500	-0.77800
H	-9.76200	-6.68000	-0.43000

LEH-P-TS-SS

C	8.02999	1.95000	5.37900
C	8.02399	0.42500	5.50300
C	8.36700	-0.32100	4.21000
S	7.05700	-0.26400	2.91400
C	7.66000	1.05200	1.79900
H	9.03700	2.34200	5.20899
H	7.39200	2.27000	4.54900
H	8.74000	0.11700	6.27700
H	7.03700	0.09000	5.84400
H	9.30100	0.04700	3.77100
H	8.51200	-1.38400	4.42600
H	8.65499	0.81500	1.41600
H	6.96200	1.09000	0.95900
H	7.67400	2.02599	2.29199
C	3.71200	5.15800	5.10300
C	3.34500	3.89200	4.31100
C	4.43300	3.20900	3.46900
C	3.86600	1.91800	2.86000
C	4.97800	4.13500	2.37500
H	3.91900	6.01700	4.45899
H	2.94800	3.15100	5.01700
H	2.51500	4.13900	3.64000
H	4.57800	4.99700	5.75400
H	5.26400	2.93600	4.13500
H	3.01800	2.14600	2.20299
H	4.61599	1.38100	2.27500
H	3.50300	1.23100	3.63200
H	4.16700	4.46500	1.71400
H	5.44700	5.03099	2.79500
H	5.71900	3.62500	1.75200
C	4.20800	4.22600	-7.82300
C	3.45100	3.80100	-6.58900
C	3.83300	2.68300	-5.83800
C	2.31999	4.51400	-6.17400
C	3.10200	2.26600	-4.72600
C	1.58200	4.11700	-5.06500
C	1.96900	2.98400	-4.34300
O	1.17000	2.60400	-3.30100
H	3.69400	3.91100	-8.74000
H	4.31700	5.31400	-7.87400
H	4.71500	2.11700	-6.12200
H	5.21000	3.78800	-7.84700
H	2.01100	5.39700	-6.73100
H	3.41600	1.40100	-4.15000
H	0.70900	4.67400	-4.73900
H	1.53200	1.79800	-2.84800
C	0.20800	-1.70300	-7.18600
C	-0.79500	-0.69900	-6.61000
C	-0.25800	0.00400	-5.37500
N	-0.65100	1.27800	-5.16500
O	0.46500	-0.60400	-4.57300
H	-0.22100	-2.21600	-8.05100
H	1.12700	-1.20000	-7.50400
H	-1.10000	0.03599	-7.36300
H	-1.70700	-1.22300	-6.29200
H	-0.20000	1.80800	-4.41700
H	-1.10000	1.79600	-5.90300
H	0.47200	-2.44600	-6.43200
C	-5.33600	6.06900	-2.67000
C	-5.51600	6.35400	-1.17100
O	-5.88000	5.46200	-0.40400
C	-3.92700	5.45800	-2.84300
C	-3.64200	4.77700	-4.19200
C	-2.13099	4.56700	-4.36200
C	-4.38199	3.43900	-4.30999
H	-6.11500	5.35700	-2.94900
H	-3.18600	6.25000	-2.67300

H	-3.77700	4.72400	-2.04599
H	-3.98800	5.44200	-4.99600
H	-1.59300	5.52200	-4.33200
H	-1.72600	3.93600	-3.56400
H	-1.90300	4.09199	-5.32300
H	-4.21900	2.98100	-5.29200
H	-4.01700	2.73800	-3.54900
H	-5.46300	3.54900	-4.17400
N	-5.15000	7.59200	-0.72500
C	-5.19800	8.00000	0.68300
C	-3.97300	8.82499	1.09200
C	-2.68700	8.04100	1.21500
C	-2.52199	7.12700	2.26500
C	-1.62000	8.24000	0.33300
C	-1.31700	6.45500	2.44700
C	-0.40700	7.56900	0.51200
C	-0.25000	6.68200	1.57400
H	-5.27500	7.08000	1.26600
H	-3.84000	9.65300	0.38600
H	-4.20300	9.28800	2.05900
H	-3.34200	6.95800	2.96000
H	-1.72900	8.94800	-0.48600
H	-1.20400	5.76000	3.27100
H	0.41300	7.74800	-0.17700
H	0.68800	6.15800	1.72000
H	-4.90299	8.29200	-1.41000
C	-7.65400	4.05100	2.04199
C	-6.88500	4.70000	3.19800
C	-7.17000	2.63800	1.67800
C	-5.68900	2.59200	1.29600
S	-5.14600	1.01899	0.52000
C	-5.81500	1.24800	-1.16300
H	-8.71899	3.99700	2.29900
H	-7.31900	5.66900	3.46400
H	-6.90000	4.07000	4.09600
H	-5.83700	4.87100	2.93000
H	-7.35100	1.94300	2.50800
H	-7.76800	2.27400	0.83400
H	-5.45400	3.40800	0.60900
H	-5.05000	2.70300	2.17800
H	-6.90400	1.16799	-1.18200
H	-7.56600	4.69299	1.15999
H	-5.51500	2.22100	-1.56000
H	-5.38100	0.46000	-1.78100
C	-2.45100	2.62900	6.14800
C	-1.10500	2.78500	5.48800
C	-1.29800	2.89800	3.96200
C	-0.15800	1.64000	5.86700
C	-0.03000	3.18900	3.16700
H	-2.37100	2.60700	7.24000
H	-3.12500	3.45200	5.88200
H	-0.65100	3.72700	5.83600
H	-1.75000	1.97200	3.59000
H	-2.03500	3.68800	3.76600
H	-0.04200	1.56200	6.95300
H	-0.55200	0.68400	5.50600
H	0.83900	1.77100	5.43700
H	0.67700	2.35600	3.21100
H	-0.26300	3.37200	2.11800
H	0.47800	4.07599	3.55800
H	-2.93800	1.69800	5.84000
C	3.13800	-1.65500	8.62600
C	3.99900	-0.95500	7.56500
C	4.65500	-1.97900	6.62900
C	3.17500	0.05400	6.75500
H	3.72400	-2.38000	9.20299
H	2.70600	-0.93400	9.32700
H	4.79699	-0.40600	8.08799
H	3.88200	-2.54900	6.10300
H	5.27100	-1.48100	5.87200
H	5.29300	-2.67700	7.18500
H	2.40899	-0.46200	6.16900
H	2.68100	0.78500	7.40700
H	3.81000	0.60500	6.05200
H	2.31100	-2.19700	8.15199
C	5.55000	-3.54900	3.40500
N	4.51700	-2.71900	2.82200
C	4.41399	-2.48700	1.51000
N	3.37000	-1.79100	1.04000
N	5.34700	-2.91800	0.65000
H	5.56400	-4.55600	2.96500
H	6.54100	-3.09300	3.28800
H	3.70500	-2.45600	3.40900
H	2.58599	-1.60200	1.67900
H	3.18700	-1.83500	0.02600
H	6.14600	-3.41800	0.99700
H	5.15400	-2.91200	-0.37000
H	5.34300	-3.64600	4.47000
C	-5.47000	-1.03899	5.43200
C	-5.29800	-1.43400	3.96200
C	-3.82600	-1.61400	3.55500
C	-3.68600	-2.28399	2.18900
C	-3.07400	-0.28100	3.57000
H	-5.02799	-0.06300	5.65600
H	-6.52400	-1.00699	5.72700
H	-5.82600	-2.38300	3.79700
H	-5.76800	-0.69900	3.29600
H	-3.35800	-2.27599	4.30100
H	-4.09900	-1.65500	1.39600
H	-2.63000	-2.44499	1.95600
H	-4.19800	-3.24900	2.15300
H	-2.05500	-0.39200	3.19500
H	-3.57400	0.44800	2.92300
H	-3.02100	0.14000	4.57599
C	-2.89900	-6.99500	3.35500
C	-3.24500	-6.08700	2.17300
C	-3.30300	-6.78900	0.80600
C	-4.41099	-7.84800	0.75000
C	-3.49100	-5.75600	-0.31100
H	-3.66200	-7.76100	3.52000
H	-4.21300	-5.59800	2.35300
H	-2.50700	-5.27800	2.11700
H	-2.33800	-7.29400	0.64600
H	-4.44600	-8.32499	-0.23600
H	-4.26499	-8.63800	1.49400
H	-5.38800	-7.38900	0.93500
H	-2.62900	-5.08600	-0.38000
H	-3.60400	-6.23300	-1.28899
H	-4.38500	-5.15100	-0.12800
C	2.38800	-7.59000	0.32300

C	2.22100	-6.11800	0.70900
C	0.74300	-5.79300	0.99600
C	2.83400	-5.21900	-0.37100
C	0.52300	-4.39700	1.57400
H	1.83200	-7.81600	-0.59600
H	3.43800	-7.83800	0.14000
H	2.77500	-5.94500	1.64400
H	0.15500	-5.90800	0.07700
H	0.35200	-6.53900	1.70300
H	3.91400	-5.37700	-0.45000
H	2.40700	-5.43800	-1.35400
H	2.67500	-4.16000	-0.17400
H	0.84100	-3.61500	0.87900
H	-0.53200	-4.22200	1.80700
H	1.09800	-4.26300	2.49400
C	8.23100	0.82700	-6.34200
C	7.73100	1.75100	-5.22300
C	6.99900	1.02000	-4.13199
C	6.94500	-0.34000	-3.93400
C	6.22000	1.60800	-3.07100
C	5.72900	0.53600	-2.26600
C	5.86900	2.92600	-2.73200
N	6.20300	-0.63700	-2.80900
C	4.91700	0.75500	-1.14700
C	5.05700	3.14800	-1.62700
C	4.58900	2.07399	-0.84200
H	8.97200	0.11400	-5.97000
H	8.57700	2.31100	-4.80000
H	7.06600	2.51200	-5.65300
H	7.38900	-1.13799	-4.51199
H	6.21700	3.75900	-3.33500
H	5.90700	-1.57100	-2.52599
H	4.55900	-0.06600	-0.53800
H	4.77700	4.16200	-1.35800
H	3.97700	2.27700	0.03000
C	3.75400	-3.91600	-4.59600
C	3.36700	-2.57900	-3.95100
C	3.67000	-2.51700	-2.45400
O	4.77500	-2.98100	-2.05500
O	2.81300	-1.96500	-1.68500
H	3.13100	-4.72900	-4.20800
H	3.94900	-1.76800	-4.41000
H	2.31500	-2.34100	-4.11900
C	-0.75100	-8.74499	-3.60300
C	-1.64200	-7.74400	-2.85000
C	-1.32300	-6.30000	-3.16600
C	-0.16900	-5.69900	-2.64700
C	-2.16100	-5.52800	-3.97900
C	0.13100	-4.36200	-2.90800
C	-1.85700	-4.19500	-4.25800
C	-0.71400	-3.60400	-3.72100
H	-1.00699	-9.77600	-3.34000
H	-2.69300	-7.94400	-3.08600
H	-1.53200	-7.90900	-1.77000
H	0.49400	-6.28700	-2.02199
H	-3.06500	-5.97500	-4.38699
H	1.01200	-3.90100	-2.47100
H	-2.52900	-3.60700	-4.87800
H	-0.48500	-2.56300	-3.91600
O	1.48800	0.22900	-2.24600

H	0.99900	-0.10800	-3.03600
H	2.12200	-0.51600	-2.05500
H	4.79800	-4.15599	-4.37800
H	3.61700	-3.87700	-5.68100
H	-0.86300	-8.62800	-4.68600
H	0.30500	-8.58600	-3.36200
H	8.68500	1.39400	-7.15900
H	-5.44900	6.97000	-3.28500
H	-6.11100	8.58200	0.86600
H	2.84200	5.38800	5.73700
H	7.61800	2.41100	6.29000
H	-4.96100	-1.79500	6.04600
H	2.01300	-8.25799	1.10700
H	-1.94300	-7.50800	3.18800
H	-2.80700	-6.41700	4.28000
H	7.38100	0.25500	-6.73900
C	-8.88100	-7.25400	-0.33300
C	-7.71300	-6.61800	-1.11000
C	-7.38600	-5.20200	-0.68300
C	-7.18400	-4.88100	0.66600
C	-7.26500	-4.17600	-1.62800
C	-6.87700	-3.57800	1.05600
C	-6.95400	-2.87100	-1.24100
C	-6.75800	-2.56400	0.10500
H	-8.65600	-7.33600	0.73400
H	-6.82100	-7.24600	-0.98800
H	-7.94400	-6.62100	-2.18000
H	-7.26900	-5.65600	1.42200
H	-7.42000	-4.40500	-2.68000
H	-6.73000	-3.35200	2.10600
H	-6.86700	-2.09100	-1.99200
H	-6.51200	-1.55300	0.41200
H	-9.08799	-8.26200	-0.70500
H	-9.79100	-6.65500	-0.43600
C	0.01799	-3.29000	5.68600
C	-0.14399	-2.32300	4.51100
C	1.16300	-2.00900	3.78900
O	2.23900	-2.36300	4.30999
O	1.10600	-1.38000	2.66300
H	-0.94500	-3.46600	6.17500
H	0.41300	-4.25200	5.34600
H	-0.85600	-2.70900	3.77300
H	-0.56399	-1.36900	4.84999
H	0.71900	-2.89000	6.42200
C	-0.34800	-0.96500	-0.25900
O	-0.69300	-0.75800	1.12999
H	0.05000	-1.11000	1.83400
C	-0.27300	1.65100	-0.16600
C	0.59400	2.74100	-0.38200
C	-1.65800	1.88000	-0.04200
C	0.07800	4.02200	-0.54300
H	1.66000	2.56500	-0.48000
C	-2.15600	3.17100	-0.15800
H	-2.33199	1.06600	0.18600
C	-1.29600	4.24099	-0.42500
H	0.74700	4.84999	-0.74700
H	-3.21900	3.34400	-0.03200
H	-1.69400	5.24500	-0.51600
C	-1.53500	-1.14700	-1.16500
C	-1.78400	-0.30200	-2.25000

C	-2.39000	-2.22700	-0.93300
C	-2.89000	-0.52100	-3.07400
H	-1.12500	0.52900	-2.45500
C	-3.50100	-2.44099	-1.74200
H	-2.18600	-2.90200	-0.11100
C	-3.75600	-1.58400	-2.81500
H	-3.06500	0.14700	-3.91000
H	-4.16200	-3.27500	-1.54500
H	-4.61900	-1.75500	-3.45100
H	0.36900	-1.78100	-0.38500
C	0.32100	0.33400	-0.11200
H	1.35300	0.27900	0.21600

41B-React

C	7.42000	0.74800	5.77600
C	7.26700	-0.72500	6.16800
C	7.69600	-1.73900	5.09100
C	7.70400	-3.15600	5.68000
C	6.79700	-1.68600	3.84800
H	6.92800	0.94700	4.82200
H	7.84100	-0.91500	7.08400
H	6.21800	-0.92300	6.42300
H	8.72300	-1.49500	4.78200
H	8.01600	-3.89700	4.93600
H	8.38400	-3.23300	6.53600
H	6.70200	-3.43300	6.03100
H	7.10100	-2.43800	3.11200
H	5.75100	-1.87600	4.11700
H	6.82100	-0.71300	3.35200
C	4.66800	5.23400	5.08900
C	3.99300	3.90300	4.74700
C	4.96600	2.92300	4.09500
S	4.33000	1.22100	3.84400
C	3.32400	1.42100	2.33599
H	5.06200	5.72300	4.19000
H	3.58300	3.44800	5.65500
H	3.14400	4.07500	4.07900
H	5.32300	3.30300	3.13000
H	5.84500	2.79600	4.73100
H	3.05400	0.40900	2.02500
H	2.41699	2.00100	2.51100
H	3.91200	1.88600	1.54100
C	7.14000	-0.15600	-7.80600
C	7.13600	0.78900	-6.58600
C	5.85700	0.78500	-5.77900
C	5.50200	-0.29099	-4.95600
C	4.99000	1.88300	-5.83100
C	4.34400	-0.26300	-4.18300
C	3.82500	1.92700	-5.06799
C	3.51500	0.86300	-4.21800
O	2.38000	0.96200	-3.45800
H	7.40900	-1.18100	-7.53600
H	7.98600	0.54300	-5.93600
H	7.31200	1.81200	-6.93600
H	6.15200	-1.15700	-4.88900
H	5.23800	2.72800	-6.46900
H	4.09900	-1.08800	-3.52500
H	3.16600	2.78800	-5.09500
H	2.35800	0.24100	-2.76700
C	2.46600	6.45500	-4.03000
C	3.74600	6.40400	-3.18500
C	4.46100	5.03800	-3.17300
C	5.90700	5.18100	-2.68200
C	3.72200	3.99800	-2.32100
H	1.76400	5.65900	-3.76400
H	4.44000	7.16600	-3.56200
H	3.52500	6.69000	-2.14900
H	4.49500	4.66399	-4.20500
H	6.42100	4.21400	-2.69800
H	6.47600	5.87800	-3.30600
H	5.93300	5.55700	-1.65200
H	4.23800	3.03500	-2.34400
H	3.67100	4.33100	-1.27499
H	2.70300	3.81500	-2.66900
C	-2.65800	5.89500	-3.98400
C	-3.21700	6.84800	-2.92800
O	-4.41600	6.96600	-2.68800
C	-1.71500	4.82000	-3.43500
C	-1.19400	3.86000	-4.51499
C	-0.09500	2.96500	-3.93900
C	-2.32500	3.01400	-5.11599
H	-3.52100	5.45200	-4.48100
H	-0.85300	5.29300	-2.94700
H	-2.22700	4.24099	-2.65800
H	-0.75100	4.46800	-5.31900
H	0.34500	2.33500	-4.71500
H	0.71500	3.55400	-3.50200
H	-0.48300	2.31300	-3.15100
H	-1.92500	2.27800	-5.82300
H	-2.85600	2.47500	-4.32500
H	-3.05500	3.62200	-5.65700
N	-2.28399	7.62000	-2.29100
C	-2.63600	8.49000	-1.18200
C	-1.41000	9.27000	-0.68800
C	-0.41300	8.40100	0.04700
C	0.78500	7.98400	-0.54400
C	-0.69200	7.97900	1.35600
C	1.68300	7.17300	0.15700
C	0.20100	7.17200	2.05700
C	1.39300	6.76400	1.45600
H	-1.31000	7.37500	-2.39299
H	-3.03500	7.89000	-0.35100
H	-0.93100	9.76900	-1.53800
H	-1.76500	10.06000	-0.01700
H	1.03200	8.31199	-1.55100
H	-1.61600	8.29800	1.83100
H	2.61100	6.86600	-0.31200
H	-0.03100	6.86200	3.07000
H	2.08500	6.12700	1.99600
C	-6.58200	5.91000	0.31300
C	-6.43500	4.47400	-0.19600
C	-5.21400	4.34600	-1.10600
S	-4.75600	2.63700	-1.57000
C	-6.22500	2.19700	-2.56100
H	-7.45900	6.02600	0.95700
H	-6.33700	3.78200	0.64800
H	-7.33800	4.17600	-0.74200
H	-5.33100	4.95300	-2.00700

H	-4.32700	4.73400	-0.59600
H	-6.05000	1.20600	-2.98100
H	-7.12600	2.15800	-1.94700
H	-6.36600	2.90900	-3.37900
C	-2.54599	4.91800	5.81500
C	-1.13999	4.39300	5.50400
C	-0.99800	4.10900	3.99800
C	-0.80100	3.17000	6.35900
C	0.42300	3.78300	3.54600
H	-3.29900	4.13199	5.67700
H	-0.42200	5.18900	5.75600
H	-1.66600	3.29000	3.71800
H	-1.35000	4.98400	3.43700
H	0.20900	2.81000	6.16200
H	-0.87200	3.39800	7.42800
H	-1.49700	2.34900	6.14900
H	0.47200	3.67200	2.46099
H	1.11000	4.58700	3.83500
H	0.79500	2.85800	3.99900
C	1.74300	-0.14800	9.34500
C	2.73500	0.27200	8.25400
C	3.41900	-0.95400	7.63700
C	2.03100	1.10400	7.18000
H	0.96800	-0.79500	8.91600
H	3.51000	0.90000	8.71599
H	4.09700	-0.65500	6.83100
H	3.99700	-1.50900	8.38500
H	2.67000	-1.62900	7.21000
H	2.70700	1.36300	6.36000
H	1.19700	0.53900	6.74600
H	1.62300	2.02500	7.60600
C	3.68200	-3.89200	4.85300
N	3.04400	-3.06400	3.85500
C	3.11400	-3.23200	2.52599
N	4.02299	-4.03599	1.96800
N	2.27300	-2.54700	1.74000
H	3.76000	-3.30600	5.77000
H	4.70000	-4.16000	4.56300
H	2.27800	-2.50700	4.22300
H	4.74899	-4.43300	2.53900
H	4.05999	-4.19600	0.93800
H	1.43100	-2.20500	2.18400
H	2.22900	-2.84700	0.74200
C	-1.56900	-1.95800	7.01000
C	-1.19700	-1.27099	5.68000
C	0.12200	-1.66800	5.07300
O	0.98200	-2.28700	5.67200
O	0.38700	-1.27000	3.81100
H	-0.97800	-1.58900	7.84700
H	-1.98000	-1.44100	4.93400
H	-1.16399	-0.18200	5.81200
C	-5.03200	-5.26400	4.35100
C	-4.80000	-4.18800	3.29500
C	-6.07600	-3.52700	2.78300
S	-5.76700	-2.38700	1.37900
C	-5.37100	-3.59300	0.07700
H	-5.74400	-6.01900	3.99500
H	-4.14499	-3.40800	3.69200
H	-4.26000	-4.63300	2.45500
H	-6.81500	-4.27100	2.46400

H	-6.54600	-2.91600	3.56000
H	-5.15599	-3.02300	-0.82500
H	-4.48200	-4.17600	0.31900
H	-6.21900	-4.25900	-0.11100
C	0.58800	-7.77300	2.10400
C	1.18800	-6.35400	2.02900
C	0.13300	-5.28000	1.96600
C	-0.42200	-4.76300	3.14200
C	-0.35900	-4.83000	0.73700
C	-1.45500	-3.83000	3.08400
C	-1.39800	-3.90100	0.67500
C	-1.94600	-3.39400	1.85200
H	0.04400	-8.02800	1.19000
H	1.82100	-6.19200	2.90900
H	1.84900	-6.28000	1.15799
H	-0.04400	-5.09300	4.10600
H	0.08200	-5.19299	-0.18200
H	-1.89300	-3.46200	4.00600
H	-1.77000	-3.57500	-0.29099
H	-2.75600	-2.67500	1.81100
C	9.50700	-3.36600	-5.01199
C	9.19300	-2.19300	-4.06500
C	8.01900	-2.43299	-3.15000
C	7.36000	-3.61800	-2.89900
C	7.32200	-1.42300	-2.38600
C	6.25200	-2.07399	-1.70500
C	7.46500	-0.03300	-2.24600
N	6.30600	-3.41100	-2.02900
C	5.34100	-1.37600	-0.90300
C	6.55800	0.66500	-1.45800
C	5.50800	-0.00100	-0.79400
H	9.94400	-4.21300	-4.47300
H	10.08600	-1.93300	-3.47900
H	8.97600	-1.30200	-4.67100
H	7.56200	-4.60900	-3.27900
H	5.64200	-4.10100	-1.68200
H	8.26300	0.49300	-2.76300
H	4.53099	-1.89300	-0.40000
H	6.65400	1.74200	-1.35400
H	4.80900	0.57099	-0.19100
C	3.43600	-5.99800	-3.18900
C	2.44400	-5.07000	-2.49100
C	2.96100	-4.33200	-1.25600
O	4.05100	-4.68200	-0.71900
O	2.19499	-3.42600	-0.79700
H	4.03599	-6.52300	-2.44000
H	2.01700	-4.32800	-3.17200
H	1.59500	-5.65400	-2.11700
C	-2.20699	-8.56400	-2.20000
C	-2.36600	-7.39800	-1.21500
C	-2.42400	-6.07900	-1.94400
C	-3.63000	-5.40800	-2.16200
C	-1.25000	-5.52100	-2.46200
C	-3.65300	-4.19400	-2.85200
C	-1.25800	-4.30100	-3.13100
C	-2.46900	-3.63300	-3.32600
H	-2.19700	-9.53200	-1.69000
H	-3.27900	-7.53600	-0.62400
H	-1.53200	-7.38600	-0.50200
H	-4.55600	-5.83300	-1.78300

H	-0.31400	-6.05100	-2.31900
H	-4.59400	-3.68100	-3.00400
H	-0.33300	-3.85700	-3.48000
H	-2.48500	-2.67100	-3.82200
C	-7.24300	-5.09700	-3.65800
C	-6.93700	-4.09300	-4.77200
C	-7.54600	-2.69500	-4.56400
C	-6.98100	-1.99600	-3.32100
C	-7.32400	-1.82300	-5.80500
H	-6.87600	-4.75399	-2.68500
H	-5.85000	-3.99200	-4.88900
H	-7.30700	-4.49700	-5.72300
H	-8.63000	-2.81800	-4.42199
H	-7.41500	-0.99900	-3.19800
H	-7.18000	-2.55100	-2.40099
H	-5.89600	-1.87400	-3.41100
H	-7.77900	-0.83400	-5.68600
H	-6.25200	-1.67600	-5.98700
H	-7.75200	-2.28600	-6.70100
C	-0.59600	-0.82900	0.11300
O	-0.85400	-0.44800	1.49100
C	-0.20100	1.72900	0.41900
C	0.79000	2.54800	-0.13800
C	-1.46700	2.26100	0.68000
C	0.52200	3.89100	-0.39700
C	-1.72500	3.60900	0.43300
C	-0.72900	4.43000	-0.09600
C	-1.68300	-0.62900	-0.88800
C	-1.31400	-0.30800	-2.20000
C	-3.03300	-0.76800	-0.56499
C	-2.28600	-0.14299	-3.18100
C	-4.00699	-0.60300	-1.55200
C	-3.63500	-0.29600	-2.86000
C	0.11800	0.30900	0.71800
H	1.76400	2.13099	-0.37800
H	-2.23800	1.61800	1.08100
H	1.29299	4.52100	-0.82200
H	-2.70300	4.01600	0.66600
H	-0.92300	5.48200	-0.27300
H	-0.26500	-0.18700	-2.43100
H	-3.32800	-0.99200	0.45300
H	-1.98400	0.11000	-4.19100
H	-5.05700	-0.68800	-1.29000
H	-4.38999	-0.16500	-3.62800
H	-0.03700	-1.75600	0.01200
H	1.14799	0.11800	1.01000
C	1.10800	-3.40100	-6.91100
C	-0.00300	-2.49600	-6.36200
C	0.55400	-1.66500	-5.21900
N	0.57599	-0.32600	-5.35100
O	0.97900	-2.23700	-4.20400
H	1.64100	-3.86000	-6.07400
H	-0.43400	-1.86600	-7.14700
H	-0.81000	-3.10500	-5.94100
H	1.08700	0.23300	-4.66099
H	0.32500	0.10400	-6.22700
C	-5.90500	1.39000	4.74200
C	-5.56500	0.59300	3.47800
C	-4.13500	0.01500	3.54600
C	-5.77900	1.43300	2.21300
C	-3.04800	1.06600	3.77000
H	-5.23900	2.25300	4.84999
H	-6.25300	-0.26200	3.42100
H	-4.09500	-0.73400	4.34999
H	-3.93600	-0.52500	2.61600
H	-5.51000	0.88100	1.30900
H	-6.82600	1.73600	2.12100
H	-5.17500	2.34700	2.23000
H	-2.04999	0.66600	3.57700
H	-3.17600	1.91200	3.09100
H	-3.06500	1.46100	4.79000
C	-10.53600	-3.19400	0.30900
C	-9.31800	-2.33199	-0.04000
C	-9.44400	-0.85800	0.39700
C	-9.32799	-0.70500	1.91900
C	-8.39800	0.01500	-0.30800
H	-10.76600	-3.17400	1.37800
H	-8.40800	-2.74800	0.40800
H	-9.17000	-2.36600	-1.12700
H	-10.44000	-0.50300	0.09200
H	-9.45500	0.34100	2.22000
H	-10.08100	-1.29600	2.45100
H	-8.34000	-1.03299	2.25700
H	-8.46700	1.05900	0.01600
H	-7.38500	-0.33400	-0.08100
H	-8.52999	-0.01000	-1.39500
H	-2.77400	5.72300	5.10900
H	-2.63300	5.29800	6.83800
H	5.50600	5.09100	5.77900
H	3.96200	5.92600	5.56000
H	2.72000	6.34800	-5.08900
H	1.94800	7.41300	-3.91100
H	8.46899	1.04299	5.67700
H	6.96700	1.39900	6.53200
H	-3.44900	9.17400	-1.45000
H	-2.13400	6.53400	-4.70400
H	-6.77100	-6.06300	-3.86200
H	-8.32100	-5.26400	-3.56700
H	-1.28400	-8.46800	-2.78000
H	-3.04700	-8.54200	-2.90200
H	-0.09900	-7.86700	2.95100
H	1.41500	-8.47800	2.24000
H	-5.43200	-4.84700	5.28100
H	-4.09700	-5.78600	4.58200
H	1.24200	0.70500	9.81400
H	2.24000	-0.71800	10.13800
H	-5.79500	0.77600	5.64300
H	-6.92600	1.78600	4.73000
H	-10.38200	-4.23800	0.02000
H	-11.41000	-2.82100	-0.23600
H	-1.46800	-3.04300	6.94600
H	-2.62500	-1.69300	7.13500
H	1.83300	-2.82600	-7.49400
H	0.72800	-4.21600	-7.53700
H	7.84300	0.22200	-8.55300
H	6.15100	-0.18000	-8.27399
H	-0.36500	-0.98100	3.24800
H	4.13199	-5.42800	-3.81400
H	2.92600	-6.72400	-3.82800
H	10.19100	-3.07400	-5.81200

H	8.55599	-3.69900	-5.44000
H	-6.66100	6.61800	-0.51800
H	-5.69300	6.18500	0.89000
H	3.12200	-4.80300	5.08700
O	1.82900	-1.04299	-1.95100
H	2.19400	-1.78200	-1.42300
H	1.45900	-1.51400	-2.73300

41B-TS-RR

C	7.42300	0.26600	5.86900
C	7.20600	-1.19800	6.26000
C	7.57800	-2.23200	5.18200
C	7.48000	-3.64900	5.76100
C	6.70400	-2.10700	3.92500
H	6.95300	0.48600	4.90800
H	7.77500	-1.41800	7.17300
H	6.14800	-1.33700	6.52300
H	8.62400	-2.05700	4.88900
H	7.75600	-4.40500	5.01800
H	8.13500	-3.77600	6.62900
H	6.45400	-3.86100	6.08900
H	6.95700	-2.88500	3.19600
H	5.64200	-2.20600	4.17699
H	6.82100	-1.14199	3.42800
C	4.77200	4.84400	5.43900
C	4.04399	3.54200	5.07900
C	4.94200	2.56800	4.30999
S	4.31600	0.84700	4.19800
C	3.03300	0.97400	2.90700
H	5.18000	5.33700	4.55000
H	3.69100	3.05300	5.99300
H	3.15100	3.76400	4.48500
H	5.16099	2.94000	3.30300
H	5.90100	2.46500	4.82599
H	2.37699	0.11100	3.03800
H	2.43800	1.87900	3.02400
H	3.47800	0.94900	1.90800
C	7.19400	0.17700	-7.74300
C	7.08600	1.08900	-6.50500
C	5.80000	0.97500	-5.71000
C	5.41700	-0.20600	-5.05800
C	4.96500	2.09000	-5.56900
C	4.26900	-0.26500	-4.26800
C	3.81800	2.05200	-4.77799
C	3.48700	0.88000	-4.09700
O	2.36400	0.91100	-3.30200
H	7.49600	-0.84100	-7.47900
H	7.94100	0.89600	-5.84200
H	7.18900	2.13200	-6.82600
H	6.03900	-1.09300	-5.13500
H	5.22700	3.01800	-6.07100
H	4.00800	-1.18100	-3.75000
H	3.19500	2.93000	-4.65400
H	2.31100	0.14399	-2.68700
C	2.64500	6.65300	-3.59900
C	3.94300	6.56000	-2.78600
C	4.68400	5.21400	-2.91800
C	6.15500	5.35300	-2.50900
C	4.01700	4.10300	-2.09900
H	1.95200	5.83800	-3.36900
H	4.61000	7.36900	-3.11100
H	3.74500	6.74700	-1.72300
H	4.65900	4.91700	-3.97700
H	6.68300	4.39900	-2.61700
H	6.67400	6.09900	-3.12000
H	6.24000	5.66300	-1.46000
H	4.51900	3.14400	-2.25100
H	4.06400	4.34400	-1.02899
H	2.96800	3.95800	-2.36700
C	-2.49100	6.20100	-3.60600
C	-3.03400	7.08600	-2.48600
O	-4.22900	7.17700	-2.21300
C	-1.55400	5.08500	-3.13200
C	-0.89200	4.31600	-4.28400
C	0.12900	3.32100	-3.73000
C	-1.93100	3.61100	-5.16600
H	-3.35700	5.80100	-4.13400
H	-0.76200	5.50500	-2.50100
H	-2.11000	4.38300	-2.49800
H	-0.35200	5.04500	-4.90700
H	0.64700	2.79300	-4.53500
H	0.88800	3.81800	-3.12100
H	-0.35400	2.58199	-3.08600
H	-1.44500	2.96300	-5.90500
H	-2.59400	2.99200	-4.55199
H	-2.55600	4.32200	-5.71500
N	-2.09400	7.82400	-1.82300
C	-2.42800	8.62400	-0.65500
C	-1.18300	9.32799	-0.10300
C	-0.20400	8.37299	0.54800
C	0.99700	8.00600	-0.07199
C	-0.49600	7.82600	1.80600
C	1.88300	7.12400	0.55300
C	0.38600	6.94800	2.43200
C	1.58100	6.59200	1.80400
H	-1.11900	7.60700	-1.97000
H	-2.84800	7.97400	0.12700
H	-0.69300	9.88800	-0.90700
H	-1.51600	10.06400	0.63700
H	1.25499	8.43200	-1.03899
H	-1.42200	8.10300	2.30500
H	2.81400	6.85900	0.06300
H	0.14700	6.54500	3.40900
H	2.26500	5.90200	2.28700
C	-6.43600	6.04600	0.66800
C	-6.33000	4.62500	0.10500
C	-5.09300	4.48800	-0.78300
S	-4.69200	2.78900	-1.33800
C	-6.16700	2.45400	-2.36100
H	-7.31600	6.16400	1.30700
H	-6.27700	3.89700	0.92200
H	-7.23000	4.38400	-0.47200
H	-5.16000	5.14600	-1.65200
H	-4.20400	4.80999	-0.23100
H	-6.04600	1.46000	-2.79300
H	-7.07600	2.45699	-1.75900
H	-6.25500	3.18700	-3.16800
C	-2.45100	4.64100	6.11700

C	-1.11400	4.01199	5.72100
C	-1.04099	3.84900	4.19299
C	-0.88600	2.69700	6.46800
C	0.26800	3.24700	3.68900
H	-3.27000	3.92300	5.99000
H	-0.30900	4.70600	6.01200
H	-1.88000	3.23400	3.85300
H	-1.18800	4.83300	3.73100
H	0.07800	2.25599	6.21900
H	-0.91500	2.84600	7.55200
H	-1.66300	1.96700	6.21300
H	0.34400	3.32200	2.60400
H	1.12500	3.77400	4.12500
H	0.35700	2.19099	3.95800
C	1.71100	-0.71700	9.35600
C	2.60800	-0.27600	8.19600
C	3.17300	-1.49400	7.45100
C	1.83400	0.63100	7.23900
H	0.91200	-1.37000	8.98000
H	3.45100	0.30000	8.60699
H	3.81900	-1.17900	6.62500
H	3.75800	-2.13499	8.12100
H	2.35800	-2.08800	7.02500
H	2.44899	0.93200	6.38600
H	0.95600	0.10500	6.84700
H	1.48300	1.53000	7.75500
C	3.59200	-4.22900	4.65599
N	2.92000	-3.31000	3.77100
C	2.96700	-3.37000	2.43800
N	3.88100	-4.10300	1.79000
N	2.08800	-2.63800	1.73700
H	3.72400	-3.73300	5.62000
H	4.59000	-4.49600	4.30199
H	2.06900	-2.90600	4.21500
H	4.59300	-4.57900	2.31500
H	3.91600	-4.17500	0.75900
H	1.28499	-2.30300	2.27199
H	2.01500	-2.87500	0.73900
C	-1.62800	-2.31300	6.90400
C	-1.27699	-1.56000	5.58600
C	-0.03400	-1.95100	4.79000
O	0.78100	-2.74500	5.29900
O	0.13200	-1.43100	3.61500
H	-1.02200	-1.98800	7.75000
H	-2.12000	-1.64100	4.88999
H	-1.19500	-0.48500	5.78800
C	-5.14700	-5.38000	4.04000
C	-4.88100	-4.26600	3.03200
C	-6.12600	-3.50300	2.58900
S	-5.80100	-2.37699	1.17400
C	-5.62000	-3.61300	-0.14900
H	-5.88200	-6.09500	3.65500
H	-4.17400	-3.54700	3.45200
H	-4.38800	-4.69800	2.15800
H	-6.93600	-4.18100	2.29700
H	-6.51000	-2.86500	3.39100
H	-5.48200	-3.06800	-1.08200
H	-4.74200	-4.24200	0.00500
H	-6.51900	-4.23100	-0.23000
C	0.43000	-7.87100	1.67000
C	1.06700	-6.46900	1.65000
C	0.02800	-5.38300	1.63300
C	-0.49900	-4.88800	2.83100
C	-0.49200	-4.91399	0.42400
C	-1.53500	-3.96100	2.81500
C	-1.53800	-3.99200	0.40200
C	-2.06000	-3.51300	1.60300
H	-0.12800	-8.07300	0.75100
H	1.70500	-6.35500	2.53399
H	1.72700	-6.37500	0.78000
H	-0.08500	-5.21400	3.78100
H	-0.07000	-5.26300	-0.51000
H	-1.93700	-3.59200	3.75100
H	-1.93600	-3.65600	-0.54900
H	-2.87700	-2.80100	1.60500
C	9.47800	-3.24400	-5.13600
C	9.17200	-2.09700	-4.15400
C	7.96400	-2.33900	-3.28300
C	7.29900	-3.52700	-3.06500
C	7.24100	-1.33800	-2.53100
C	6.15000	-1.99700	-1.89000
C	7.37900	0.05100	-2.36800
N	6.21400	-3.33000	-2.23100
C	5.21300	-1.31000	-1.10800
C	6.45100	0.73800	-1.59500
C	5.37900	0.06400	-0.97300
H	9.88100	-4.12100	-4.62100
H	10.05400	-1.88300	-3.53400
H	8.99600	-1.17800	-4.73200
H	7.51600	-4.51400	-3.44500
H	5.55700	-4.03000	-1.89200
H	8.19400	0.58299	-2.84900
H	4.38500	-1.83200	-0.63900
H	6.54700	1.81200	-1.46700
H	4.66800	0.63000	-0.37700
C	3.34200	-5.84700	-3.49800
C	2.37699	-4.91700	-2.75900
C	2.89400	-4.27400	-1.47000
O	3.98400	-4.65100	-0.96600
O	2.12200	-3.41200	-0.93000
H	3.92700	-6.42900	-2.77900
H	2.00000	-4.11700	-3.40500
H	1.49000	-5.47500	-2.43900
C	-2.35900	-8.34399	-2.68500
C	-2.49600	-7.21200	-1.65900
C	-2.50199	-5.86500	-2.33900
C	-3.67500	-5.12300	-2.50700
C	-1.30900	-5.34200	-2.84600
C	-3.64200	-3.86800	-3.11800
C	-1.26099	-4.08399	-3.44000
C	-2.43400	-3.33700	-3.56900
H	-2.38600	-9.33100	-2.21200
H	-3.42200	-7.34200	-1.08800
H	-1.67600	-7.24900	-0.93300
H	-4.61700	-5.52300	-2.14400
H	-0.40000	-5.92600	-2.75100
H	-4.55600	-3.29700	-3.22400
H	-0.32000	-3.67000	-3.77800
H	-2.40099	-2.34100	-3.99600
C	-7.31200	-4.68800	-3.95300

C	-6.96800	-3.67200	-5.04399
C	-7.55900	-2.26799	-4.82800
C	-7.01200	-1.59900	-3.56200
C	-7.29500	-1.38200	-6.05100
H	-6.95100	-4.37100	-2.96900
H	-5.87600	-3.58700	-5.13400
H	-7.32100	-4.05600	-6.01000
H	-8.64800	-2.37600	-4.71300
H	-7.42600	-0.59300	-3.43800
H	-7.25000	-2.16100	-2.65500
H	-5.92200	-1.50400	-3.62500
H	-7.73700	-0.38800	-5.92700
H	-6.21600	-1.24900	-6.20500
H	-7.70800	-1.82500	-6.96300
C	-0.41800	-0.93600	-0.18700
O	-0.91800	-0.43900	1.57300
C	-0.00700	1.53700	0.44300
C	1.10100	2.30200	0.06800
C	-1.25800	2.14800	0.57699
C	0.96300	3.67800	-0.12400
C	-1.39000	3.52100	0.38200
C	-0.27800	4.29200	0.04600
C	-1.52700	-0.60800	-1.10400
C	-1.25600	0.11900	-2.27199
C	-2.84600	-0.98800	-0.82000
C	-2.27199	0.38100	-3.18300
C	-3.86900	-0.65900	-1.70600
C	-3.58100	-0.00600	-2.90300
C	0.14800	0.07299	0.73600
H	2.06900	1.82800	-0.06400
H	-2.11700	1.55200	0.85700
H	1.82700	4.27400	-0.38800
H	-2.35900	3.98800	0.51700
H	-0.37200	5.36500	-0.07099
H	-0.24900	0.45700	-2.46700
H	-3.08000	-1.49000	0.10700
H	-2.03500	0.90100	-4.10300
H	-4.89300	-0.91300	-1.45600
H	-4.37200	0.21800	-3.61000
H	-0.15600	-1.97100	-0.01000
H	1.13900	-0.18500	1.10800
C	1.09000	-2.98400	-7.06700
C	-0.01700	-2.07700	-6.50900
C	0.49300	-1.29500	-5.31100
N	0.53300	0.04800	-5.39200
O	0.85400	-1.89800	-4.28700
H	1.58500	-3.50200	-6.24100
H	-0.41000	-1.40900	-7.28200
H	-0.84900	-2.68900	-6.14500
H	1.03099	0.54800	-4.65299
H	0.35900	0.51500	-6.26700
C	-5.87900	1.25699	4.82300
C	-5.58200	0.51100	3.51500
C	-4.17199	-0.11500	3.52600
C	-5.79300	1.42000	2.29900
C	-3.03900	0.89500	3.70100
H	-5.19100	2.09700	4.96200
H	-6.30100	-0.31700	3.43300
H	-4.12600	-0.85900	4.33399
H	-4.02400	-0.66500	2.59100
H	-5.54500	0.91300	1.36300
H	-6.83600	1.74900	2.23500
H	-5.16899	2.31800	2.35900
H	-2.06000	0.42200	3.63500
H	-3.07500	1.66300	2.92500
H	-3.09800	1.40000	4.66700
C	-10.58400	-2.95300	0.10700
C	-9.34000	-2.11500	-0.20100
C	-9.42400	-0.65900	0.29900
C	-9.31500	-0.57599	1.82700
C	-8.34600	0.21000	-0.36100
H	-10.81400	-2.97700	1.17600
H	-8.44600	-2.57700	0.23300
H	-9.18699	-2.10800	-1.28800
H	-10.40600	-0.26000	0.00300
H	-9.41000	0.45900	2.17300
H	-10.09000	-1.16399	2.32799
H	-8.33900	-0.95000	2.15600
H	-8.39300	1.24300	0.00100
H	-7.34600	-0.17200	-0.13200
H	-8.46299	0.22600	-1.44900
H	-2.65800	5.49200	5.46000
H	-2.46000	4.98100	7.15700
H	5.60300	4.64100	6.12300
H	4.09700	5.54900	5.93400
H	2.90100	6.60100	-4.66200
H	2.12400	7.60000	-3.42300
H	8.47899	0.54400	5.79100
H	6.97300	0.93100	6.61500
H	-3.22500	9.34000	-0.88400
H	-1.95000	6.87000	-4.28500
H	-6.85700	-5.66000	-4.16700
H	-8.39400	-4.83700	-3.87700
H	-1.42900	-8.25600	-3.25400
H	-3.19500	-8.26300	-3.38700
H	-0.25100	-7.98300	2.51799
H	1.24000	-8.60100	1.76700
H	-5.52800	-4.99099	4.99000
H	-4.22400	-5.93400	4.24300
H	1.22500	0.11600	9.87299
H	2.27300	-1.29099	10.10100
H	-5.77700	0.59800	5.69300
H	-6.89100	1.67500	4.83000
H	-10.46299	-3.98800	-0.23000
H	-11.44700	-2.52999	-0.41800
H	-1.54000	-3.39500	6.78900
H	-2.67800	-2.03399	7.04100
H	1.84600	-2.40400	-7.60200
H	0.69600	-3.75200	-7.74200
H	7.91000	0.58299	-8.46400
H	6.22800	0.11300	-8.25400
H	-0.55700	-0.92400	2.44000
H	4.05800	-5.27200	-4.09600
H	2.80900	-6.52400	-4.17000
H	10.18300	-2.94200	-5.91400
H	8.52300	-3.53000	-5.58800
H	-6.48200	6.78800	-0.13500
H	-5.54500	6.26600	1.26499
H	3.01100	-5.13999	4.83399
O	1.33100	-1.09600	-1.74700

H	1.74800	-1.95400	-1.44600
H	0.97400	-1.35700	-2.63100

41B-TS-SS

C	7.28000	-0.00500	6.00800
C	7.02500	-1.49000	6.27900
C	7.42200	-2.44499	5.13800
C	7.32700	-3.90000	5.61600
C	6.56600	-2.24100	3.88000
H	6.82300	0.29800	5.06300
H	7.55800	-1.78600	7.19200
H	5.95700	-1.63400	6.49000
H	8.47200	-2.24400	4.87500
H	7.61100	-4.60100	4.82400
H	7.97700	-4.08500	6.47800
H	6.30000	-4.13800	5.92100
H	6.84800	-2.95200	3.09500
H	5.50300	-2.38499	4.10500
H	6.66600	-1.23600	3.46400
C	4.76100	4.64600	5.57700
C	3.97200	3.37800	5.22200
C	4.83000	2.35800	4.46699
S	4.14799	0.65500	4.38999
C	2.91100	0.78000	3.05600
H	5.20500	5.10700	4.68600
H	3.59100	2.91400	6.13700
H	3.09300	3.63800	4.62000
H	5.05999	2.70200	3.45300
H	5.78700	2.23200	4.98200
H	2.21200	-0.05000	3.19000
H	2.35300	1.71400	3.11800
H	3.39300	0.69400	2.07799
C	7.30700	0.04800	-7.60700
C	7.26700	0.93800	-6.34700
C	5.96300	0.91200	-5.57900
C	5.55100	-0.21000	-4.84900
C	5.13699	2.04199	-5.56000
C	4.37899	-0.19700	-4.09600
C	3.96200	2.07300	-4.81300
C	3.59800	0.96200	-4.05100
O	2.46000	1.05300	-3.29100
H	7.54800	-0.99300	-7.37200
H	8.09200	0.65500	-5.68100
H	7.46300	1.97400	-6.64400
H	6.16600	-1.10400	-4.83700
H	5.42800	2.92300	-6.12600
H	4.09300	-1.06100	-3.50800
H	3.34200	2.96300	-4.78000
H	2.40000	0.29500	-2.64900
C	2.85400	6.60100	-3.48000
C	4.13000	6.48700	-2.63600
C	4.92000	5.17800	-2.84400
C	6.38700	5.35100	-2.43299
C	4.30199	3.99800	-2.08500
H	2.15400	5.78400	-3.27700
H	4.77900	7.33800	-2.87800
H	3.88700	6.58400	-1.57000
H	4.89900	4.93700	-3.91700
H	6.94800	4.42300	-2.58300
H	6.87700	6.14100	-3.01200
H	6.46400	5.61800	-1.37200
H	4.82800	3.06600	-2.30500
H	4.37399	4.17600	-1.00400
H	3.25100	3.83800	-2.33400
C	-2.29000	6.28900	-3.58800
C	-2.80900	7.09500	-2.40600
O	-3.96900	7.01600	-2.00300
C	-1.41700	5.11100	-3.13000
C	-0.78100	4.30100	-4.26900
C	0.23000	3.30000	-3.70300
C	-1.83200	3.57600	-5.11800
H	-3.16500	5.94400	-4.14100
H	-0.61300	5.49400	-2.49000
H	-2.01600	4.44200	-2.50100
H	-0.24000	5.00699	-4.91700
H	0.77100	2.79200	-4.50399
H	0.97500	3.78600	-3.06700
H	-0.26700	2.53799	-3.09500
H	-1.35600	3.00000	-5.91900
H	-2.40000	2.87400	-4.49600
H	-2.54199	4.26499	-5.58500
N	-1.89100	7.89600	-1.79300
C	-2.21900	8.68000	-0.61000
C	-0.97400	9.35800	-0.03200
C	0.02200	8.40700	0.60000
C	1.30900	8.24100	0.07400
C	-0.31400	7.70400	1.76700
C	2.24300	7.41800	0.70900
C	0.61200	6.87900	2.40000
C	1.89900	6.73700	1.87600
H	-0.93900	7.90300	-2.12900
H	-2.66700	8.01500	0.13800
H	-0.48300	9.94800	-0.81500
H	-1.31300	10.07499	0.72400
H	1.59500	8.78400	-0.82300
H	-1.30700	7.82100	2.19400
H	3.24300	7.32500	0.29900
H	0.33600	6.35300	3.30800
H	2.62300	6.10100	2.37400
C	-6.31900	6.19800	0.61000
C	-6.22700	4.75399	0.09400
C	-4.75500	4.41700	-0.15800
S	-4.42500	2.88300	-1.09200
C	-5.02299	3.42000	-2.73400
H	-7.21900	6.38600	1.20000
H	-6.64300	4.04300	0.81500
H	-6.80600	4.64300	-0.82900
H	-4.27400	5.24400	-0.68500
H	-4.24000	4.28400	0.80100
H	-4.59800	2.74400	-3.47700
H	-6.11300	3.37700	-2.79500
H	-4.68900	4.44100	-2.93200
C	-2.47600	4.63199	6.11800
C	-1.21200	3.88300	5.68600
C	-1.14399	3.79000	4.15100
C	-1.13799	2.51600	6.37000
C	0.06500	3.02400	3.61800
H	-3.35600	3.98300	6.02500

H	-0.33600	4.46699	6.01000
H	-2.06300	3.33400	3.76900
H	-1.13199	4.81200	3.74900
H	-0.23600	1.97100	6.09100
H	-1.13599	2.62300	7.46000
H	-2.00100	1.89800	6.10100
H	0.17500	3.16600	2.54199
H	0.98600	3.37300	4.09900
H	-0.01799	1.94900	3.80100
C	1.47900	-0.86900	9.37700
C	2.36100	-0.42400	8.20700
C	2.93900	-1.63500	7.46400
C	1.56300	0.46800	7.25400
H	0.67500	-1.52100	9.01300
H	3.19700	0.16700	8.60900
H	3.59400	-1.31200	6.64900
H	3.52000	-2.27500	8.14000
H	2.13200	-2.23300	7.02600
H	2.16800	0.78400	6.40100
H	0.69600	-0.07800	6.86500
H	1.19200	1.35800	7.77200
C	3.35400	-4.38300	4.67699
N	2.72200	-3.44300	3.78000
C	2.80200	-3.48300	2.44600
N	3.71700	-4.22400	1.80900
N	1.95800	-2.71700	1.73300
H	3.47600	-3.89400	5.64500
H	4.35200	-4.67199	4.34000
H	1.86600	-3.03100	4.20899
H	4.38699	-4.74700	2.34500
H	3.76500	-4.29800	0.77600
H	1.14599	-2.38200	2.25599
H	1.89400	-2.94900	0.73100
C	-1.85400	-2.35000	6.84700
C	-1.47600	-1.63000	5.51700
C	-0.22300	-2.04400	4.74600
O	0.56399	-2.85700	5.27100
O	-0.02600	-1.52100	3.57900
H	-1.25000	-2.01900	7.69200
H	-2.30900	-1.72300	4.81200
H	-1.39200	-0.55100	5.69800
C	-5.39900	-5.29200	3.88500
C	-5.10700	-4.18200	2.88200
C	-6.34800	-3.42400	2.42099
S	-5.99300	-2.24100	1.06700
C	-5.68800	-3.40900	-0.29500
H	-6.14800	-5.99000	3.49300
H	-4.40700	-3.46100	3.31300
H	-4.60100	-4.61500	2.01600
H	-7.13500	-4.10799	2.08000
H	-6.77000	-2.82300	3.23200
H	-5.51600	-2.81200	-1.19000
H	-4.79600	-4.01100	-0.11800
H	-6.55600	-4.05400	-0.45600
C	0.15300	-7.90900	1.59400
C	0.82000	-6.51900	1.56400
C	-0.19200	-5.40500	1.52400
C	-0.76500	-4.93000	2.71000
C	-0.63100	-4.87800	0.30600
C	-1.77200	-3.97200	2.67200
C	-1.64700	-3.92200	0.26200
C	-2.22300	-3.47100	1.45000
H	-0.40800	-8.10300	0.67500
H	1.45100	-6.40900	2.45400
H	1.49100	-6.44900	0.70000
H	-0.40900	-5.29800	3.66800
H	-0.16900	-5.20500	-0.61600
H	-2.20800	-3.62000	3.59900
H	-1.97900	-3.53100	-0.69300
H	-3.02100	-2.73800	1.42700
C	9.44900	-3.45900	-4.99400
C	9.16499	-2.32100	-3.99500
C	7.95100	-2.54800	-3.12800
C	7.23200	-3.71200	-2.95700
C	7.27900	-1.55000	-2.32700
C	6.16200	-2.18600	-1.71000
C	7.48100	-0.17700	-2.10400
N	6.16300	-3.50400	-2.10700
C	5.26200	-1.49300	-0.88900
C	6.58800	0.51700	-1.29600
C	5.49200	-0.13600	-0.69600
H	9.82300	-4.35500	-4.48800
H	10.05000	-2.13899	-3.36900
H	9.01200	-1.39000	-4.55800
H	7.40100	-4.69100	-3.38400
H	5.46400	-4.18300	-1.80800
H	8.31700	0.33900	-2.56800
H	4.41700	-2.00000	-0.43600
H	6.73200	1.58000	-1.12500
H	4.81200	0.43400	-0.06700
C	3.21600	-5.91200	-3.49700
C	2.26900	-4.96000	-2.76000
C	2.79400	-4.32900	-1.46600
O	3.85800	-4.75699	-0.94100
O	2.05799	-3.42400	-0.95200
H	3.77200	-6.51700	-2.77300
H	1.91300	-4.15100	-3.40500
H	1.36900	-5.50100	-2.44600
C	-2.56500	-8.26300	-2.81700
C	-2.68000	-7.13500	-1.78400
C	-2.64600	-5.78600	-2.45600
C	-3.79600	-5.00900	-2.61700
C	-1.43700	-5.29700	-2.96400
C	-3.73000	-3.75900	-3.23400
C	-1.35700	-4.04500	-3.56400
C	-2.51100	-3.27000	-3.69800
H	-2.62900	-9.25200	-2.35400
H	-3.61300	-7.24500	-1.22000
H	-1.86600	-7.19900	-1.05300
H	-4.74800	-5.38000	-2.24600
H	-0.54300	-5.90400	-2.86500
H	-4.62800	-3.16000	-3.33700
H	-0.40200	-3.65600	-3.89700
H	-2.45500	-2.28200	-4.13999
C	-7.39300	-4.46200	-4.13900
C	-6.99500	-3.46800	-5.23100
C	-7.54600	-2.04500	-5.03899
C	-7.04200	-1.40200	-3.74200
C	-7.18600	-1.16599	-6.24300
H	-7.04500	-4.14600	-3.15100

H	-5.89900	-3.41600	-5.29300
H	-7.33500	-3.84800	-6.20200
H	-8.64300	-2.11300	-4.98200
H	-7.39600	-0.37100	-3.64700
H	-7.37400	-1.94200	-2.85200
H	-5.94700	-1.37800	-3.72900
H	-7.60500	-0.15900	-6.14300
H	-6.09700	-1.06600	-6.33500
H	-7.55900	-1.59600	-7.17800
C	-0.68200	-0.77100	0.22200
O	-1.03000	-0.37700	1.56800
C	0.33200	1.62200	0.19200
C	1.53800	2.36000	0.18800
C	-0.89900	2.31199	0.06500
C	1.51300	3.74100	0.05800
C	-0.91000	3.69300	-0.03400
C	0.28999	4.41000	-0.04100
C	-1.75500	-0.50500	-0.80300
C	-1.37800	-0.12500	-2.09700
C	-3.11000	-0.62500	-0.48900
C	-2.34400	0.13300	-3.06500
C	-4.07599	-0.37700	-1.46500
C	-3.69800	0.00200	-2.75200
C	0.41200	0.20500	0.36900
H	2.48300	1.83400	0.26700
H	-1.83300	1.77200	0.08700
H	2.43600	4.30400	0.05100
H	-1.85900	4.21000	-0.11000
H	0.27500	5.49200	-0.11400
H	-0.32900	-0.01400	-2.33300
H	-3.41700	-0.87700	0.51700
H	-2.02999	0.43400	-4.05900
H	-5.12700	-0.44000	-1.20600
H	-4.45400	0.21100	-3.50100
H	-0.32500	-1.80000	0.17000
H	1.34700	-0.20200	0.74400
C	1.10800	-2.95400	-7.07700
C	0.02600	-2.03200	-6.49700
C	0.56000	-1.29099	-5.28000
N	0.59600	0.05700	-5.31700
O	0.93600	-1.92700	-4.28400
H	1.59500	-3.49500	-6.26100
H	-0.35200	-1.33500	-7.25300
H	-0.82200	-2.63100	-6.14700
H	1.11500	0.54200	-4.58100
H	0.41600	0.54600	-6.18000
C	-5.96800	1.35400	4.72400
C	-5.68100	0.57299	3.43700
C	-4.28800	-0.08600	3.48600
C	-5.85800	1.45900	2.19800
C	-3.12600	0.90400	3.49800
H	-5.25800	2.17800	4.85400
H	-6.41900	-0.24000	3.36900
H	-4.23700	-0.71700	4.38400
H	-4.18400	-0.76100	2.63100
H	-5.59600	0.92900	1.27899
H	-6.89600	1.79400	2.10300
H	-5.22800	2.35200	2.25700
H	-2.17000	0.39600	3.61800
H	-3.07000	1.47000	2.56400

H	-3.22800	1.61700	4.31900
C	-10.69400	-2.68100	-0.12300
C	-9.42500	-1.87700	-0.42100
C	-9.48199	-0.41000	0.05100
C	-9.43000	-0.30200	1.58100
C	-8.35399	0.41600	-0.57999
H	-10.93600	-2.69500	0.94400
H	-8.55000	-2.35200	0.03599
H	-9.25400	-1.89100	-1.50500
H	-10.44100	0.01300	-0.28799
H	-9.50100	0.74200	1.90400
H	-10.24600	-0.85200	2.06100
H	-8.48300	-0.70500	1.95500
H	-8.38500	1.45800	-0.24600
H	-7.37700	0.01200	-0.30000
H	-8.42200	0.41100	-1.67300
H	-2.64800	5.49400	5.46600
H	-2.42200	4.97400	7.15600
H	5.57400	4.41500	6.27300
H	4.11700	5.39200	6.05300
H	3.13000	6.55600	-4.53899
H	2.33300	7.54800	-3.30100
H	8.34500	0.24500	5.95300
H	6.84400	0.61200	6.80100
H	-2.99100	9.41900	-0.84800
H	-1.71900	6.95000	-4.25000
H	-6.96000	-5.44900	-4.33500
H	-8.48000	-4.58300	-4.08500
H	-1.62300	-8.19999	-3.37000
H	-3.38500	-8.15199	-3.53400
H	-0.53400	-7.99800	2.44000
H	0.94200	-8.66100	1.69800
H	-5.78200	-4.89600	4.83200
H	-4.49600	-5.87300	4.09900
H	1.00600	-0.02800	9.89400
H	2.05100	-1.44000	10.11700
H	-5.89200	0.70900	5.60700
H	-6.96900	1.79900	4.71699
H	-10.59900	-3.71800	-0.45900
H	-11.53500	-2.22900	-0.66000
H	-1.78800	-3.43600	6.75400
H	-2.89900	-2.04300	6.96600
H	1.87700	-2.37800	-7.60100
H	0.70800	-3.70400	-7.76700
H	8.04700	0.44200	-8.30899
H	6.33800	0.06200	-8.11600
H	-0.67300	-0.94900	2.36600
H	3.95600	-5.35300	-4.08000
H	2.67100	-6.56700	-4.18200
H	10.17200	-3.16200	-5.75700
H	8.49499	-3.71400	-5.46600
H	-6.27500	6.90900	-0.21900
H	-5.43300	6.38800	1.22500
H	2.74600	-5.28000	4.83399
O	1.69800	-0.97800	-1.83700
H	2.02599	-1.82100	-1.43500
H	1.29600	-1.31800	-2.67000

52A-React

O	1.95100	-0.71700	-1.87500
H	1.96100	-1.67000	-1.65400
H	1.53200	-0.71000	-2.76600
C	6.31000	-3.92500	5.87300
C	5.76600	-4.84900	4.76800
C	5.50000	-4.15000	3.42000
C	5.42000	-5.16600	2.27500
C	4.21100	-3.32100	3.48400
H	5.98500	-2.88800	5.73400
H	6.46800	-5.67800	4.61700
H	4.82500	-5.31100	5.09700
H	6.34000	-3.47300	3.20900
H	5.20600	-4.67400	1.32200
H	6.35700	-5.72100	2.16900
H	4.62200	-5.89400	2.46800
H	4.05400	-2.76600	2.55399
H	3.34500	-3.97500	3.62800
H	4.22400	-2.60400	4.30900
C	4.94299	1.05300	6.42600
C	4.55100	0.16000	5.24500
C	5.64400	0.03599	4.17900
S	5.80000	1.49200	3.06900
C	4.20700	1.35600	2.18600
H	5.39800	1.98700	6.08300
H	4.31700	-0.84600	5.62100
H	3.63200	0.52100	4.77500
H	6.63000	-0.08300	4.64200
H	5.48000	-0.83600	3.54000
H	4.29399	1.92200	1.26000
H	3.99900	0.31400	1.93400
H	3.37500	1.76000	2.76300
C	8.15100	0.65900	-6.83500
C	8.27800	1.08400	-5.35900
C	6.95800	1.12400	-4.61500
C	6.33000	-0.03599	-4.14499
C	6.31700	2.34700	-4.37899
C	5.12399	0.01400	-3.44700
C	5.10900	2.41800	-3.69100
C	4.51300	1.24900	-3.20800
O	3.33800	1.36700	-2.51500
H	8.00799	-0.42200	-6.93100
H	8.97600	0.41100	-4.84700
H	8.72800	2.08199	-5.31400
H	6.78900	-1.00600	-4.30600
H	6.78000	3.26700	-4.73100
H	4.67400	-0.89500	-3.06200
H	4.63400	3.37200	-3.49700
H	3.02200	0.48000	-2.18000
C	1.28600	-0.68900	-7.32700
C	0.37800	0.24700	-6.51700
C	0.94300	0.37100	-5.11200
N	1.39400	1.57300	-4.70700
O	0.99400	-0.63400	-4.38900
H	1.53200	-1.56300	-6.71800
H	0.27300	1.22300	-7.00000
H	-0.62000	-0.19100	-6.42200
H	1.96300	1.63100	-3.85800
H	1.42600	2.34700	-5.35200
C	5.16899	6.74300	-1.37200
C	6.28600	6.06400	-0.56799
C	6.58900	4.61800	-1.00899
C	7.96700	4.16700	-0.51300
C	5.51700	3.63600	-0.52400
H	4.27299	6.12000	-1.44300
H	7.19700	6.66800	-0.66600
H	6.04500	6.06600	0.50300
H	6.60100	4.59300	-2.10900
H	8.18600	3.14600	-0.84400
H	8.76099	4.82300	-0.88500
H	8.00799	4.17600	0.58299
H	5.71400	2.62400	-0.88500
H	5.51100	3.60900	0.57199
H	4.51300	3.90800	-0.86300
C	0.13300	7.66700	-1.88400
C	-0.32900	8.21400	-0.52900
O	-1.48900	8.52399	-0.28199
C	0.73000	6.25600	-1.78400
C	1.09600	5.63100	-3.13900
C	1.98400	4.40200	-2.93100
C	-0.15200	5.26200	-3.95100
H	-0.72700	7.70500	-2.55200
H	1.63500	6.28700	-1.16300
H	0.03000	5.59600	-1.25800
H	1.67200	6.37600	-3.70700
H	2.33100	4.00000	-3.88800
H	2.87500	4.64600	-2.34600
H	1.45300	3.60800	-2.39800
H	0.12300	4.81500	-4.91300
H	-0.76000	4.53300	-3.40300
H	-0.78200	6.13100	-4.16099
N	0.67400	8.37900	0.39000
C	0.40100	8.74499	1.77300
C	1.69500	8.80700	2.59100
C	2.25599	7.43000	2.88700
C	3.30800	6.88200	2.14299
C	1.69700	6.65700	3.91500
C	3.79700	5.60400	2.43000
C	2.18600	5.38500	4.20600
C	3.24400	4.85500	3.46700
H	1.57000	7.95400	0.19900
H	-0.25900	7.98900	2.22300
H	2.43600	9.42099	2.06500
H	1.47400	9.31800	3.53400
H	3.77200	7.46600	1.35100
H	0.87700	7.06600	4.49899
H	4.61500	5.19700	1.84900
H	1.74500	4.80700	5.01300
H	3.64200	3.87300	3.69800
C	-4.35200	6.94300	1.96100
C	-4.30400	5.69700	1.06800
C	-2.93800	5.60100	0.36600
C	-5.47000	5.68900	0.07199
C	-2.74700	4.33600	-0.46700
H	-5.33300	7.06100	2.43600
H	-4.41399	4.81300	1.71200
H	-2.79500	6.49200	-0.25600
H	-2.15300	5.65300	1.13199
H	-5.49800	4.76499	-0.51300
H	-6.43000	5.78400	0.59200

H	-5.38800	6.52900	-0.62900
H	-1.72100	4.24600	-0.83500
H	-2.95300	3.43100	0.11100
H	-3.40800	4.32400	-1.33800
C	-2.04599	2.73200	6.92400
C	-0.71100	1.99600	6.73400
C	-0.21300	2.18900	5.29100
C	-0.82200	0.51900	7.12200
C	1.18100	1.64000	4.99400
H	-2.84200	2.26900	6.33200
H	0.02600	2.46400	7.40400
H	-0.92900	1.72800	4.60300
H	-0.22500	3.26100	5.05500
H	0.14299	0.01300	7.05700
H	-1.19100	0.40400	8.14700
H	-1.51900	-0.00500	6.45700
H	1.46200	1.87000	3.96200
H	1.93400	2.08100	5.65900
H	1.22800	0.55300	5.11500
C	-0.05800	-4.33500	8.54299
C	1.28899	-3.96500	7.90100
C	1.74800	-5.05400	6.92200
C	1.22000	-2.60300	7.20500
H	-0.83900	-4.40599	7.77700
H	2.03500	-3.89400	8.70600
H	2.71400	-4.79800	6.47300
H	1.85400	-6.02200	7.42400
H	1.01699	-5.16500	6.11500
H	2.18500	-2.32700	6.76500
H	0.48200	-2.62000	6.39500
H	0.93100	-1.81500	7.90600
C	1.67400	-6.71600	3.25400
N	1.45900	-5.40200	2.68700
C	1.61000	-5.08600	1.38600
N	2.24800	-5.89900	0.53800
N	1.13599	-3.92200	0.94500
H	1.76700	-6.60300	4.33500
H	2.60300	-7.16500	2.89600
H	0.81800	-4.82800	3.23300
H	2.75800	-6.68700	0.89600
H	2.38300	-5.62500	-0.46100
H	0.47900	-3.43200	1.53700
H	1.20800	-3.70200	-0.07299
C	-3.26600	-4.03500	5.51500
C	-2.45699	-3.02700	4.67500
C	-1.17900	-3.53900	4.06600
O	-0.61900	-4.55600	4.43600
O	-0.59500	-2.80000	3.10400
H	-2.78400	-4.26700	6.46500
H	-3.07700	-2.61500	3.87400
H	-2.17800	-2.16400	5.29400
C	-6.03100	1.22000	4.55100
C	-5.54700	0.89100	3.13200
C	-4.37700	-0.11200	3.15100
C	-5.19000	2.15800	2.34800
C	-3.19000	0.33400	4.00600
H	-5.28600	1.79800	5.10900
H	-6.37800	0.41000	2.60100
H	-4.74700	-1.08200	3.51100
H	-4.04300	-0.27800	2.12300
H	-4.88199	1.91700	1.32600
H	-6.04800	2.83300	2.28399
H	-4.37200	2.71200	2.82200
H	-2.29000	-0.24500	3.78600
H	-2.94100	1.38100	3.81900
H	-3.40000	0.24000	5.07599
C	-2.06300	-8.00300	-1.05300
C	-1.09100	-6.93300	-0.51200
C	-1.76600	-5.63700	-0.14599
C	-2.37699	-5.48300	1.10500
C	-1.84800	-4.58300	-1.06300
C	-3.06500	-4.31400	1.41900
C	-2.54300	-3.41300	-0.75500
C	-3.15100	-3.27200	0.49300
H	-2.50500	-7.70300	-2.00800
H	-0.58299	-7.33800	0.37100
H	-0.30500	-6.74400	-1.25299
H	-2.31599	-6.28900	1.83300
H	-1.36000	-4.67400	-2.02500
H	-3.56300	-4.23000	2.38000
H	-2.61000	-2.62000	-1.49200
H	-3.71000	-2.37500	0.73200
C	8.68000	-4.01499	-5.23200
C	8.55300	-3.30400	-3.87500
C	7.24200	-3.55000	-3.17500
C	6.27500	-4.47900	-3.49200
C	6.72800	-2.81100	-2.04400
C	5.44000	-3.34400	-1.74500
C	7.21000	-1.73400	-1.28000
N	5.19600	-4.36599	-2.63500
C	4.63400	-2.82000	-0.72700
C	6.41600	-1.20900	-0.26800
C	5.13999	-1.74600	-0.00200
H	8.81300	-5.09400	-5.10600
H	9.39200	-3.59000	-3.22500
H	8.66600	-2.22200	-4.02799
H	6.26800	-5.22200	-4.27500
H	4.33900	-4.91399	-2.64600
H	8.18399	-1.30200	-1.49300
H	3.64800	-3.22100	-0.53000
H	6.76800	-0.36800	0.32200
H	4.53099	-1.30700	0.78000
C	1.95500	-5.12600	-4.92199
C	1.55700	-3.99800	-3.95900
C	1.83500	-4.24099	-2.47600
O	2.48600	-5.26500	-2.11700
O	1.36500	-3.37400	-1.67200
H	2.15200	-6.04100	-4.35700
H	2.02700	-3.04300	-4.22000
H	0.48700	-3.79100	-4.01100
C	-4.25000	-5.98400	-5.25300
C	-4.13000	-5.21400	-3.93300
C	-3.61100	-3.81400	-4.14400
C	-4.47700	-2.71800	-4.17199
C	-2.24300	-3.58600	-4.34100
C	-3.98500	-1.42400	-4.34300
C	-1.74300	-2.29700	-4.51100
C	-2.61900	-1.21000	-4.50399
H	-4.61000	-7.00500	-5.08900
H	-5.10799	-5.17199	-3.44000

H	-3.46200	-5.75000	-3.24900
H	-5.54300	-2.87900	-4.03899
H	-1.56100	-4.43200	-4.34100
H	-4.66500	-0.58199	-4.32900
H	-0.68000	-2.12000	-4.62100
H	-2.23500	-0.20200	-4.59400
C	-10.91800	-0.15100	-2.39500
C	-9.71100	-0.52900	-1.52100
C	-9.44300	0.43200	-0.38600
C	-9.80800	0.11700	0.92600
C	-8.79700	1.65200	-0.62400
C	-9.51500	0.98400	1.98000
C	-8.50200	2.52300	0.42300
C	-8.85800	2.18900	1.73100
H	-11.83700	-0.11300	-1.80200
H	-9.86200	-1.53500	-1.11300
H	-8.81800	-0.58099	-2.15499
H	-10.30899	-0.82700	1.12700
H	-8.50100	1.90700	-1.63700
H	-9.79700	0.71800	2.99500
H	-7.98300	3.45500	0.22100
H	-8.62400	2.86300	2.54999
C	-0.81800	-0.73600	-0.06700
O	-1.11300	-0.76300	1.36000
C	0.30500	1.29800	1.07000
C	1.41700	1.92300	0.49100
C	-0.63800	2.06599	1.75600
C	1.58800	3.30100	0.62400
C	-0.45200	3.44000	1.90100
C	0.66100	4.06300	1.33800
C	-1.66200	0.15300	-0.92200
C	-1.05100	0.85100	-1.97100
C	-3.03500	0.29400	-0.71200
C	-1.81000	1.67300	-2.80100
C	-3.79500	1.11500	-1.54600
C	-3.18500	1.80200	-2.59700
C	0.13500	-0.17200	0.90300
H	2.13200	1.33800	-0.07600
H	-1.51100	1.57800	2.16800
H	2.45600	3.77800	0.18400
H	-1.18300	4.02500	2.45000
H	0.81100	5.13000	1.45800
H	0.01500	0.74600	-2.12100
H	-3.51400	-0.22800	0.10700
H	-1.32000	2.21099	-3.60700
H	-4.85799	1.23300	-1.36100
H	-3.77600	2.44700	-3.24000
H	-0.57899	-1.71200	-0.48200
H	1.01099	-0.78000	1.12700
C	-7.53300	-0.88200	-6.17800
C	-6.61500	0.31900	-6.41600
C	-6.93500	1.56800	-5.57400
C	-6.91200	1.30300	-4.06200
C	-5.96600	2.70400	-5.92400
H	-7.44300	-1.26499	-5.15599
H	-5.57500	0.02100	-6.23500
H	-6.66500	0.60200	-7.47500
H	-7.95300	1.89300	-5.83700
H	-7.15300	2.21600	-3.50900
H	-7.63000	0.53400	-3.76500
H	-5.91900	0.97800	-3.73200
H	-6.20600	3.62000	-5.37400
H	-4.93600	2.42300	-5.66900
H	-5.99000	2.93600	-6.99400
C	-7.14100	-4.93100	1.70000
C	-6.41100	-3.65800	1.26699
C	-7.30300	-2.46200	0.94100
S	-6.36800	-1.07500	0.18800
C	-5.98500	-1.81000	-1.43100
H	-7.91800	-5.21000	0.98000
H	-5.70100	-3.35500	2.04300
H	-5.80500	-3.89800	0.39000
H	-8.11000	-2.74200	0.25400
H	-7.77300	-2.04500	1.83700
H	-5.45200	-1.05300	-2.00300
H	-5.33300	-2.68200	-1.34800
H	-6.90300	-2.08000	-1.96000
H	0.89100	8.37800	-2.23000
H	-0.17900	9.67200	1.82000
H	-1.91200	3.75800	6.56500
H	-2.36200	2.75500	7.97100
H	5.65300	0.51900	7.06500
H	4.07100	1.30300	7.04000
H	-0.37100	-3.59600	9.28700
H	0.02200	-5.31200	9.03100
H	7.40400	-3.93600	5.91000
H	5.96300	-4.24000	6.86300
H	9.52200	-3.63200	-5.81300
H	7.75200	-3.86400	-5.79400
H	2.86000	-4.88500	-5.48600
H	2.22300	-0.19400	-7.59700
H	1.15300	-5.34100	-5.63600
H	0.81400	-1.06200	-8.24200
H	9.02800	0.95400	-7.42000
H	7.28400	1.14599	-7.29200
H	-3.28200	-6.04200	-5.76000
H	-4.94800	-5.47900	-5.92900
H	-2.87200	-8.20200	-0.34400
H	-1.48100	-8.92000	-1.19500
H	0.83800	-7.39400	3.05200
H	-3.45100	-4.96200	4.96900
H	-4.21300	-3.51100	5.68100
H	-6.23500	0.30200	5.11200
H	-6.95200	1.81100	4.52200
H	-11.05100	-0.89000	-3.19200
H	-10.77700	0.83100	-2.85600
H	5.53300	6.94700	-2.38499
H	4.87899	7.70200	-0.93000
H	-7.28200	-1.70400	-6.85500
H	-8.58300	-0.62100	-6.34400
H	-7.61300	-4.83200	2.68300
H	-6.41300	-5.74800	1.73400
H	-4.14100	7.84700	1.37900
H	-3.60200	6.87800	2.75600
H	-1.11900	-2.08700	2.66700

52A-TS-RR

O	1.28600	-0.99400	-1.64800

H	1.32700	-1.99200	-1.59200
H	1.02899	-0.85400	-2.58900
C	6.09300	-3.92400	6.09800
C	5.51800	-4.84100	5.00300
C	5.40400	-4.19100	3.60900
C	5.31000	-5.25000	2.50500
C	4.19100	-3.25300	3.53600
H	5.80900	-2.87800	5.94100
H	6.13700	-5.74500	4.93600
H	4.51700	-5.18499	5.29700
H	6.31400	-3.59900	3.43100
H	5.20099	-4.78700	1.51900
H	6.19800	-5.89100	2.48700
H	4.43600	-5.89300	2.66700
H	4.16200	-2.72600	2.57799
H	3.26000	-3.82400	3.63000
H	4.20099	-2.50700	4.33500
C	4.91600	1.12100	6.43900
C	4.56799	0.23200	5.24400
C	5.70100	0.11500	4.22000
S	5.93300	1.61800	3.18600
C	4.42600	1.49200	2.17099
H	5.38700	2.05500	6.11700
H	4.32100	-0.77400	5.60600
H	3.66800	0.59700	4.74300
H	6.66300	-0.05000	4.71600
H	5.53700	-0.72600	3.54000
H	4.45800	2.29700	1.44000
H	4.39600	0.53800	1.63900
H	3.51800	1.61000	2.76300
C	8.24000	0.07600	-6.75700
C	8.28700	0.60000	-5.31000
C	6.93900	0.61800	-4.60500
C	6.22500	-0.55300	-4.31400
C	6.37100	1.83200	-4.19800
C	5.00600	-0.52200	-3.63300
C	5.15700	1.88200	-3.51400
C	4.47600	0.70000	-3.21600
O	3.28400	0.81700	-2.53399
H	8.09800	-1.01000	-6.78100
H	8.99799	-0.00400	-4.73100
H	8.68600	1.62000	-5.30800
H	6.62800	-1.52000	-4.59600
H	6.89500	2.76200	-4.40500
H	4.49800	-1.44900	-3.38600
H	4.74500	2.82800	-3.18500
H	2.92700	-0.04500	-2.23300
C	1.33200	-1.02400	-7.27500
C	0.44600	-0.02100	-6.52100
C	0.98000	0.15700	-5.11100
N	1.46000	1.36400	-4.75399
O	0.97900	-0.80300	-4.32599
H	1.53700	-1.88100	-6.62800
H	0.38300	0.93500	-7.05100
H	-0.56799	-0.42000	-6.42700
H	2.01100	1.41500	-3.89600
H	1.55500	2.10100	-5.43400
C	5.44400	6.48300	-1.57400
C	6.58400	5.93200	-0.70800
C	6.99600	4.49099	-1.07500
C	8.41100	4.17400	-0.57899
C	6.00400	3.46400	-0.51700
H	4.58900	5.80200	-1.61500
H	7.45100	6.59500	-0.81800
H	6.31700	5.96600	0.35500
H	6.99500	4.41000	-2.17300
H	8.69900	3.15000	-0.83600
H	9.14899	4.85500	-1.01600
H	8.46899	4.26999	0.51100
H	6.22100	2.45400	-0.87700
H	6.06500	3.44900	0.57699
H	4.97100	3.69400	-0.79600
C	0.45300	7.58100	-2.18000
C	-0.00300	8.18000	-0.84500
O	-1.16300	8.50100	-0.61000
C	0.99300	6.15100	-2.02999
C	1.45700	5.50700	-3.34500
C	2.21600	4.20899	-3.05900
C	0.28399	5.25200	-4.30000
H	-0.39900	7.63000	-2.85800
H	1.84000	6.15300	-1.33100
H	0.22600	5.51800	-1.56600
H	2.15300	6.20600	-3.83400
H	2.62300	3.77700	-3.97800
H	3.05900	4.38300	-2.38300
H	1.56900	3.46600	-2.58100
H	0.62200	4.75500	-5.21600
H	-0.46200	4.60799	-3.82000
H	-0.21800	6.17700	-4.59600
N	0.99900	8.36800	0.06800
C	0.72600	8.79200	1.43500
C	2.01500	8.82799	2.26300
C	2.52800	7.44200	2.60600
C	3.61900	6.86600	1.94500
C	1.89300	6.69700	3.61200
C	4.07500	5.59100	2.29400
C	2.34900	5.42900	3.96500
C	3.45100	4.87300	3.31200
H	1.90000	7.95000	-0.11200
H	0.03100	8.07900	1.89900
H	2.78300	9.39899	1.72700
H	1.80600	9.37400	3.18800
H	4.13800	7.42800	1.17200
H	1.04200	7.12700	4.13300
H	4.92699	5.16300	1.77800
H	1.85100	4.87399	4.75500
H	3.82500	3.89600	3.59600
C	-4.09600	7.18500	1.63900
C	-4.09000	5.88800	0.82100
C	-2.68100	5.63200	0.26000
C	-5.15900	5.92700	-0.27800
C	-2.52999	4.32100	-0.50700
H	-5.07599	7.37900	2.08700
H	-4.33900	5.05800	1.49700
H	-2.39699	6.47700	-0.37800
H	-1.97100	5.64300	1.09800
H	-5.23200	4.97200	-0.80900
H	-6.14600	6.14800	0.14299
H	-4.92900	6.70600	-1.01499
H	-1.48900	4.13699	-0.78700

H	-2.85500	3.46000	0.08600
H	-3.12000	4.32599	-1.42800
C	-2.00500	3.08800	6.79000
C	-0.70400	2.28200	6.69300
C	-0.15000	2.34500	5.25800
C	-0.90600	0.84200	7.17300
C	1.23600	1.73200	5.06600
H	-2.80000	2.62700	6.19300
H	0.03599	2.75800	7.35500
H	-0.85500	1.85000	4.58399
H	-0.11800	3.39600	4.94000
H	0.03100	0.28299	7.18300
H	-1.31900	0.81800	8.18800
H	-1.60400	0.30900	6.51700
H	1.57100	1.86400	4.03300
H	1.97500	2.20600	5.72300
H	1.24100	0.65900	5.28000
C	-0.31300	-3.98100	8.70900
C	0.97300	-3.78300	7.89100
C	1.17900	-4.94299	6.90900
C	0.96400	-2.44400	7.14700
H	-1.18800	-3.99200	8.04900
H	1.81900	-3.77300	8.59399
H	2.10800	-4.81400	6.34200
H	1.23300	-5.90400	7.43400
H	0.35000	-4.98299	6.19400
H	1.89100	-2.29599	6.58100
H	0.13300	-2.40300	6.43400
H	0.85600	-1.60700	7.84400
C	1.37700	-6.63600	3.53800
N	1.18000	-5.31300	2.99100
C	1.34300	-5.01800	1.69200
N	1.99100	-5.84000	0.85700
N	0.87600	-3.85100	1.24300
H	1.49600	-6.54100	4.61900
H	2.28700	-7.10900	3.16000
H	0.43200	-4.78500	3.48900
H	2.41100	-6.67900	1.21500
H	2.13099	-5.61700	-0.14599
H	0.20800	-3.36200	1.84900
H	0.90200	-3.70700	0.22500
C	-3.47600	-3.67700	5.63500
C	-2.63500	-2.71600	4.75000
C	-1.42100	-3.26200	4.00500
O	-0.96800	-4.37700	4.33500
O	-0.89700	-2.53600	3.07400
H	-3.00700	-3.88800	6.59600
H	-3.28200	-2.25500	3.99700
H	-2.27700	-1.87700	5.36000
C	-6.02300	1.63900	4.43200
C	-5.56100	1.24400	3.02200
C	-4.40800	0.22200	3.06200
C	-5.19000	2.47400	2.18800
C	-3.21100	0.66000	3.90700
H	-5.25200	2.20200	4.96800
H	-6.40800	0.76100	2.51900
H	-4.79300	-0.73200	3.44700
H	-4.08000	0.02500	2.03600
H	-4.90700	2.18600	1.17000
H	-6.03100	3.17000	2.11400

H	-4.34800	3.02000	2.62800
H	-2.35100	0.00700	3.74900
H	-2.90100	1.67700	3.65200
H	-3.44500	0.65000	4.97499
C	-2.36400	-7.94700	-0.75800
C	-1.34700	-6.89200	-0.27300
C	-1.97900	-5.55800	0.02400
C	-2.57300	-5.31700	1.26800
C	-2.04100	-4.56100	-0.95300
C	-3.23500	-4.12000	1.51600
C	-2.71100	-3.36100	-0.71300
C	-3.31100	-3.13800	0.52700
H	-2.79900	-7.67600	-1.72500
H	-0.85400	-7.27000	0.63100
H	-0.55700	-6.77200	-1.02299
H	-2.50599	-6.06700	2.05100
H	-1.55600	-4.71800	-1.90900
H	-3.69800	-3.95400	2.48200
H	-2.76800	-2.61500	-1.49900
H	-3.85300	-2.22000	0.72700
C	8.56900	-4.54699	-4.96699
C	8.43699	-3.76200	-3.64800
C	7.09800	-3.91600	-2.97300
C	6.12500	-4.85900	-3.23200
C	6.55400	-3.05800	-1.94400
C	5.24500	-3.53900	-1.64500
C	7.02100	-1.91100	-1.27899
N	5.01600	-4.64300	-2.43699
C	4.40299	-2.89600	-0.72800
C	6.19200	-1.26699	-0.36800
C	4.89400	-1.75300	-0.10400
H	8.65600	-5.62300	-4.78500
H	9.24700	-4.04500	-2.96100
H	8.59399	-2.69400	-3.85300
H	6.13600	-5.67900	-3.93500
H	4.15200	-5.18100	-2.41800
H	8.01099	-1.51900	-1.49000
H	3.40500	-3.26200	-0.52400
H	6.54000	-0.37500	0.14499
H	4.25500	-1.23200	0.60100
C	1.80200	-5.38400	-4.69000
C	1.41600	-4.22200	-3.76400
C	1.64400	-4.44700	-2.27100
O	2.29199	-5.45100	-1.87300
O	1.12999	-3.57900	-1.48900
H	1.97200	-6.28800	-4.09900
H	1.92800	-3.29000	-4.03200
H	0.35500	-3.98300	-3.85000
C	-4.42699	-6.01300	-5.05900
C	-4.28200	-5.17600	-3.78400
C	-3.68900	-3.81600	-4.05500
C	-4.48900	-2.67100	-4.10500
C	-2.31199	-3.67100	-4.26499
C	-3.92200	-1.40900	-4.28599
C	-1.73800	-2.41400	-4.44600
C	-2.54400	-1.27499	-4.43800
H	-4.83800	-7.00400	-4.84199
H	-5.26200	-5.05800	-3.30800
H	-3.65100	-5.70400	-3.06000
H	-5.56300	-2.76700	-3.97300

H	-1.68100	-4.55600	-4.25699
H	-4.55100	-0.52900	-4.28300
H	-0.66800	-2.30100	-4.56300
H	-2.09800	-0.29099	-4.52500
C	-10.89000	0.18200	-2.50999
C	-9.85300	-0.29800	-1.48400
C	-9.55599	0.69200	-0.38000
C	-9.88700	0.41200	0.94900
C	-8.90300	1.89800	-0.66300
C	-9.55300	1.29800	1.97400
C	-8.56800	2.78800	0.35500
C	-8.88899	2.48900	1.68100
H	-11.85200	0.38800	-2.02999
H	-10.19500	-1.24000	-1.03800
H	-8.91600	-0.52800	-2.00500
H	-10.39500	-0.51900	1.18400
H	-8.63299	2.12900	-1.68900
H	-9.81000	1.05800	3.00200
H	-8.04400	3.71000	0.11800
H	-8.62400	3.17800	2.47600
C	-0.59800	-0.75600	-0.24100
O	-1.17000	-0.58600	1.55100
C	0.48200	1.17300	1.03899
C	1.62800	1.63300	0.38000
C	-0.34600	2.07900	1.70300
C	1.93000	2.99400	0.38700
C	-0.02100	3.43500	1.73100
C	1.11200	3.90000	1.06700
C	-1.43100	0.15100	-1.06000
C	-0.83800	1.04700	-1.96300
C	-2.82800	0.13100	-0.93500
C	-1.63300	1.85300	-2.77200
C	-3.61300	0.98500	-1.70500
C	-3.02200	1.82800	-2.64600
C	0.11600	-0.28299	0.96400
H	2.26700	0.94100	-0.15600
H	-1.24000	1.71400	2.18900
H	2.81400	3.34500	-0.13100
H	-0.66000	4.12899	2.26600
H	1.36300	4.95400	1.08900
H	0.23800	1.09500	-2.04000
H	-3.29500	-0.51400	-0.20600
H	-1.15599	2.50900	-3.49200
H	-4.68800	0.99500	-1.56200
H	-3.63900	2.47500	-3.26000
H	-0.69100	-1.82800	-0.37200
H	0.90000	-0.96000	1.30900
C	-7.49800	-0.82900	-6.22200
C	-6.54300	0.35500	-6.38600
C	-6.86700	1.58600	-5.51900
C	-6.92400	1.27099	-4.01900
C	-5.84900	2.70200	-5.78600
H	-7.45200	-1.25000	-5.21200
H	-5.51900	0.02400	-6.17100
H	-6.54100	0.67100	-7.43700
H	-7.86000	1.95100	-5.82000
H	-7.15200	2.17300	-3.44200
H	-7.68500	0.52400	-3.77900
H	-5.96100	0.89500	-3.65900
H	-6.08600	3.60600	-5.21500
H	-4.84100	2.37900	-5.49700
H	-5.81800	2.97200	-6.84700
C	-7.34500	-4.57300	1.81300
C	-6.56000	-3.35100	1.33300
C	-7.40200	-2.12699	0.97900
S	-6.43700	-0.83100	0.10700
C	-6.13200	-1.70400	-1.45900
H	-8.12900	-4.84900	1.09900
H	-5.82700	-3.05800	2.09200
H	-5.97700	-3.64900	0.45900
H	-8.25200	-2.39600	0.34100
H	-7.80900	-1.63800	1.86900
H	-5.66200	-0.99300	-2.13600
H	-5.45400	-2.54999	-1.33800
H	-7.07400	-2.04300	-1.90300
H	1.24200	8.24799	-2.54400
H	0.18200	9.74200	1.43900
H	-1.82800	4.09300	6.39300
H	-2.35900	3.17200	7.82200
H	5.59800	0.58500	7.10800
H	4.02200	1.37200	7.01900
H	-0.45100	-3.18400	9.44699
H	-0.27600	-4.94000	9.23600
H	7.18500	-3.97600	6.14900
H	5.72300	-4.21200	7.08800
H	9.43500	-4.22400	-5.54800
H	7.65300	-4.38300	-5.54500
H	2.71800	-5.17800	-5.25200
H	2.29100	-0.57599	-7.55300
H	1.00000	-5.59600	-5.40500
H	0.85500	-1.41300	-8.18099
H	9.13299	0.31400	-7.34400
H	7.39000	0.52200	-7.28700
H	-3.46100	-6.14500	-5.55600
H	-5.09800	-5.50900	-5.76300
H	-3.17500	-8.08000	-0.03700
H	-1.81600	-8.89000	-0.85800
H	0.51800	-7.28800	3.35300
H	-3.68700	-4.61900	5.12399
H	-4.40299	-3.11000	5.76800
H	-6.26800	0.75200	5.02600
H	-6.91800	2.26900	4.38500
H	-11.04400	-0.58199	-3.27900
H	-10.56100	1.10100	-3.00500
H	5.82500	6.63300	-2.58900
H	5.08700	7.45200	-1.20800
H	-7.24700	-1.63200	-6.92100
H	-8.53600	-0.53400	-6.41000
H	-7.81500	-4.41500	2.78800
H	-6.65000	-5.41600	1.88800
H	-3.82500	8.04100	1.01099
H	-3.35700	7.12200	2.44499
H	-1.14300	-1.43300	2.19400

52A-TS-SS

O	1.82200	-0.89500	-1.74100
H	1.80900	-1.88000	-1.61500
H	1.41600	-0.81200	-2.63700

C	6.10600	-3.91400	6.10000	H	4.59300	5.81800	-1.63800
C	5.53500	-4.86900	5.03599	H	7.43600	6.59700	-0.74400
C	5.33800	-4.23700	3.64500	H	6.24600	5.90800	0.34500
C	5.24400	-5.31300	2.55799	H	7.22800	4.47400	-2.16299
C	4.08600	-3.34900	3.62300	H	8.66000	3.12200	-0.61500
H	5.81800	-2.87600	5.91000	H	9.13700	4.82800	-0.59500
H	6.19200	-5.74300	4.95200	H	8.18399	4.16099	0.73600
H	4.56200	-5.25700	5.36900	H	6.26000	2.45000	-0.99300
H	6.21500	-3.61100	3.42200	H	5.69500	3.53000	0.29900
H	5.07000	-4.87100	1.57300	H	5.01900	3.64800	-1.32900
H	6.16100	-5.91000	2.50900	C	0.47100	7.58400	-2.19000
H	4.41300	-5.99700	2.77200	C	0.01600	8.18600	-0.85500
H	3.97800	-2.85000	2.65600	O	-1.14900	8.47300	-0.60100
H	3.18600	-3.95000	3.78500	C	0.98500	6.15000	-1.98900
H	4.11599	-2.58000	4.40299	C	1.40600	5.41300	-3.26600
C	4.93400	1.13300	6.43600	C	2.13400	4.11800	-2.89900
C	4.62500	0.28000	5.20200	C	0.21000	5.11800	-4.17900
C	5.87300	-0.23600	4.48200	H	-0.37900	7.62700	-2.87000
S	6.99100	1.05200	3.80600	H	1.84400	6.16700	-1.30500
C	5.95400	1.66900	2.44300	H	0.20900	5.56200	-1.48100
H	5.42400	2.06900	6.15400	H	2.11000	6.06000	-3.81200
H	4.03200	-0.59400	5.50000	H	2.54599	3.63700	-3.78900
H	3.99400	0.84100	4.50100	H	2.96500	4.29800	-2.21300
H	6.50100	-0.80500	5.17500	H	1.46100	3.40600	-2.41000
H	5.60600	-0.91100	3.66300	H	0.52800	4.57700	-5.07900
H	6.53900	2.42800	1.92200	H	-0.52400	4.49300	-3.65600
H	5.71200	0.87300	1.73500	H	-0.29800	6.02900	-4.50699
H	5.03000	2.12500	2.80700	N	1.02200	8.38899	0.05100
C	8.24600	0.06400	-6.76200	C	0.74900	8.80100	1.42200
C	8.35800	0.56399	-5.30900	C	2.03300	8.84500	2.25300
C	7.02800	0.66700	-4.58800	C	2.59200	7.47600	2.59600
C	6.38900	-0.44900	-4.03500	C	3.87500	7.08400	2.19899
C	6.39200	1.90700	-4.45200	C	1.84000	6.58500	3.38100
C	5.17699	-0.34100	-3.35300	C	4.40900	5.85500	2.60200
C	5.17800	2.03600	-3.78200	C	2.36500	5.35600	3.77600
C	4.57200	0.91100	-3.21500	C	3.65900	4.99200	3.39700
O	3.39000	1.09500	-2.54199	H	1.94000	8.02000	-0.14900
H	8.07300	-1.01499	-6.80200	H	0.04900	8.08500	1.87600
H	9.03500	-0.09200	-4.75100	H	2.79000	9.44300	1.73200
H	8.82600	1.55600	-5.31000	H	1.80800	9.37800	3.18400
H	6.84200	-1.43200	-4.11700	H	4.47900	7.75800	1.59500
H	6.86200	2.79500	-4.86900	H	0.84200	6.86700	3.70100
H	4.72000	-1.21700	-2.90500	H	5.41800	5.58200	2.30799
H	4.71000	3.00700	-3.66600	H	1.77100	4.68400	4.38500
H	3.04700	0.24900	-2.15400	H	4.07599	4.04500	3.72300
C	1.33700	-1.02899	-7.27300	C	-4.07400	7.19800	1.63300
C	0.46500	-0.01100	-6.52600	C	-4.10200	5.89400	0.82900
C	0.99800	0.16600	-5.11300	C	-2.71000	5.61200	0.23700
N	1.52200	1.36100	-4.77700	C	-5.19500	5.94000	-0.24600
O	0.95100	-0.77800	-4.31200	C	-2.61200	4.30100	-0.54000
H	1.53000	-1.88600	-6.62100	H	-5.04900	7.41500	2.08400
H	0.42100	0.94400	-7.06000	H	-4.34900	5.07300	1.51700
H	-0.55600	-0.39100	-6.43800	H	-2.42000	6.45400	-0.40300
H	2.07399	1.42300	-3.91800	H	-1.98400	5.60500	1.06300
H	1.65200	2.07300	-5.47700	H	-5.30200	4.97900	-0.76200
C	5.46100	6.48100	-1.58600	H	-6.16500	6.18700	0.19500
C	6.57500	5.91800	-0.70100	H	-4.96699	6.70400	-1.00000
C	7.03100	4.49700	-1.08100	H	-1.59300	4.10400	-0.88700
C	8.32799	4.13100	-0.35200	H	-2.92400	3.44500	0.06900
C	5.94000	3.47100	-0.76800	H	-3.25000	4.30999	-1.42700

C	-1.98500	3.10700	6.78800
C	-0.74100	2.24700	6.50600
C	-0.26900	2.44800	5.05000
C	-0.99200	0.77500	6.84500
C	1.02299	1.71600	4.67699
H	-2.86800	2.70200	6.28300
H	0.06900	2.60800	7.15800
H	-1.06700	2.14200	4.36500
H	-0.13200	3.52600	4.88600
H	-0.09100	0.17100	6.72200
H	-1.32800	0.66200	7.88100
H	-1.76900	0.35300	6.19600
H	1.35400	1.99800	3.67100
H	1.83200	1.95600	5.37700
H	0.89100	0.63000	4.68200
C	-0.29800	-3.96100	8.71700
C	0.97900	-3.76300	7.88700
C	1.17200	-4.92199	6.90200
C	0.95600	-2.42600	7.14000
H	-1.18100	-3.96900	8.06700
H	1.83300	-3.75100	8.57900
H	2.09700	-4.79200	6.32600
H	1.23000	-5.88300	7.42500
H	0.33800	-4.95899	6.19300
H	1.88000	-2.27199	6.56900
H	0.12300	-2.39699	6.42800
H	0.84300	-1.58600	7.83100
C	1.38600	-6.62500	3.54900
N	1.18100	-5.31200	2.97500
C	1.38200	-5.01700	1.68200
N	2.06800	-5.82500	0.86500
N	0.91100	-3.85500	1.21900
H	1.49900	-6.51200	4.62700
H	2.29500	-7.10300	3.17900
H	0.41600	-4.79100	3.45600
H	2.49700	-6.65500	1.23400
H	2.22100	-5.60200	-0.13700
H	0.20800	-3.38500	1.80400
H	0.98600	-3.69100	0.20500
C	-3.46300	-3.65900	5.64500
C	-2.64200	-2.73500	4.70500
C	-1.44400	-3.31200	3.95600
O	-0.98700	-4.41800	4.31200
O	-0.94000	-2.61400	2.99600
H	-2.97300	-3.83200	6.60300
H	-3.30400	-2.30500	3.94700
H	-2.27000	-1.87200	5.27200
C	-6.00600	1.65800	4.43600
C	-5.60100	1.21700	3.02400
C	-4.47300	0.17000	3.08000
C	-5.24300	2.41200	2.13400
C	-3.12800	0.71200	3.56200
H	-5.20200	2.20100	4.94400
H	-6.47400	0.72900	2.57300
H	-4.79399	-0.64700	3.74000
H	-4.35300	-0.27700	2.08800
H	-4.93800	2.08500	1.13500
H	-6.09900	3.08100	2.01500
H	-4.42000	2.99500	2.56100
H	-2.38200	-0.07700	3.64000
H	-2.72800	1.45900	2.86900
H	-3.22100	1.18500	4.54399
C	-2.36100	-7.93900	-0.74300
C	-1.34000	-6.87000	-0.29700
C	-1.97500	-5.53100	-0.03100
C	-2.59800	-5.27800	1.19700
C	-2.01799	-4.54500	-1.02200
C	-3.27300	-4.08200	1.41400
C	-2.70200	-3.34700	-0.81200
C	-3.33500	-3.11500	0.41000
H	-2.81800	-7.68400	-1.70300
H	-0.83700	-7.22200	0.61200
H	-0.55700	-6.76800	-1.05900
H	-2.54300	-6.01700	1.99100
H	-1.51600	-4.71500	-1.96700
H	-3.75600	-3.90500	2.36800
H	-2.74200	-2.60300	-1.60200
H	-3.88400	-2.19600	0.57799
C	8.57100	-4.55600	-4.96500
C	8.48800	-3.75900	-3.65300
C	7.17300	-3.88600	-2.92800
C	6.13500	-4.75200	-3.19900
C	6.73200	-3.07800	-1.81400
C	5.41100	-3.50200	-1.48100
C	7.30700	-2.02199	-1.08600
N	5.08000	-4.52900	-2.33599
C	4.66099	-2.88800	-0.46900
C	6.57400	-1.41800	-0.07099
C	5.26200	-1.84300	0.22700
H	8.64600	-5.63200	-4.77200
H	9.31500	-4.05100	-2.99000
H	8.66200	-2.69600	-3.87200
H	6.06300	-5.52100	-3.95300
H	4.20000	-5.04200	-2.30100
H	8.31000	-1.67600	-1.31800
H	3.64700	-3.20800	-0.25400
H	7.01800	-0.61100	0.50200
H	4.71300	-1.35300	1.02699
C	1.80500	-5.38600	-4.68200
C	1.40500	-4.23900	-3.73800
C	1.76600	-4.40400	-2.25999
O	2.37699	-5.43700	-1.87100
O	1.38100	-3.47100	-1.48100
H	1.98800	-6.29300	-4.10100
H	1.79900	-3.27200	-4.06799
H	0.32200	-4.10100	-3.72600
C	-4.42600	-6.00900	-5.04399
C	-4.27200	-5.16099	-3.78000
C	-3.66400	-3.81500	-4.08100
C	-4.43800	-2.65200	-4.09700
C	-2.29599	-3.70700	-4.36000
C	-3.84900	-1.40500	-4.31600
C	-1.70200	-2.46900	-4.58399
C	-2.48000	-1.31200	-4.54699
H	-4.84199	-6.99600	-4.81900
H	-5.24900	-5.02400	-3.30400
H	-3.64400	-5.68400	-3.05000
H	-5.50500	-2.72200	-3.91100
H	-1.68800	-4.60799	-4.37500
H	-4.45300	-0.50800	-4.28200

H	-0.63500	-2.38499	-4.74800
H	-2.01600	-0.34100	-4.67100
C	-10.88100	0.19700	-2.50000
C	-9.89600	-0.30300	-1.43200
C	-9.60900	0.69200	-0.33000
C	-9.93600	0.41000	0.99900
C	-8.96700	1.90400	-0.61300
C	-9.60800	1.29800	2.02500
C	-8.64000	2.79700	0.40400
C	-8.95600	2.49500	1.73000
H	-11.85000	0.44100	-2.05600
H	-10.28200	-1.22800	-0.98800
H	-8.94600	-0.56499	-1.91400
H	-10.43500	-0.52600	1.23600
H	-8.69999	2.13800	-1.63900
H	-9.86100	1.05600	3.05300
H	-8.12700	3.72500	0.16500
H	-8.69600	3.18600	2.52500
C	-0.84800	-0.77100	0.08400
O	-1.18000	-0.63300	1.48000
C	0.95400	1.00000	0.68500
C	2.34800	1.21700	0.71800
C	0.07600	2.10500	0.78000
C	2.84700	2.50999	0.77200
C	0.59000	3.38900	0.88900
C	1.97400	3.59600	0.86000
C	-1.66100	0.11600	-0.82800
C	-1.05900	0.73100	-1.93200
C	-3.02300	0.31600	-0.59200
C	-1.81200	1.53000	-2.78900
C	-3.77900	1.10700	-1.45900
C	-3.17700	1.71300	-2.56199
C	0.50300	-0.34500	0.49100
H	3.02200	0.37100	0.63000
H	-0.99100	1.94000	0.80800
H	3.91400	2.67400	0.74600
H	-0.08300	4.23200	0.99500
H	2.37000	4.60200	0.92300
H	-0.00300	0.59200	-2.11100
H	-3.49300	-0.12800	0.27400
H	-1.32400	2.00900	-3.63300
H	-4.83200	1.26600	-1.25499
H	-3.76400	2.33900	-3.22500
H	-0.87300	-1.81100	-0.24500
H	1.18000	-1.14900	0.76600
C	-7.49200	-0.82400	-6.21300
C	-6.54100	0.36800	-6.33900
C	-6.87600	1.57400	-5.44000
C	-6.94200	1.21600	-3.95000
C	-5.86300	2.70100	-5.67300
H	-7.45100	-1.27099	-5.21400
H	-5.51800	0.03599	-6.12400
H	-6.53300	0.71200	-7.38100
H	-7.87000	1.94200	-5.73700
H	-7.16100	2.10300	-3.34800
H	-7.71400	0.47300	-3.73500
H	-5.98400	0.81600	-3.59800
H	-6.10200	3.58600	-5.07400
H	-4.85300	2.37200	-5.39500
H	-5.83300	3.00300	-6.72500
C	-7.33600	-4.55600	1.82800
C	-6.57600	-3.33200	1.31300
C	-7.44900	-2.14299	0.92400
S	-6.50200	-0.81700	0.08000
C	-6.10400	-1.66700	-1.47600
H	-8.13299	-4.84800	1.13399
H	-5.85200	-2.99900	2.06400
H	-5.98600	-3.64100	0.44800
H	-8.26900	-2.44800	0.26300
H	-7.90100	-1.66400	1.79700
H	-5.53900	-0.96100	-2.08199
H	-5.47600	-2.54500	-1.31800
H	-7.01400	-1.95200	-2.01100
H	1.26099	8.24900	-2.55600
H	0.20600	9.75100	1.42600
H	-1.80700	4.11200	6.39000
H	-2.20400	3.17500	7.85800
H	5.61600	0.59800	7.10300
H	4.02299	1.37100	6.99200
H	-0.42700	-3.16300	9.45600
H	-0.26100	-4.91899	9.24499
H	7.19800	-3.96700	6.15100
H	5.73900	-4.17000	7.10000
H	9.43500	-4.25699	-5.56500
H	7.65600	-4.39300	-5.54200
H	2.71700	-5.15700	-5.24200
H	2.30300	-0.59700	-7.55100
H	1.00200	-5.59800	-5.39600
H	0.85900	-1.42000	-8.17700
H	9.13899	0.30000	-7.35000
H	7.40000	0.54900	-7.26200
H	-3.46200	-6.15100	-5.54400
H	-5.09700	-5.50600	-5.74900
H	-3.15600	-8.06199	-0.00200
H	-1.81400	-8.88299	-0.84100
H	0.52400	-7.27600	3.36500
H	-3.68300	-4.62000	5.17600
H	-4.38900	-3.09000	5.77900
H	-6.25200	0.77300	5.03200
H	-6.88400	2.31300	4.41300
H	-11.03600	-0.56899	-3.26700
H	-10.50400	1.09800	-2.99400
H	5.84200	6.63100	-2.60200
H	5.11599	7.45400	-1.21900
H	-7.23200	-1.60800	-6.93000
H	-8.52900	-0.52800	-6.40100
H	-7.79100	-4.38500	2.80900
H	-6.64200	-5.40000	1.90300
H	-3.79300	8.04200	0.99400
H	-3.33600	7.13600	2.43900
H	-1.16900	-1.50100	2.06900

60A-React

O	1.15500	-1.16900	-2.18800
H	0.58800	-1.67100	-2.81800
H	1.82100	-1.84100	-1.91700
C	6.97400	1.93600	4.20099
C	6.89600	0.40800	4.13900

C	7.16000	-0.23000	2.76300
C	7.21700	-1.75800	2.89900
C	6.10600	0.15900	1.72000
H	6.24100	2.40400	3.53500
H	7.60600	-0.01500	4.86300
H	5.89800	0.08900	4.47400
H	8.14000	0.12200	2.40899
H	7.39900	-2.23900	1.93300
H	8.00500	-2.07200	3.59300
H	6.26100	-2.13700	3.28000
H	6.32500	-0.28199	0.74400
H	5.11700	-0.20700	2.02300
H	6.03800	1.24100	1.58100
C	4.60200	0.79200	-9.07100
C	4.52400	1.90400	-8.01700
C	3.57600	1.60300	-6.86900
C	3.66800	0.43000	-6.10600
C	2.57100	2.51600	-6.52400
C	2.83200	0.19600	-5.01499
C	1.72100	2.29300	-5.44400
C	1.87200	1.14700	-4.66000
O	1.02499	0.98400	-3.59700
H	5.10600	-0.09500	-8.67400
H	5.53100	2.09900	-7.62300
H	4.20400	2.83700	-8.49300
H	4.41800	-0.31500	-6.34600
H	2.45500	3.42600	-7.11000
H	2.94400	-0.70200	-4.41800
H	0.94700	3.00600	-5.18600
H	1.30100	0.21400	-3.01400
C	-0.42200	-3.61100	-6.76600
C	-1.47400	-2.74400	-6.06200
C	-0.81100	-1.90000	-5.00000
N	-0.83800	-0.56399	-5.15900
O	-0.26500	-2.45800	-4.03599
H	0.15500	-4.17000	-6.02700
H	-2.02300	-2.13000	-6.78400
H	-2.19600	-3.38800	-5.55000
H	-0.28999	0.04200	-4.54200
H	-1.20800	-0.16400	-6.00500
C	-0.34600	6.52600	-4.76900
C	1.04200	6.82800	-4.18400
C	1.97200	5.60400	-4.09600
C	3.43600	6.02400	-3.92700
C	1.56000	4.65400	-2.96500
H	-0.75900	5.58600	-4.38900
H	1.51900	7.60500	-4.79500
H	0.94100	7.26400	-3.18100
H	1.89500	5.05800	-5.04699
H	4.09100	5.14799	-3.89900
H	3.76600	6.66700	-4.74899
H	3.57800	6.57900	-2.99100
H	2.14100	3.73000	-2.98700
H	1.71500	5.13100	-1.98800
H	0.50700	4.36900	-3.02700
C	-5.06400	4.96800	-3.58500
C	-5.54000	5.67400	-2.32399
O	-6.49300	5.27900	-1.65500
C	-3.78600	4.14499	-3.32900
C	-3.28100	3.35100	-4.54500
C	-1.94200	2.68200	-4.21600
C	-4.29000	2.29599	-5.01900
H	-5.88700	4.33700	-3.92300
H	-2.99000	4.82599	-3.00400
H	-3.94900	3.46100	-2.49200
H	-3.11700	4.06400	-5.36700
H	-1.52100	2.18900	-5.09700
H	-1.20200	3.40600	-3.86500
H	-2.05600	1.93200	-3.42800
H	-3.89400	1.74300	-5.87800
H	-4.48900	1.57200	-4.22100
H	-5.24300	2.73400	-5.32400
N	-4.81900	6.77400	-1.96100
C	-5.11700	7.53600	-0.75700
C	-4.00600	8.54800	-0.45600
C	-2.70700	7.90700	-0.01900
C	-1.65200	7.69900	-0.91500
C	-2.54000	7.49900	1.31200
C	-0.45700	7.11600	-0.49000
C	-1.34800	6.92000	1.74100
C	-0.29900	6.73300	0.84100
H	-4.02900	7.05000	-2.52400
H	-5.22499	6.83700	0.07900
H	-3.83900	9.17700	-1.34000
H	-4.36800	9.21400	0.33400
H	-1.75000	8.02100	-1.94900
H	-3.35200	7.65200	2.01900
H	0.35300	6.96900	-1.19500
H	-1.23400	6.62300	2.77900
H	0.63900	6.30000	1.17600
C	-8.41799	4.48900	1.89600
C	-8.20600	3.02500	1.50500
C	-6.99400	2.87500	0.59100
S	-6.92800	1.22600	-0.21500
C	-5.73100	1.64700	-1.52500
H	-9.24900	4.59400	2.60100
H	-8.08799	2.40400	2.39800
H	-9.10000	2.66200	0.98500
H	-7.02700	3.62400	-0.20400
H	-6.07200	3.03400	1.15900
H	-5.75000	0.84800	-2.26300
H	-6.03100	2.58700	-1.99100
H	-4.71800	1.72900	-1.12400
C	-2.72600	3.86100	5.28200
C	-1.67700	3.87400	4.16899
C	-0.71200	2.73000	4.18600
C	-0.67500	1.64600	5.02400
C	0.40800	2.58800	3.28600
C	1.09600	1.39700	3.64200
C	0.88100	3.36400	2.21900
N	0.38700	0.81100	4.67800
C	2.26399	0.99300	2.98700
C	2.03900	2.96600	1.56400
C	2.72800	1.79800	1.95100
H	-3.26200	2.90500	5.30600
H	-2.17700	3.90000	3.19400
H	-1.10500	4.81100	4.22600
H	-1.32300	1.38400	5.84700
H	0.78200	0.12800	5.30300
H	0.33300	4.24099	1.89700

H	2.78600	0.08700	3.26700
H	2.42000	3.55700	0.73600
H	3.63200	1.51600	1.42500
C	2.73500	-1.07700	8.38700
C	3.83900	-0.57399	7.44500
C	4.64700	-1.75600	6.89300
C	3.26800	0.25500	6.29400
H	1.99800	-1.67600	7.83700
H	4.51700	0.06200	8.03100
H	5.44200	-1.41900	6.22000
H	5.10600	-2.34100	7.69600
H	3.98300	-2.42099	6.32600
H	4.05500	0.72800	5.69900
H	2.71100	-0.40600	5.62200
H	2.60000	1.04600	6.64800
C	4.79900	-4.56799	4.08700
N	4.03200	-3.71300	3.19300
C	4.03899	-3.71800	1.84600
N	4.81200	-4.55800	1.13700
N	3.30200	-2.82500	1.18500
H	4.93400	-4.03700	5.02900
H	5.79400	-4.75900	3.67300
H	3.30200	-3.14600	3.63200
H	5.21100	-5.36400	1.58800
H	4.70899	-4.58200	0.10700
H	2.62800	-2.27400	1.70600
H	3.09200	-2.99100	0.17100
C	-5.63400	0.10600	5.82700
C	-5.47400	-0.36700	4.37899
C	-6.03100	0.66800	3.39900
C	-4.00300	-0.67700	4.06799
H	-5.12300	1.06600	5.97200
H	-6.04500	-1.29800	4.25300
H	-5.94700	0.34200	2.35900
H	-7.08800	0.86700	3.60200
H	-5.49300	1.61900	3.49700
H	-3.87700	-1.02499	3.03700
H	-3.37600	0.21600	4.19100
H	-3.61100	-1.45500	4.73400
C	-4.03500	-6.88700	4.63199
C	-4.15900	-5.60700	3.79800
C	-3.71900	-5.79000	2.33599
C	-4.58900	-6.80500	1.58700
C	-3.69200	-4.45300	1.59300
H	-4.77600	-7.63700	4.34100
H	-5.19299	-5.23299	3.82300
H	-3.54100	-4.82100	4.25300
H	-2.69200	-6.18100	2.35200
H	-4.24200	-6.91700	0.55700
H	-4.56799	-7.79200	2.05799
H	-5.63400	-6.47200	1.55300
H	-3.31400	-4.56300	0.57299
H	-4.69900	-4.02400	1.52500
H	-3.05900	-3.72400	2.11300
C	7.55800	-1.58900	-6.12300
C	7.11800	-0.39200	-5.27900
C	6.32200	-0.75300	-4.05900
C	6.09000	-1.99900	-3.52700
C	5.59100	0.17600	-3.23400
C	4.92300	-0.58199	-2.23000

C	5.39700	1.56500	-3.28900
N	5.26800	-1.90400	-2.42000
C	4.04399	0.01500	-1.31900
C	4.52600	2.15899	-2.38600
C	3.84600	1.38700	-1.42200
H	8.26800	-2.21400	-5.57000
H	8.00700	0.18700	-4.99099
H	6.51900	0.29099	-5.89700
H	6.43800	-2.96900	-3.85100
H	4.92000	-2.70700	-1.90900
H	5.89200	2.16000	-4.05100
H	3.52200	-0.57399	-0.57399
H	4.35600	3.22900	-2.42400
H	3.13800	1.87000	-0.75800
C	2.99400	-5.92600	-4.03099
C	2.29599	-4.91399	-3.11000
C	3.14200	-4.25699	-2.00999
O	4.28800	-4.69000	-1.72300
O	2.57600	-3.29900	-1.37500
H	3.76200	-6.46100	-3.46500
H	1.80300	-4.11400	-3.67400
H	1.48900	-5.40400	-2.55600
C	-1.88100	-9.93000	-2.75500
C	-1.92100	-8.93300	-1.58600
C	-2.19700	-7.50000	-1.98800
C	-3.49500	-6.98100	-1.93900
C	-1.16100	-6.64600	-2.39600
C	-3.74900	-5.64000	-2.23700
C	-1.40800	-5.30800	-2.71000
C	-2.70300	-4.79699	-2.61600
H	-1.71000	-10.95000	-2.39500
H	-2.68400	-9.25200	-0.86600
H	-0.96300	-8.97600	-1.05600
H	-4.31600	-7.63000	-1.64400
H	-0.14700	-7.03400	-2.45400
H	-4.75800	-5.25400	-2.15600
H	-0.60500	-4.65000	-3.01800
H	-2.88900	-3.74800	-2.82200
C	-7.28300	-6.98500	-2.92200
C	-7.25800	-5.81500	-3.90900
C	-7.97500	-4.54300	-3.42400
C	-7.36700	-3.99000	-2.13200
C	-7.96100	-3.47100	-4.51999
H	-6.71800	-6.75900	-2.01200
H	-6.21400	-5.56700	-4.14400
H	-7.71500	-6.13300	-4.85500
H	-9.02300	-4.80999	-3.22100
H	-7.89600	-3.08900	-1.80300
H	-7.40600	-4.71200	-1.31300
H	-6.31800	-3.71900	-2.28399
H	-8.49900	-2.57100	-4.20400
H	-6.93000	-3.17600	-4.75600
H	-8.42200	-3.83400	-5.44400
C	-9.61900	-5.95300	1.96700
C	-8.52500	-4.94600	1.60000
C	-8.62400	-3.58300	2.30900
C	-8.37299	-3.69700	3.81800
C	-7.65200	-2.57100	1.68500
H	-9.61700	-6.19500	3.03500
H	-7.53600	-5.37900	1.81100

H	-8.55599	-4.77600	0.51800
H	-9.64500	-3.20400	2.16100
H	-8.45400	-2.71900	4.30500
H	-9.08400	-4.37000	4.30700
H	-7.36300	-4.08000	4.01199
H	-7.74000	-1.58600	2.15200
H	-6.61400	-2.90400	1.81100
H	-7.83700	-2.44700	0.61400
C	-1.04000	-1.08500	-0.02500
O	-1.12700	-0.82200	1.41100
C	-1.29000	1.46700	0.37100
C	-0.79300	2.41699	-0.53200
C	-2.45000	1.73800	1.09800
C	-1.44500	3.63700	-0.68400
C	-3.10000	2.96300	0.93800
C	-2.59600	3.91900	0.05400
C	-2.32300	-1.18000	-0.77300
C	-2.31100	-0.87700	-2.13700
C	-3.51100	-1.58800	-0.16600
C	-3.46700	-1.03099	-2.89900
C	-4.67000	-1.74400	-0.92800
C	-4.64499	-1.48000	-2.29700
C	-0.54600	0.19100	0.52300
H	0.09800	2.19700	-1.11300
H	-2.82800	0.99800	1.79200
H	-1.05000	4.37800	-1.36700
H	-3.99600	3.17200	1.51200
H	-3.09100	4.87700	-0.06100
H	-1.39600	-0.52700	-2.59100
H	-3.52400	-1.79000	0.89700
H	-3.44300	-0.79200	-3.95600
H	-5.58800	-2.06300	-0.44900
H	-5.54600	-1.60500	-2.88900
H	-0.27700	-1.81400	-0.29199
H	0.53100	0.28999	0.63800
C	1.07900	-8.60900	1.38900
C	1.37800	-7.11700	1.20700
C	0.07400	-6.30100	1.17800
C	2.23400	-6.88800	-0.04000
C	0.27500	-4.78400	1.14799
H	0.40300	-8.97100	0.60700
H	1.96100	-6.77900	2.08000
H	-0.52300	-6.60600	0.31000
H	-0.51700	-6.56700	2.06500
H	2.49800	-5.83900	-0.16400
H	3.16800	-7.45900	0.00600
H	1.70000	-7.20900	-0.94000
H	-0.68500	-4.26300	1.21600
H	0.89800	-4.45899	1.98700
H	0.76600	-4.44700	0.23000
C	3.22600	5.94900	4.08200
C	2.16299	6.37000	5.07500
H	4.21500	6.00200	4.55000
H	2.15800	5.71800	5.95500
H	3.24000	6.60500	3.20300
H	3.06800	4.92600	3.72800
C	-0.62800	-3.10500	5.76700
C	-0.61000	-2.50000	4.36500
C	0.78700	-2.30500	3.82500
O	1.79600	-2.57700	4.47100
O	0.90400	-1.81400	2.60000
H	0.14000	-2.65100	6.39700
H	-1.16500	-3.11800	3.64800
H	-1.09900	-1.51900	4.34500
H	2.31999	7.39700	5.42600
H	1.15999	6.32500	4.63500
H	-6.08400	8.04200	-0.85400
H	-4.84900	5.70800	-4.36300
H	-8.65499	5.08600	1.00800
H	-7.53700	4.95000	2.35500
H	-5.18300	-0.61000	6.52500
H	-6.67600	0.23900	6.13600
H	-9.48800	-6.89100	1.41700
H	-10.60800	-5.55500	1.71500
H	-6.83600	-7.88000	-3.36600
H	-8.30300	-7.23800	-2.61400
H	-1.07700	-9.67600	-3.45400
H	-2.82000	-9.91499	-3.31900
H	0.26600	-3.00500	-7.36200
H	-0.93200	-4.32400	-7.42200
H	3.49000	-5.43100	-4.87100
H	2.29199	-6.66700	-4.42900
H	0.61000	-8.80599	2.35900
H	1.99600	-9.20700	1.33800
H	7.96200	2.32100	3.92900
H	6.75700	2.28900	5.21500
H	-3.46800	4.64300	5.08700
H	-2.28200	4.02700	6.26700
H	-4.16000	-6.69100	5.70100
H	-3.04300	-7.32000	4.47100
H	8.05599	-1.26000	-7.04000
H	6.72000	-2.23500	-6.40500
H	5.12500	1.08800	-9.98600
H	3.59200	0.48300	-9.36600
H	2.21099	-0.25600	8.88599
H	3.19400	-1.72200	9.14500
H	-0.46900	-4.18800	5.77300
H	-1.60300	-2.92700	6.23300
H	-0.31000	6.46900	-5.86200
H	-1.06000	7.32000	-4.52100
H	0.03200	-1.48900	2.18699
H	4.30199	-5.52100	4.29399

60A-TS-RR

O	0.81800	-1.54800	-1.78700
H	0.17600	-1.47100	-2.54100
H	0.80300	-2.52000	-1.58200
C	6.38800	-3.55100	5.42900
C	5.31000	-4.61500	5.19600
C	5.27800	-5.25600	3.79600
C	4.25900	-6.40300	3.77200
C	4.94900	-4.24700	2.69000
H	6.26400	-2.70100	4.75100
H	5.42900	-5.41300	5.94200
H	4.32500	-4.16800	5.38600
H	6.27500	-5.67600	3.59300
H	4.21699	-6.88100	2.78700
H	4.49899	-7.17200	4.51499

H	3.25600	-6.01900	3.99400
H	4.95500	-4.71900	1.70300
H	3.94600	-3.83000	2.84000
H	5.66000	-3.41800	2.65900
C	5.82400	-2.50700	-8.05000
C	6.37000	-1.69000	-6.87100
C	5.30900	-1.25899	-5.87200
C	4.45700	-2.17400	-5.23500
C	5.16200	0.09200	-5.53700
C	3.52900	-1.77000	-4.27600
C	4.23600	0.51600	-4.58700
C	3.43500	-0.42000	-3.92800
O	2.55500	0.04800	-2.98500
H	5.51400	-3.50700	-7.72800
H	7.14700	-2.27199	-6.35700
H	6.86900	-0.79100	-7.25000
H	4.52500	-3.23100	-5.46800
H	5.79400	0.83200	-6.02300
H	2.91000	-2.50199	-3.76900
H	4.14300	1.56300	-4.33100
H	2.12699	-0.69000	-2.47500
C	-1.11800	-2.35700	-6.73600
C	-1.34200	-0.95200	-6.15100
C	-0.54900	-0.74000	-4.87899
N	0.36800	0.24200	-4.87200
O	-0.77100	-1.46300	-3.89300
H	-1.25299	-3.11400	-5.96100
H	-1.11400	-0.18100	-6.89400
H	-2.39699	-0.84500	-5.87900
H	1.02800	0.33100	-4.09600
H	0.58399	0.73800	-5.72200
C	5.45500	4.97300	-3.60200
C	6.66100	4.34199	-2.89300
C	6.69800	2.80400	-2.95600
C	8.09200	2.26900	-2.60900
C	5.64700	2.17600	-2.03600
H	4.51700	4.46699	-3.35700
H	7.57900	4.74700	-3.33800
H	6.68400	4.64700	-1.83900
H	6.47000	2.50199	-3.98900
H	8.11700	1.17600	-2.66200
H	8.85399	2.66000	-3.29300
H	8.37700	2.56000	-1.59100
H	5.61300	1.09100	-2.14800
H	5.87200	2.40400	-0.98600
H	4.64100	2.54500	-2.24900
C	0.80900	7.02000	-3.04700
C	0.76300	7.91900	-1.81600
O	-0.27000	8.46100	-1.42100
C	1.11100	5.54300	-2.74800
C	1.35200	4.71000	-4.01900
C	1.71900	3.26700	-3.66700
C	0.15300	4.73500	-4.97600
H	-0.15500	7.13000	-3.54400
H	2.00199	5.46400	-2.11300
H	0.28799	5.11700	-2.15899
H	2.21000	5.16000	-4.54200
H	2.05799	2.72100	-4.55300
H	2.51200	3.21200	-2.91800
H	0.85800	2.73100	-3.26000

H	0.33800	4.09700	-5.84700
H	-0.74800	4.35799	-4.47800
H	-0.06600	5.74000	-5.34600
N	1.96000	8.12400	-1.20000
C	2.08500	8.88100	0.03800
C	3.53600	8.86800	0.53300
C	4.00500	7.50700	0.99900
C	4.83000	6.70100	0.20400
C	3.61500	7.02300	2.25700
C	5.26900	5.45600	0.66200
C	4.05500	5.78500	2.71800
C	4.89100	5.00000	1.92300
H	2.74400	7.54800	-1.47200
H	1.43300	8.43900	0.80300
H	4.19200	9.24200	-0.26300
H	3.61000	9.58100	1.36100
H	5.16099	7.06200	-0.76700
H	2.97300	7.63500	2.88500
H	5.91500	4.84800	0.04000
H	3.75000	5.43300	3.69800
H	5.24700	4.04100	2.28399
C	-2.74600	8.84300	2.00900
C	-3.22400	7.60000	1.23800
C	-2.02000	6.79100	0.74900
S	-2.38800	5.36800	-0.34900
C	-2.90200	6.27600	-1.85200
H	-3.51300	9.24400	2.67700
H	-3.85000	6.96600	1.87400
H	-3.84600	7.90200	0.38800
H	-1.31300	7.43700	0.22300
H	-1.50100	6.35100	1.60800
H	-2.88100	5.56800	-2.68400
H	-3.91400	6.67600	-1.75800
H	-2.19700	7.08900	-2.04300
C	0.47600	4.44000	5.81600
C	1.45900	3.76000	4.85900
C	1.38300	2.26399	4.81700
C	0.48800	1.43100	5.44100
C	2.27400	1.41400	4.06600
C	1.86200	0.06900	4.27700
C	3.37700	1.67000	3.24000
N	0.75400	0.11100	5.09999
C	2.53399	-1.01400	3.70200
C	4.05300	0.59800	2.67100
C	3.63900	-0.72900	2.90600
H	-0.54900	4.09800	5.63400
H	1.30700	4.14600	3.84300
H	2.48400	4.05300	5.13100
H	-0.33100	1.66500	6.10400
H	0.36400	-0.69200	5.56500
H	3.67500	2.69000	3.02800
H	2.19899	-2.03200	3.85900
H	4.90800	0.78000	2.02700
H	4.18700	-1.54500	2.45100
C	0.67500	-2.95600	8.83400
C	1.93800	-3.32100	8.03999
C	1.79400	-4.72000	7.42500
C	2.22900	-2.29500	6.94300
H	-0.18900	-2.87200	8.16400
H	2.78500	-3.33600	8.74100

H	2.68700	-5.00200	6.85800
H	1.62600	-5.48100	8.19500
H	0.93800	-4.72800	6.74000
H	3.19800	-2.46900	6.46700
H	1.46500	-2.38200	6.16500
H	2.22700	-1.26899	7.32600
C	0.45600	-6.83100	4.37500
N	0.59200	-5.65200	3.53100
C	0.76600	-5.62500	2.19800
N	0.90400	-6.74700	1.46600
N	0.86500	-4.44700	1.58000
H	0.77400	-6.56100	5.38200
H	1.11100	-7.63600	4.02900
H	0.34700	-4.75399	3.98100
H	0.66300	-7.63700	1.86600
H	0.93600	-6.66100	0.44000
H	0.58500	-3.60900	2.10300
H	0.78000	-4.40299	0.54900
C	-4.23800	3.65900	5.65900
C	-4.19400	3.30200	4.16600
C	-3.80000	4.51600	3.31800
C	-3.24300	2.12500	3.90700
H	-3.25000	3.99200	6.00200
H	-5.20400	2.99000	3.86100
H	-3.79600	4.28099	2.24900
H	-4.49200	5.35100	3.47800
H	-2.79400	4.86400	3.58600
H	-3.23200	1.84300	2.84900
H	-2.21300	2.37900	4.18900
H	-3.53500	1.24100	4.48500
C	-7.60000	-2.52400	3.83000
C	-6.88400	-1.52400	2.91600
C	-6.66200	-2.04000	1.48300
C	-7.97600	-2.36100	0.76000
C	-5.83100	-1.05100	0.66300
H	-8.66000	-2.61900	3.58000
H	-7.44300	-0.57799	2.87900
H	-5.90300	-1.27899	3.34600
H	-6.08800	-2.97400	1.56100
H	-7.77800	-2.71800	-0.25600
H	-8.55300	-3.13300	1.27800
H	-8.60600	-1.46700	0.68200
H	-5.57200	-1.47200	-0.31400
H	-6.39100	-0.12400	0.48800
H	-4.90400	-0.78100	1.18000
C	5.94400	-6.32300	-5.13000
C	6.29000	-5.15900	-4.19700
C	5.27300	-4.90700	-3.12200
C	4.18499	-5.67900	-2.78500
C	5.22900	-3.75700	-2.25300
C	4.07400	-3.89300	-1.42800
C	6.02900	-2.61000	-2.12699
N	3.47200	-5.08600	-1.76200
C	3.69200	-2.89900	-0.51900
C	5.65700	-1.62700	-1.22000
C	4.49300	-1.76500	-0.43400
H	5.96200	-7.27400	-4.58700
H	7.27600	-5.34200	-3.74700
H	6.41200	-4.24099	-4.78800
H	3.84700	-6.61900	-3.19800
H	2.60600	-5.46300	-1.38800
H	6.91100	-2.48400	-2.74700
H	2.80600	-3.01400	0.09500
H	6.26800	-0.73700	-1.11000
H	4.21900	-0.97200	0.25200
C	-0.58900	-6.43200	-3.97600
C	-0.55300	-5.18100	-3.07800
C	0.32100	-5.23100	-1.81600
O	0.81800	-6.32200	-1.43000
O	0.44900	-4.13300	-1.17000
H	-0.49500	-7.32800	-3.35700
H	-0.27800	-4.28000	-3.63800
H	-1.55600	-4.97700	-2.68900
C	-7.00500	-6.07100	-3.62200
C	-6.51100	-5.31600	-2.37699
C	-5.69800	-4.09100	-2.72900
C	-6.31100	-2.83900	-2.86000
C	-4.32200	-4.18499	-2.97300
C	-5.56900	-1.71200	-3.21500
C	-3.57300	-3.06300	-3.32800
C	-4.19800	-1.82200	-3.45000
H	-7.62400	-6.93000	-3.34500
H	-7.37000	-5.01999	-1.76400
H	-5.90700	-5.99100	-1.76200
H	-7.37900	-2.74500	-2.67900
H	-3.83300	-5.15100	-2.87800
H	-6.06000	-0.75000	-3.29400
H	-2.50599	-3.13000	-3.49700
H	-3.60600	-0.94800	-3.69700
C	-8.92700	-0.23300	-3.95400
C	-8.07600	0.57399	-4.93800
C	-7.74800	2.00500	-4.47499
C	-6.85400	2.02300	-3.22900
C	-7.09000	2.80100	-5.60900
H	-8.43300	-0.36100	-2.98600
H	-7.13700	0.04200	-5.14200
H	-8.60500	0.63100	-5.89700
H	-8.69800	2.49800	-4.21900
H	-6.65700	3.05000	-2.90200
H	-7.30200	1.49200	-2.38400
H	-5.88700	1.55500	-3.44700
H	-6.87400	3.83000	-5.30200
H	-6.14200	2.33700	-5.90600
H	-7.73300	2.84000	-6.49400
C	-10.64200	2.01399	0.78300
C	-9.13599	1.94700	0.49500
C	-8.28700	3.01100	1.21600
C	-8.29800	2.82400	2.73900
C	-6.84300	3.01200	0.69500
H	-10.86100	1.86800	1.84400
H	-8.75500	0.95200	0.76400
H	-8.97899	2.04800	-0.58700
H	-8.72800	3.99400	0.99100
H	-7.69200	3.59100	3.23200
H	-9.30700	2.87900	3.15700
H	-7.87700	1.84700	3.00800
H	-6.25000	3.80300	1.16300
H	-6.34700	2.05900	0.91400
H	-6.80700	3.16300	-0.38900
C	-0.51300	-0.58299	-0.08600

O	-0.66200	-0.14000	1.75400
C	0.98500	1.36400	0.77000
C	2.17300	1.63600	0.08300
C	0.25400	2.41200	1.33700
C	2.61700	2.95300	-0.03300
C	0.70000	3.72600	1.20800
C	1.88400	4.00100	0.52600
C	-1.40900	0.25600	-0.86500
C	-0.95900	1.42300	-1.51200
C	-2.77600	-0.06500	-0.89800
C	-1.86800	2.26700	-2.13600
C	-3.68700	0.80800	-1.48400
C	-3.23400	1.97800	-2.09700
C	0.48000	-0.04700	0.87900
H	2.73900	0.83000	-0.37200
H	-0.66000	2.19000	1.87100
H	3.54800	3.16200	-0.54400
H	0.11600	4.53500	1.63400
H	2.24000	5.02100	0.43600
H	0.09500	1.65800	-1.52200
H	-3.12100	-0.98200	-0.43600
H	-1.51300	3.16900	-2.62100
H	-4.74300	0.57199	-1.46800
H	-3.94600	2.66200	-2.54700
H	-0.73100	-1.63700	0.01300
H	1.26899	-0.75100	1.14500
C	-4.56900	-7.20200	0.90300
C	-3.36600	-6.25200	0.88600
C	-3.83500	-4.78700	0.81700
C	-2.40400	-6.61000	-0.24800
C	-2.71100	-3.75100	0.88100
H	-5.20400	-7.04800	0.02300
H	-2.82100	-6.37700	1.83600
H	-4.41200	-4.63800	-0.10500
H	-4.53200	-4.61200	1.64600
H	-1.51300	-5.98400	-0.23400
H	-2.07200	-7.65100	-0.17500
H	-2.88800	-6.48800	-1.22300
H	-3.12500	-2.74000	0.95800
H	-2.06800	-3.90600	1.75200
H	-2.06900	-3.78500	-0.00500
C	6.37700	1.94100	5.41800
C	5.74400	2.95200	6.35000
H	7.06200	1.29700	5.97900
H	5.17600	2.46000	7.14600
H	6.95500	2.42700	4.62399
H	5.62600	1.30600	4.94100
C	-2.74900	-2.09800	5.70000
C	-2.09000	-1.69500	4.38600
C	-0.87800	-2.54300	4.01499
O	-0.46000	-3.42100	4.79500
O	-0.32800	-2.28900	2.87700
H	-1.99800	-2.29400	6.46900
H	-2.79500	-1.74800	3.54800
H	-1.75400	-0.65100	4.41099
H	6.49400	3.59300	6.82600
H	5.04500	3.60800	5.81700
H	1.73600	9.91100	-0.09000
H	1.57600	7.43200	-3.71200
H	-2.43500	9.63400	1.32000
H	-1.86300	8.57199	2.59700
H	-4.51900	2.78900	6.26400
H	-4.95000	4.46000	5.88300
H	-11.18100	1.24000	0.22600
H	-11.05600	2.98400	0.48600
H	-9.14200	-1.23100	-4.34700
H	-9.88299	0.27000	-3.77200
H	-6.15900	-6.43400	-4.21400
H	-7.59500	-5.41000	-4.26499
H	-0.11900	-2.46400	-7.16700
H	-1.87200	-2.51900	-7.51300
H	0.23300	-6.43800	-4.69800
H	-1.53800	-6.48900	-4.51900
H	-5.18300	-7.04600	1.79600
H	-4.24800	-8.25000	0.90000
H	7.40300	-3.93800	5.29500
H	6.32200	-3.15900	6.45000
H	0.49000	5.52100	5.64300
H	0.71900	4.24099	6.86300
H	-7.53100	-2.23300	4.88199
H	-7.14500	-3.51400	3.71400
H	6.66300	-6.39700	-5.95100
H	4.94200	-6.22000	-5.56000
H	6.53600	-2.62800	-8.87299
H	4.93700	-2.01600	-8.46700
H	0.78800	-2.01200	9.37500
H	0.46600	-3.75500	9.55300
H	-3.36100	-3.00000	5.59800
H	-3.41000	-1.30100	6.05700
H	5.60200	4.92900	-4.68600
H	5.34300	6.03000	-3.33600
H	-0.58700	-1.07100	2.27199
H	-0.57599	-7.19400	4.42600

60A-TS-SS

O	0.95900	-1.61100	-1.75600
H	0.92500	-2.57100	-1.51200
H	0.33800	-1.58000	-2.52400
C	6.40100	-3.54300	5.41200
C	5.32100	-4.60799	5.19299
C	5.29900	-5.27700	3.80700
C	4.27700	-6.42100	3.79700
C	4.97700	-4.28899	2.67900
H	6.27400	-2.70000	4.72499
H	5.43200	-5.39100	5.95500
H	4.33600	-4.15400	5.36500
H	6.29700	-5.70300	3.61800
H	4.24300	-6.92000	2.82200
H	4.50800	-7.17500	4.55900
H	3.27300	-6.02900	4.00100
H	4.99400	-4.78000	1.70100
H	3.97300	-3.87200	2.81500
H	5.68500	-3.45800	2.63700
C	5.82100	-2.49600	-8.06600
C	6.38000	-1.68000	-6.89300
C	5.33400	-1.25699	-5.87700
C	4.48200	-2.17800	-5.24900
C	5.19800	0.09000	-5.51900

C	3.56400	-1.78600	-4.27600
C	4.28000	0.50100	-4.55500
C	3.48100	-0.44200	-3.90500
O	2.60000	0.01200	-2.95800
H	5.51300	-3.49600	-7.74300
H	7.16800	-2.25999	-6.39300
H	6.87000	-0.77700	-7.27800
H	4.54000	-3.23100	-5.50400
H	5.82800	0.83300	-5.99900
H	2.93900	-2.52100	-3.78000
H	4.18700	1.54600	-4.28400
H	2.18500	-0.74100	-2.44700
C	-1.11900	-2.34800	-6.74500
C	-1.35200	-0.98400	-6.08600
C	-0.45500	-0.82800	-4.87899
N	0.42800	0.18800	-4.87899
O	-0.56100	-1.62600	-3.93500
H	-1.21400	-3.14200	-6.00100
H	-1.20200	-0.16900	-6.80400
H	-2.38200	-0.92300	-5.72200
H	1.13999	0.24800	-4.14600
H	0.55700	0.74300	-5.70900
C	5.45500	4.98299	-3.61600
C	6.65100	4.33399	-2.90400
C	6.63400	2.79400	-2.92600
C	8.01900	2.21500	-2.62100
C	5.59800	2.22900	-1.94700
H	4.50900	4.49099	-3.37200
H	7.57500	4.69500	-3.37200
H	6.69700	4.66899	-1.86000
H	6.35400	2.47500	-3.94000
H	8.00000	1.12100	-2.64500
H	8.76500	2.55700	-3.34600
H	8.35900	2.52100	-1.62400
H	5.50100	1.14500	-2.04900
H	5.90000	2.44800	-0.91400
H	4.60400	2.65500	-2.10400
C	0.80800	7.02800	-3.05600
C	0.74900	7.85400	-1.77700
O	-0.30700	8.18600	-1.24100
C	1.15300	5.56300	-2.72400
C	1.23200	4.61100	-3.92600
C	1.65900	3.21700	-3.45300
C	-0.09000	4.52700	-4.69800
H	-0.16100	7.11700	-3.54400
H	2.11600	5.53900	-2.20000
H	0.41500	5.18000	-2.01300
H	2.00599	4.99600	-4.60799
H	1.84500	2.55200	-4.30100
H	2.57500	3.25000	-2.85700
H	0.88600	2.75900	-2.82900
H	-0.01000	3.81600	-5.52600
H	-0.89600	4.18200	-4.04100
H	-0.38900	5.49000	-5.12100
N	1.95900	8.16000	-1.22800
C	2.08700	8.88800	0.02800
C	3.52900	8.84900	0.54000
C	3.97500	7.47500	0.99400
C	4.80700	6.67400	0.20200
C	3.56100	6.97900	2.23900

C	5.23800	5.42500	0.65400
C	3.99000	5.73400	2.69400
C	4.84100	4.95700	1.90500
H	2.80000	7.87200	-1.70500
H	1.41300	8.43099	0.76000
H	4.20400	9.22600	-0.23900
H	3.60400	9.55000	1.37800
H	5.15000	7.04100	-0.76300
H	2.91200	7.58700	2.86400
H	5.89300	4.82400	0.03400
H	3.67200	5.37500	3.66600
H	5.19600	3.99700	2.26700
C	-2.74100	8.85000	2.00400
C	-3.48200	7.61900	1.47200
C	-2.54800	6.69100	0.69100
S	-3.48800	5.41000	-0.24500
C	-2.36700	5.16700	-1.66500
H	-3.39100	9.46100	2.63800
H	-3.94800	7.07000	2.29700
H	-4.29399	7.94200	0.81100
H	-1.96600	7.26400	-0.03500
H	-1.84400	6.20400	1.37700
H	-2.95800	4.75100	-2.48100
H	-1.94600	6.12700	-1.96900
H	-1.56700	4.46200	-1.43100
C	0.48700	4.44700	5.80600
C	1.38600	3.74500	4.78300
C	1.28200	2.24900	4.75300
C	0.35100	1.43900	5.36000
C	2.16100	1.37100	4.01800
C	1.69200	0.04100	4.20500
C	3.31200	1.58200	3.24500
N	0.58500	0.11500	5.01999
C	2.32399	-1.06500	3.62700
C	3.96200	0.48600	2.69100
C	3.46800	-0.82300	2.87400
H	-0.55400	4.12000	5.70200
H	1.16599	4.13600	3.77900
H	2.43299	4.01999	4.97800
H	-0.47300	1.69500	6.00800
H	0.12700	-0.68400	5.42700
H	3.68400	2.58700	3.08200
H	1.93400	-2.06599	3.76500
H	4.86300	0.63300	2.10200
H	3.99400	-1.65600	2.42499
C	0.69200	-2.95100	8.82300
C	1.92700	-3.32000	7.99100
C	1.71200	-4.66800	7.28900
C	2.25500	-2.23800	6.95900
H	-0.18900	-2.83600	8.18000
H	2.78000	-3.41900	8.67900
H	2.59600	-4.96300	6.71500
H	1.49200	-5.46500	8.00799
H	0.86600	-4.58600	6.59600
H	3.20600	-2.43600	6.45500
H	1.47800	-2.22400	6.19100
H	2.31800	-1.24200	7.41100
C	0.46900	-6.82400	4.36300
N	0.58800	-5.62700	3.53600
C	0.74800	-5.58100	2.19899

N	0.93300	-6.69000	1.46000
N	0.78300	-4.39700	1.58500
H	0.79800	-6.57000	5.37200
H	1.12300	-7.62000	3.99700
H	0.35200	-4.72600	3.97900
H	0.80600	-7.59900	1.87000
H	0.96300	-6.61200	0.43100
H	0.42400	-3.56800	2.07600
H	0.74100	-4.37000	0.55000
C	-4.22800	3.66300	5.65500
C	-4.29000	3.19900	4.19600
C	-4.01300	4.36000	3.24000
C	-3.31300	2.04199	3.94800
H	-3.22500	4.04399	5.89000
H	-5.30700	2.83100	3.99600
H	-4.03899	4.04500	2.19400
H	-4.74899	5.16300	3.36900
H	-3.02000	4.78700	3.43000
H	-3.36500	1.67900	2.91600
H	-2.27800	2.35400	4.13000
H	-3.52600	1.19400	4.61000
C	-7.58900	-2.52100	3.82900
C	-6.81200	-1.51200	2.98000
C	-6.58500	-1.97400	1.53000
C	-7.89600	-2.12200	0.74900
C	-5.61700	-1.03499	0.81000
H	-8.63599	-2.59700	3.52200
H	-7.32600	-0.53900	2.97400
H	-5.83200	-1.33500	3.44200
H	-6.11400	-2.96700	1.57000
H	-7.70400	-2.48500	-0.26400
H	-8.58100	-2.82700	1.23000
H	-8.41400	-1.15900	0.66300
H	-5.38700	-1.38100	-0.20000
H	-6.03500	-0.02300	0.72500
H	-4.67500	-0.95700	1.36300
C	5.94600	-6.31200	-5.14700
C	6.29700	-5.14600	-4.22000
C	5.29200	-4.89300	-3.13500
C	4.20000	-5.65600	-2.78700
C	5.27400	-3.75100	-2.25599
C	4.13400	-3.88400	-1.41000
C	6.08700	-2.61200	-2.13899
N	3.51200	-5.06600	-1.74400
C	3.78900	-2.90000	-0.47500
C	5.74800	-1.63700	-1.21200
C	4.60700	-1.77800	-0.39500
H	5.96300	-7.26100	-4.60200
H	7.28700	-5.32800	-3.77700
H	6.41300	-4.22900	-4.81400
H	3.84300	-6.58700	-3.20200
H	2.64200	-5.43300	-1.36700
H	6.95400	-2.48800	-2.78000
H	2.92000	-3.01500	0.16400
H	6.36800	-0.75300	-1.10800
H	4.37100	-0.99800	0.32000
C	-0.58500	-6.42400	-3.98600
C	-0.54000	-5.18900	-3.06900
C	0.35800	-5.26200	-1.82400
O	0.85400	-6.36000	-1.45400
O	0.50900	-4.17199	-1.17000
H	-0.48600	-7.32900	-3.38100
H	-0.27800	-4.27700	-3.61500
H	-1.53800	-4.99600	-2.65800
C	-7.00100	-6.06500	-3.62500
C	-6.50000	-5.30500	-2.38499
C	-5.69800	-4.05600	-2.69500
C	-6.31200	-2.79700	-2.71800
C	-4.31900	-4.12100	-2.94000
C	-5.56700	-1.63700	-2.93500
C	-3.56800	-2.96500	-3.16400
C	-4.19200	-1.71600	-3.14900
H	-7.62000	-6.92000	-3.33700
H	-7.35700	-5.02799	-1.76100
H	-5.88500	-5.97900	-1.77900
H	-7.38100	-2.72300	-2.54300
H	-3.82400	-5.08900	-2.93100
H	-6.05900	-0.67000	-2.91100
H	-2.50000	-3.02000	-3.33400
H	-3.60400	-0.81400	-3.28300
C	-8.92600	-0.22800	-3.95300
C	-8.05300	0.55200	-4.93800
C	-7.80500	2.02300	-4.55700
C	-7.13500	2.15700	-3.18600
C	-6.96200	2.71700	-5.63200
H	-8.43500	-0.34600	-2.98100
H	-7.08400	0.04400	-5.04100
H	-8.51800	0.52400	-5.93200
H	-8.78300	2.52500	-4.51100
H	-6.92400	3.20500	-2.94900
H	-7.75600	1.75500	-2.37900
H	-6.18400	1.61400	-3.17100
H	-6.81500	3.77800	-5.40100
H	-5.97000	2.25300	-5.70300
H	-7.43200	2.64700	-6.61800
C	-10.63700	2.01700	0.78500
C	-9.12500	1.98600	0.53700
C	-8.32600	3.07700	1.27400
C	-8.34700	2.87600	2.79600
C	-6.88100	3.13600	0.76600
H	-10.88300	1.85500	1.83800
H	-8.71899	1.00499	0.81900
H	-8.94699	2.08599	-0.54200
H	-8.80200	4.04500	1.05200
H	-7.77900	3.66300	3.30200
H	-9.36200	2.88600	3.20100
H	-7.89000	1.91400	3.06100
H	-6.31700	3.94200	1.24500
H	-6.35600	2.19700	0.98100
H	-6.84700	3.30300	-0.31500
C	-1.15100	-0.37300	0.07400
O	-1.45800	-0.18000	1.45500
C	0.76100	1.34900	0.33700
C	2.15899	1.52500	0.20000
C	-0.07199	2.49200	0.48300
C	2.69900	2.80400	0.16600
C	0.49000	3.75600	0.50400
C	1.87400	3.91500	0.33500
C	-1.96700	0.49100	-0.85400
C	-1.45300	0.82300	-2.11100

C	-3.22800	0.95400	-0.48300	H	-1.92000	-2.31700	6.38300
C	-2.20900	1.58100	-3.00200	H	-2.96500	-1.72800	3.55500
C	-3.98500	1.71800	-1.37300	H	-1.91700	-0.58700	4.34500
C	-3.48200	2.02400	-2.63700	H	6.50600	3.60300	6.80700
C	0.26000	0.02100	0.29400	H	5.05199	3.61500	5.80400
H	2.79300	0.65600	0.07199	H	1.73700	9.91900	-0.10000
H	-1.13399	2.36600	0.63800	H	1.57500	7.44100	-3.72100
H	3.76500	2.93700	0.04500	H	-2.39100	9.47300	1.17500
H	-0.14399	4.62300	0.65400	H	-1.85800	8.57799	2.59100
H	2.31000	4.90800	0.34900	H	-4.43300	2.83700	6.34400
H	-0.46300	0.49400	-2.38899	H	-4.93900	4.46500	5.88100
H	-3.60600	0.72600	0.50400	H	-11.14700	1.24400	0.20300
H	-1.79500	1.82800	-3.97400	H	-11.05100	2.98700	0.49000
H	-4.96200	2.07600	-1.07200	H	-9.14200	-1.23100	-4.33200
H	-4.07500	2.61500	-3.32900	H	-9.88100	0.27500	-3.77100
H	-1.22000	-1.42400	-0.21600	H	-6.16100	-6.43500	-4.22100
H	0.94200	-0.78000	0.55800	H	-7.59200	-5.40400	-4.26600
C	-4.55999	-7.19600	0.89700	H	-0.12600	-2.41200	-7.19700
C	-3.37700	-6.22200	0.83900	H	-1.87400	-2.50999	-7.52100
C	-3.87600	-4.77500	0.67600	H	0.23300	-6.41900	-4.71200
C	-2.40000	-6.63000	-0.26700	H	-1.53600	-6.48200	-4.52799
C	-2.77900	-3.71200	0.61200	H	-5.17300	-7.04100	1.79100
H	-5.20600	-7.08300	0.01900	H	-4.21400	-8.23600	0.91700
H	-2.83600	-6.28300	1.79700	H	7.41600	-3.92900	5.27700
H	-4.48800	-4.71200	-0.23100	H	6.33800	-3.14000	6.42800
H	-4.54900	-4.54699	1.51400	H	0.50000	5.52800	5.63500
H	-1.51300	-6.00000	-0.27300	H	0.80400	4.24200	6.83400
H	-2.06599	-7.66500	-0.13900	H	-7.57200	-2.25599	4.89200
H	-2.87400	-6.55900	-1.25200	H	-7.13500	-3.50900	3.71200
H	-3.21800	-2.71400	0.52800	H	6.66200	-6.39000	-5.96900
H	-2.14900	-3.72200	1.50500	H	4.94299	-6.21000	-5.57600
H	-2.12000	-3.85200	-0.25000	H	6.53200	-2.61600	-8.89000
C	6.38800	1.94900	5.40200	H	4.93400	-2.00300	-8.47899
C	5.75600	2.96100	6.33500	H	0.83800	-2.01600	9.37299
H	7.07400	1.30500	5.96100	H	0.48400	-3.75000	9.54200
H	5.19299	2.46800	7.13400	H	-3.34900	-2.99500	5.59200
H	6.96500	2.43500	4.60600	H	-3.37200	-1.31900	6.13600
H	5.63600	1.31200	4.92800	H	5.60100	4.93900	-4.70200
C	-2.73600	-2.09300	5.69300	H	5.35800	6.04200	-3.35000
C	-2.19899	-1.64600	4.33500	H	-1.16100	-1.03899	1.97800
C	-0.96700	-2.41699	3.85800	H	-0.56299	-7.18700	4.41600
O	-0.36400	-3.18600	4.63900				
O	-0.59100	-2.19499	2.64900				
